# The Middle-to-Upper Paleolithic transition occupations from Cova Foradada (Calafell, NE Iberia)

**DOI:** 10.1371/journal.pone.0215832

**Published:** 2019-05-16

**Authors:** Juan I. Morales, Artur Cebrià, Aitor Burguet-Coca, Juan Luis Fernández-Marchena, Gala García-Argudo, Antonio Rodríguez-Hidalgo, María Soto, Sahra Talamo, José-Miguel Tejero, Josep Vallverdú, Josep Maria Fullola

**Affiliations:** 1 SERP, Departament d’Historia i Arqueologia, Universitat de Barcelona, Barcelona, Spain; 2 Àrea de Prehistòria, Universitat Rovira i Virgili (URV), Tarragona, Spain; 3 Institut Català de Paleoecologia Humana i Evolució Social (IPHES), Tarragona, Spain; 4 Complutense University, Prehistory, Ancient History and Archaeology Department, Madrid, Spain; 5 IDEA (Instituto de Evolución en África), Madrid, Spain; 6 Department of Anthropology and Archaeology, University of Calgary, Calgary, Canada; 7 Max Planck Institute for Evolutionary Anthropology, Department of Human Evolution, Leipzig, Germany; 8 CNRS, ArScan, UMR-7041, Ethnologie préhistorique, Nanterre, France; Universita degli Studi di Ferrara, ITALY

## Abstract

The Middle-to-Upper Paleolithic transition in Europe covers the last millennia of Neanderthal life together with the appearance and expansion of Modern Human populations. Culturally, it is defined by the Late Middle Paleolithic succession, and by Early Upper Paleolithic complexes like the Châtelperronian (southwestern Europe), the Protoaurignacian, and the Early Aurignacian. Up to now, the southern boundary for the transition has been established as being situated between France and Iberia, in the Cantabrian façade and Pyrenees. According to this, the central and southern territories of Iberia are claimed to have been the refuge of the last Neanderthals for some additional millennia after they were replaced by anatomically Modern Humans on the rest of the continent. In this paper, we present the Middle-to-Upper Paleolithic transition sequence from Cova Foradada (Tarragona), a cave on the Catalan Mediterranean coastline. Archaeological research has documented a stratigraphic sequence containing a succession of very short-term occupations pertaining to the Châtelperronian, Early Aurignacian, and Gravettian. Cova Foradada therefore represents the southernmost Châtelperronian–Early Aurignacian sequence ever documented in Europe, significantly enlarging the territorial distribution of both cultures and providing an important geographical and chronological reference for understanding Neanderthal disappearance and the complete expansion of anatomically Modern Humans.

## Introduction

The Middle-to-Upper Paleolithic transition in Europe (ca. 45–35 ka cal BP) covers the last millennia of Neanderthal presence in the fossil record, together with the appearance of anatomically Modern Human populations. In SW Europe this is broadly represented by the Late Middle Paleolithic succession, the so-called transitional assemblages, including the Châtelperronian, Neronian, and Uluzzian, and the Protoaurignacian and Early Aurignacian assemblages. While there is general agreement that Neanderthals are associated with the Late Middle Paleolithic and Modern Humans with the Aurignacian, there has been intense debate about who was responsible for transitional industries such as those of the Châtelperronian and the Uluzzian [1–8]. Inferences from paleoanthropological [9–13], technological [14–17], and paleo-proteomic studies [18] seem to point out that Neanderthals were the makers of Châtelperronian artifacts. Notwithstanding, this statement is far from being universally accepted amongst researchers [1,19] and the debate continues. Be that as it may, during the Transition, Neanderthals and Modern Humans appear to have co-inhabited geographical zones over a fairly narrow time span, opening up the possibility of various kinds of interaction, ranging from population replacement [20–23], and biological assimilation [24–29], to indirect contact and technological diffusion between the species [30,31].

The Iberian Peninsula is the southern boundary of the Transition in SW Europe. This geographical area has played a key role in the aforementioned debate, mainly due to the alleged existence of a dual demographic dynamic [29,32,33]. Iberia is thus of particular interest for understanding species extinction and expansion processes, and a suitable area for testing the hypothesis on interspecific competitiveness, coexistence, admixture, and exclusion [34]. The Cantabrian façade and Pyrenees (northern Spain) aggregate the entire set of Iberian Châtelperronian, Protoaurignacian, and Early Aurignacian sites, with the exception of Abric Romaní and Arbreda Cave (Catalonia) [35]. Solid evidence for the Iberian Châtelperronian comes from Labeko Koba, Ekain, and Aranbaltza, in the Basque Country, and Cueva Morín, in Cantabria [36–40]. The most reliable chronology for the Iberian Châtelperronian is provided by Labeko Koba, 42.5–41.6 14C ka cal BP [41], while the oldest Protoaurignacian/Early Aurignacian assemblages date from as early as 43.3–40.5 14C ka cal BP [41,42], or 42.8–41.3 14C ka cal BP [43] at Isturitz, in the French Pyrenees.

Conversely, no evidence of these cultures has ever been found south of the Ebro River basin, where the local Late Middle Paleolithic is replaced by the Evolved Aurignacian, at the earliest during GI8, ca. 37.5 ka cal BP [29]. Assuming that Neanderthals were responsible for the Châtelperronian and Modern Humans for the Protoaurignacian industries, there is a millenary gap between Modern Humans first peopling the northern area and their expansion over the entire Iberian Peninsula. A by-product of this situation is the debate concerning the survival of Late Middle Paleolithic groups in the south, emerging through the arguments of the so-called Ebro Frontier Model [29,33,44,45].

Here we present, for the first time, the archaeological sequence from Cova Foradada on the Mediterranean coast of Catalonia (NE Iberia). Our results indicate that the site represents the southernmost European manifestation of both Châtelperronian and Early Aurignacian occupations. Located far from the Cantabrian-Pyrenean influence and close to the Ebro basin boundary, Cova Foradada can be regarded as an important geographical hinge between territorial dynamics on the Iberian Peninsula and is a key site for understanding the southern expansion of the Châtelperronian and Early Aurignacian in Europe.

## Methods

### Site and excavation process

**Fig 1:**
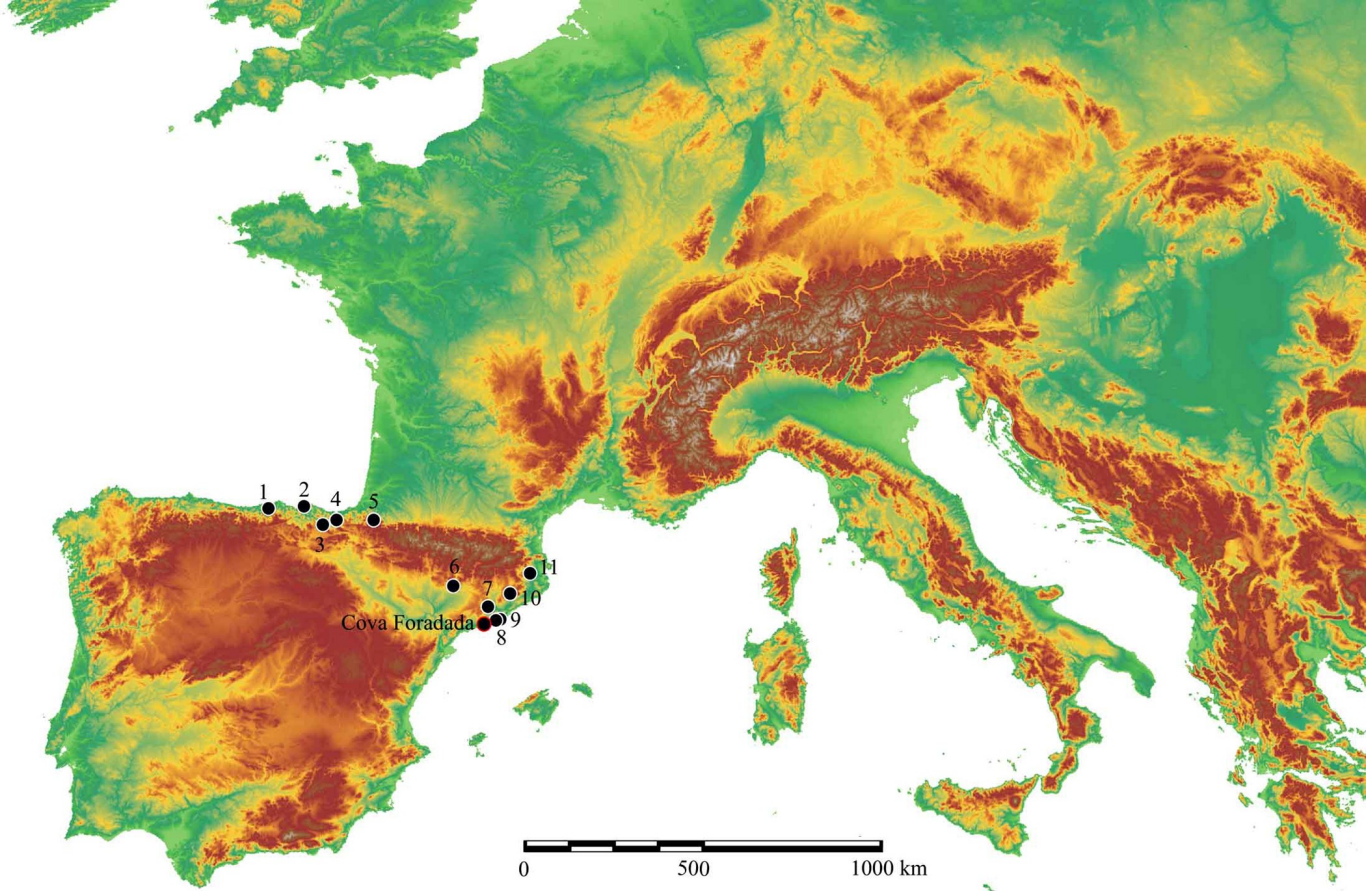
Location of Cova Foradada. Location of Cova Foradada and the main sites in Iberia containing transitional or earliest Upper Paleolithic assemblages, as referred to in the text. 1-Cueva Morín, 2-Aranbaltza, 3-Labeko Koba, 4-Ekain, 5-Isturitz, 6-Cova Gran, 7-Abric Romaní, 8-Cova del Gegant, 9-Canyars, 10-Teixoneres, 11-L’Arbreda.

**Fig 2:**
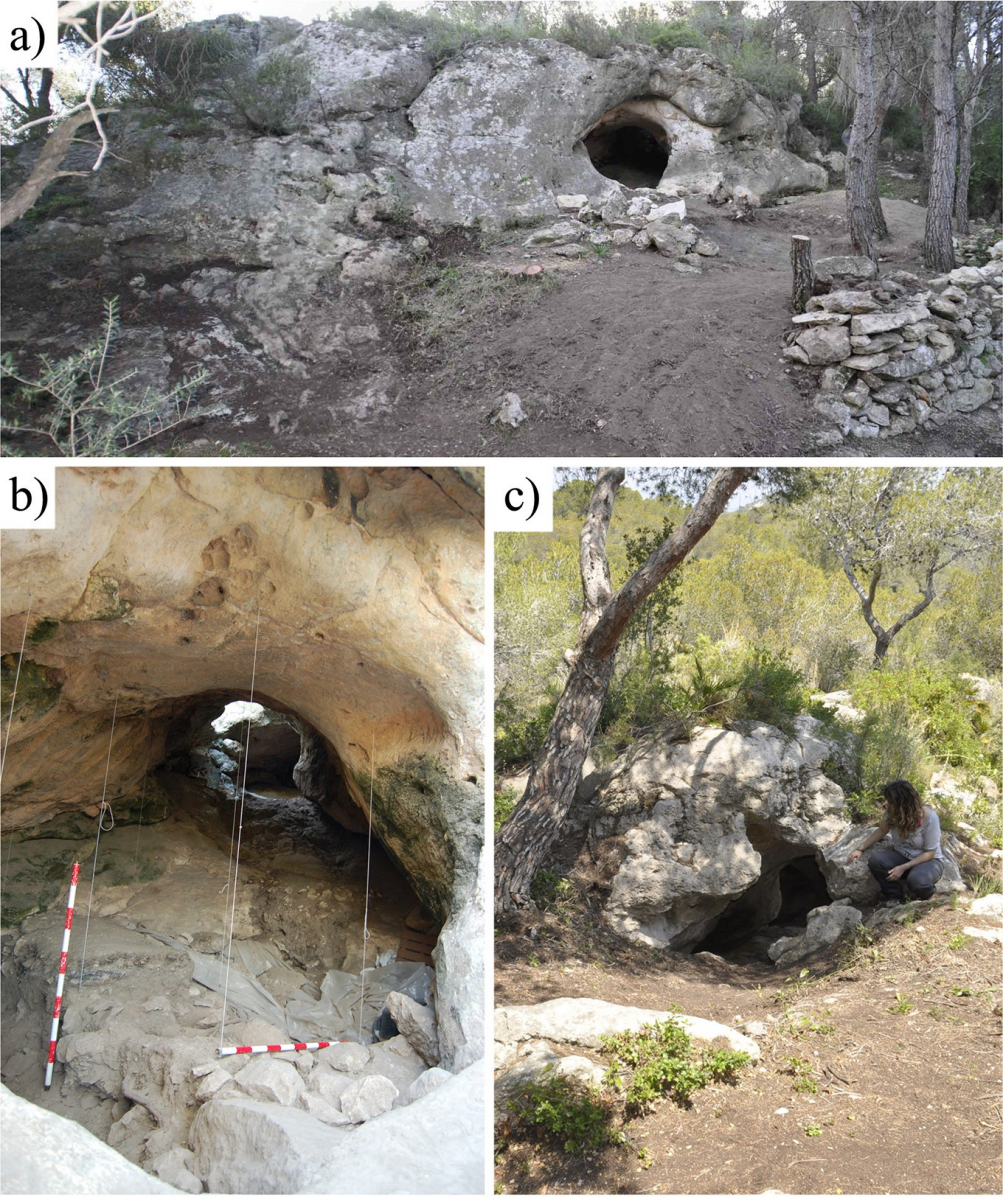
View of the different parts of Cova Foradada. a) General view of the Lower Entrance of the cave and external terrace prior to its excavation in 2014; b) interior of the cave from the Lower Entrance during the excavation of Layer I in 2006, the excavation hall; c) view of the Upper Entrance of the cave.

**Fig 3:**
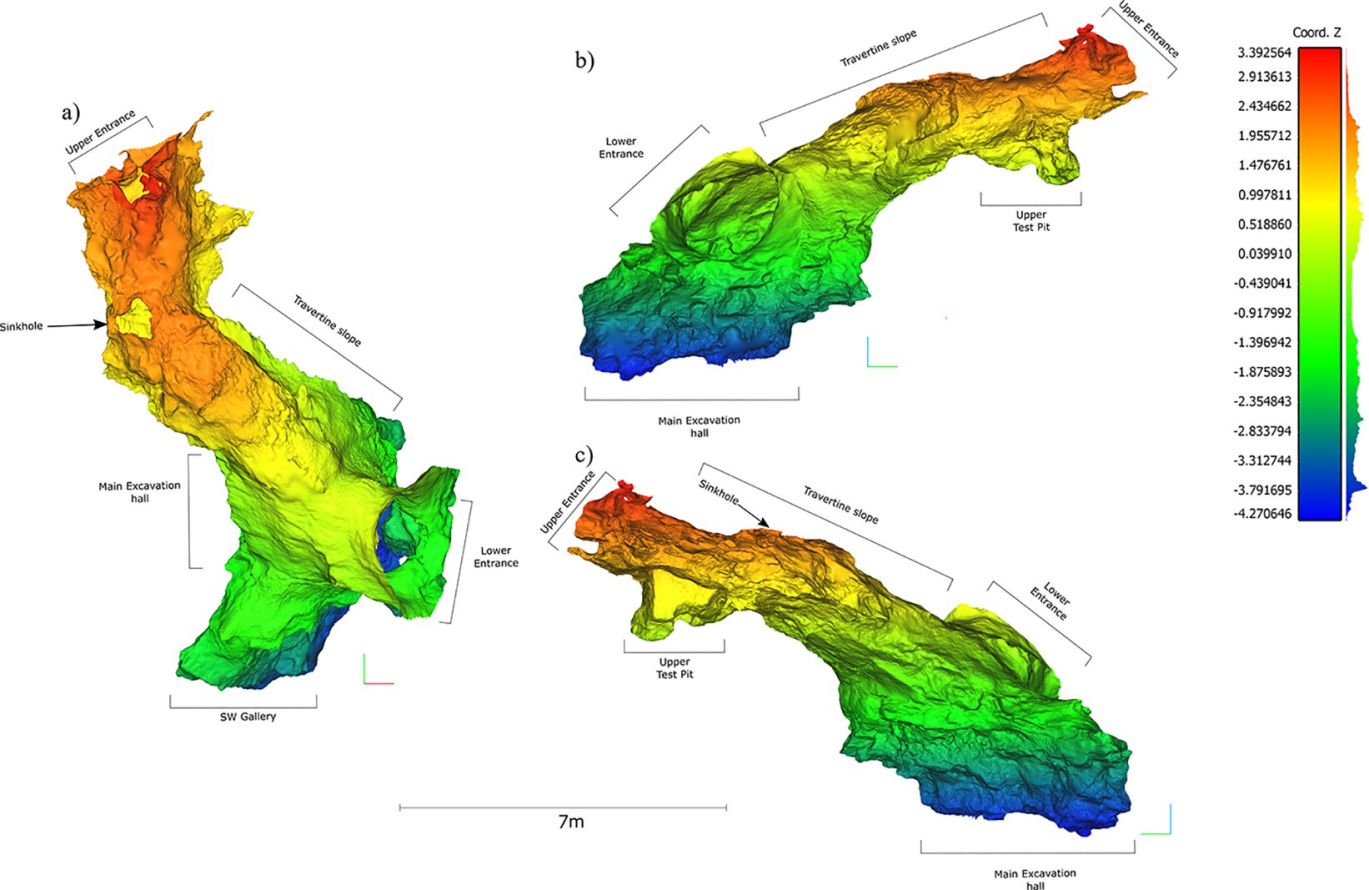
Digital elevation model of the interior part of the karstic development. a) Upper, b) front, and c) back views of the Cova Foradada morphology from the photogrammetric 3D model of the cave. The external structure of the cave has been removed from the model to show the internal morphology of the karst system. Digital elevation model created in CloudCompare v2.1.0 alpha.

Cova Foradada is on the Mediterranean coast of NE Spain, near the village of Calafell (Tarragona) at UTM (ETRS89) 381027.6–4562447.9, and 110 m.a.s.l. (Fig 1), in the Coastal Range of the Penedès region. The cave is a small karstic tunnel at the top of L’Escarnosa hill, 1.8 km from the current coastline. Geologically, the cavity is developed within Serravallian–Tortonian (Miocene) calcarenite formations, in discordant contact with Valanginian–Barremian (Lower Cretaceous) limestones and dolomites. The present morphology of the cave is defined by a circular entrance yielding direct access to the excavation hall of ca. 14 m^2^, from where a 7 m long travertine formation generates an ascending platform reaching an upper and smaller entrance (Fig 2). The original shape and development of the karst complex remain unknown (Fig 3 and Fig A in S1 Supporting Information), but a test-pit dug into the travertine platform close to the upper entrance has yielded additional, undated, Upper Paleolithic materials.

The cave’s archaeological potential was discovered in 1997, thanks to the accidental find of several Late Neolithic human bones by hikers. Since then, 11 archeological field-seasons have been undertaken. The first campaign was carried out under the Generalitat de Catalunya’s rescue excavation plan, to assess the potential and viability of the site for further research. Since 1999, it has been included in various research programs and projects at Barcelona University, and has been developed under a perspective of archaeological investigation. All the permits necessary for fieldwork activities have been obtained from the Generalitat de Catalunya’s Ministry of Culture and Calafell town council.

**Fig 4:**
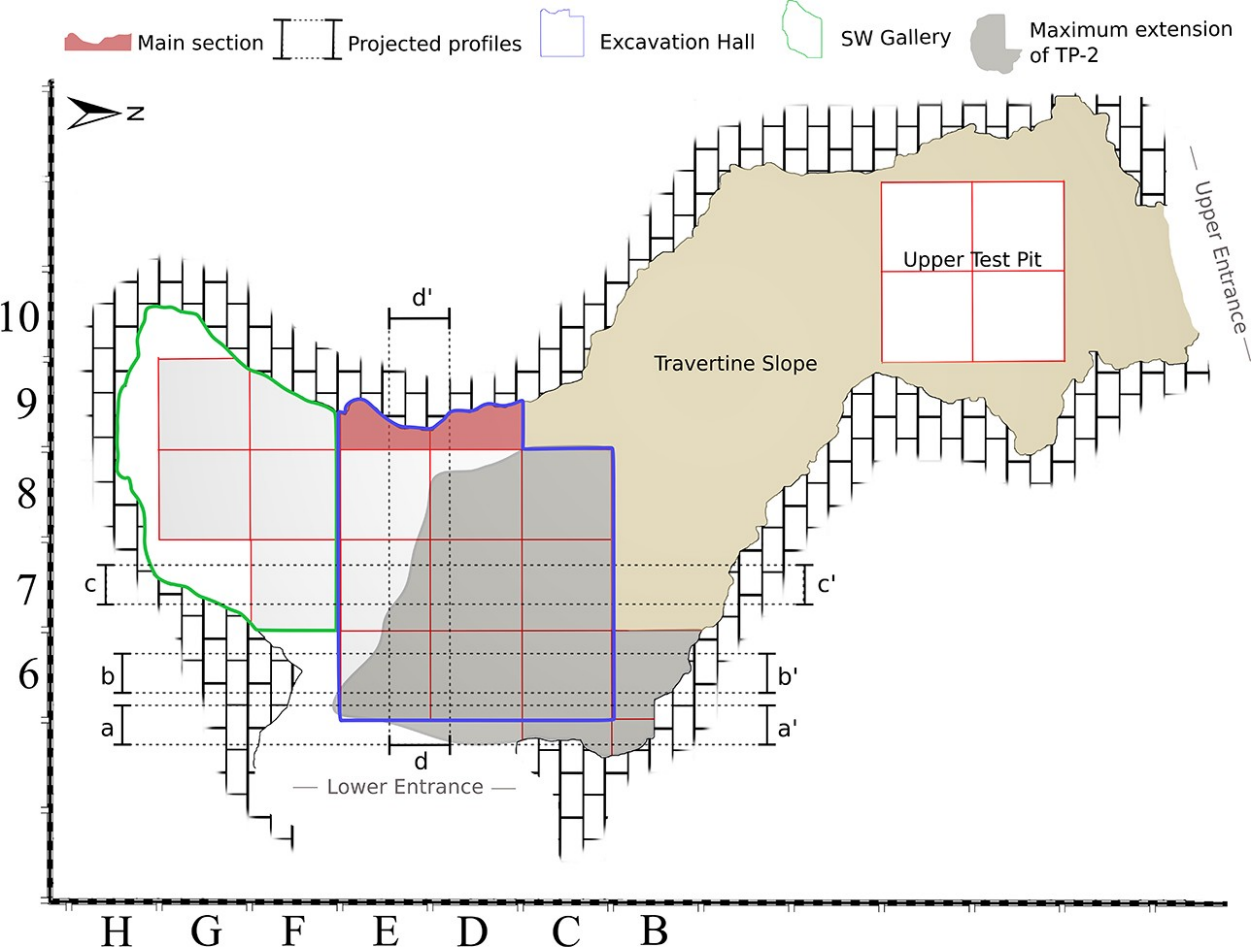
Plain view of Cova Foradada. Plain view of the cave indicating the different sectors mentioned in the text, including the excavation area (square), the travertine slope, and the upper test pit. Various features, such as the location of the main section, the position of the projected profiles, the location of the SW Gallery, and the Travertine Platform 2 (TP-2), are also indicated in the figure.

The main excavation tasks have been carried out in the excavation hall, where the stratigraphic sequence has been defined (Fig 4). The initial work, up to 2012, primarily focused on excavating the uppermost Late Neolithic layers [46–48]. The Upper Pleistocene sequence was documented and excavated between 2013 and 2017. In the 2014 fieldwork season, the external terrace of the lower entrance was also excavated, in an attempt to find preserved stratigraphy and occupations outside the cave. A 40 cm thick carbonated layer was documented below the superficial units and above the bedrock, but this contained no archaeological evidence (Figs AM to AP in S1 Supporting Information). In 2017, the same process was repeated, outside the upper entrance and documenting a large rockfall covered by slope deposits that probably sealed a wider cave entrance (Figs AS to AU in S1 Supporting Information). This excavation will continue over the coming years, in order to determine the possible relationship between the upper entrance and the remains documented below the travertine slope, in the upper test pit. Furthermore, this future work will be aimed at understanding the formation of the karst system.

**Fig 5:**
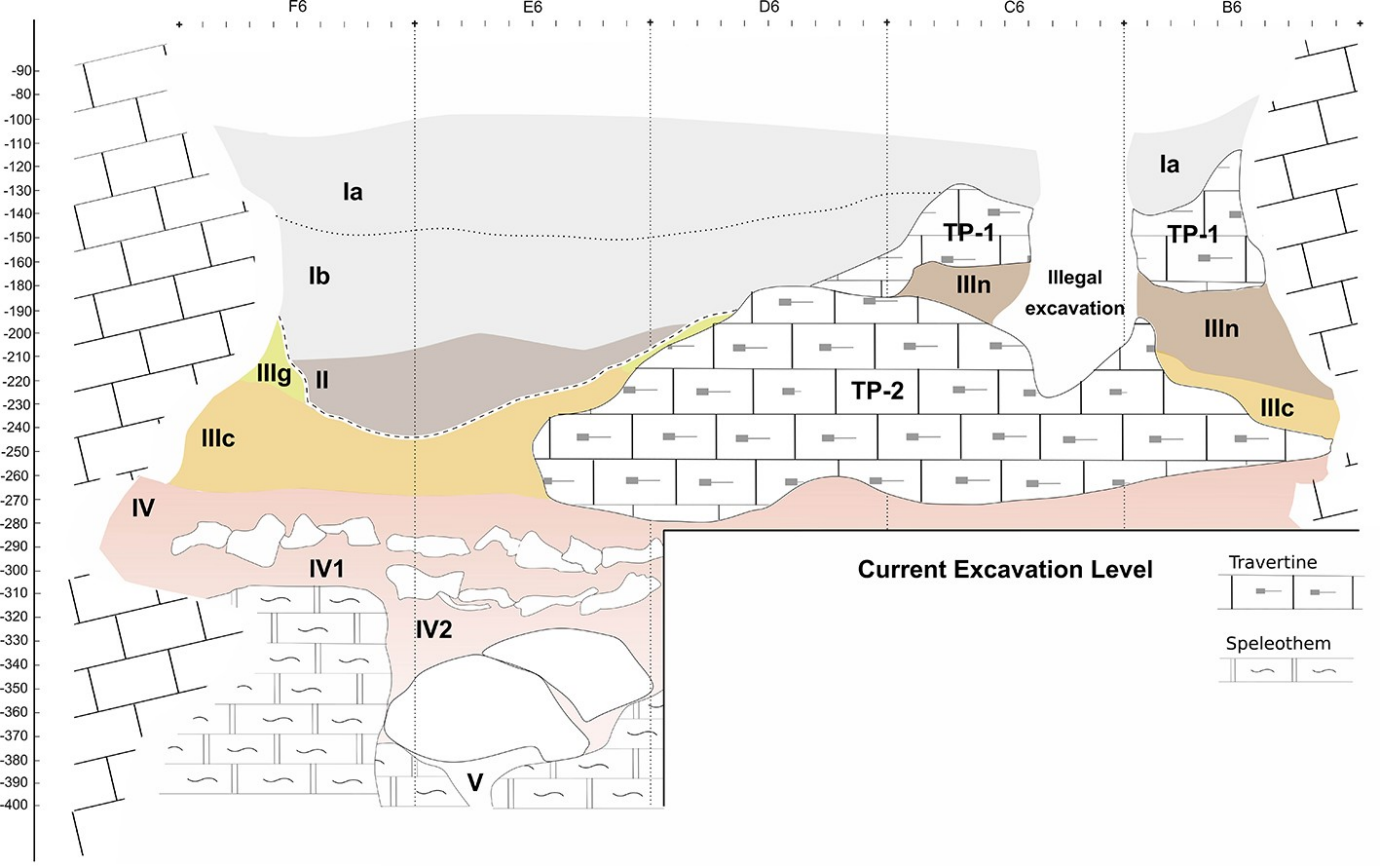
Stratigraphic reconstruction. Reconstruction of the stratigraphic succession at the site, based on field maps and the distribution of material along line y = 6. Black dashed line between layers II, IIIg, and IIIc indicates the basal part of the erosive discontinuity affecting the top of Unit III.

The Pleistocene lithostratigraphic sequence comprises three major units (III, IV, and V), defined based on common lithological and sedimentary traits and excavated following their natural orientation and slope. Within Unit III, several archeological layers (IIIn, IIIg, and IIIc) have been established, based on both sedimentary changes and differential preservation through the cave. The homogeneity of the sedimentary characteristics in Unit IV does not enable the definition of different layers, but two continuous lines of stones and travertine-crust fragments facilitated the definition of three sublayers within this unit (IV, IV1, and IV2). No clearly sterile layers between units or layers have been documented in the sequence, so the distribution of archaeological and paleontological remains is continuous. In the northern sector of the excavation hall, a thick travertine platform (TP-2) separates Unit III, overlying the platform, from Unit IV, below the platform. In the rest of the cave, these two units are in direct contact. The small size of the cave, together with the low compaction level of sediments, prevents large reference sections being maintained during the excavation. Uniquely, a small section of the Pleistocene layers was preserved in the E9 –D9 squares (Fig 4) to allow possible future sampling. The stratification preserved in this section is only partially representative of the general stratigraphy, as neither travertine platforms nor sublayers IV1 and IV2 are represented (Fig 5).

All the archaeological remains were individually recorded with a total station, using X-Y Cartesian coordinates and depth, or Z values, and individually bagged. A 20 mm cut-off length was established for recording non-identifiable bone or lithic fragments, and all rocks larger than 100 mm were also registered, including their upper and lower depth values. The sediment from each layer was kept in bags for 1 m^2^ units, with depths of 5 cm. Afterwards, this was transported for water screening using a sieving column with meshes of 0.5, 0.1, and 0.05 cm, and sorted to recover any small mammal remains, in addition to all lithic or bone fragments missed during the excavation.

Evidence of burrowing by small mammals, mainly rabbits, has been documented throughout the Cova Foradada sequence (Figs M, U and AJ in S1 Supporting Information). The Holocene layers have been heavily bioturbated, while the Pleistocene layers are significantly less affected. All the burrows identified during the excavation were mapped, isolated, and completely excavated prior to the excavation of the *in situ* areas. The burrow infills, and any archaeological materials found within these sediments, were isolated to avoid mixing with stratigraphically reliable assemblages, and the burrow-infill materials were excluded from the spatial analysis of the distribution of remains.

During the work on Units III and IV, the excavation surface gradually increased due to a progressive regression of the cave walls (Figs U and Z in S1 Supporting Information). This effect was particularly significant in the SW sector of the cave where almost three new lines of excavation squares were present in layer IIIc compared to layer I (squares G-H/8-9-10 or “SW Gallery”, Fig 4). This expanded area was excavated following the same general criteria as used in the rest of the dig; however, the narrowness of the space and the high number of large boulders made the stratigraphic correlation between this zone and the excavation hall unclear. Sediment and material subsidence from the upper layers were also identified close to the walls in the G-H area, and the stratigraphic assignation of a considerable proportion of the materials recovered is considered unreliable (Figs S, T and AB in S1 Supporting Information).

### FTIR analysis

In order to characterize possible fire-related archaeological features, FTIR analysis was carried out to determine the gross mineral components of the sediments, using a Fourier Jasco FT/IR-600 PLUS spectrometer. Infrared spectra were collected between 4000 and 400 cm^-1^ at a 4 cm^-1^ wavelength range resolution using the KBr method [49]. The use of FTIR to identify traces of combustion is based on the characterization of thermally altered clay—thermal impact [50], and the determination of ash in the form of pyrogenic calcite—combustion residue [51,52]. Wood ash is usually formed from calcium oxalate crystals (CaC_2_O_4_) found on the wood. When it is heated to around 500°C, the calcium oxalate decomposes to calcite with pyrogenic origin (CaCO_3_) through the release of carbon monoxide (CO).

Mineralogical compositions were interpreted based on a reference library [53]. To assess the geogenic or pyrogenic origin of the calcite, the infrared grinding curve method, based on the ratio measured between the 1420 and 713cm^-1^ peaks of the calcite, was used. These peaks are normalized using a third (874 cm^-1^), to correct any possible modification of the peaks by differential grinding during the sample preparation process.

Thermally altered clay was identified according to specific absorption peaks in the clay spectrum [50] and compared to heating experiments using the same sediments from Cova Foradada.

### Zooarchaeology and taphonomy

The faunal remains were studied following standardized zooarcheology methods techniques [54,55]. Three measurements were used to quantify faunal abundance: Number of Specimens (NSP), Number of Identified Specimens (NISP), and Minimum Number of Individuals (NMI) [56]. To assess the character of the fragmentation, fracture planes were analyzed following the criteria of Villa and Mahieu [57]. In the case of leporids, we followed the breakage patterns described in Lloveras et al. [58] and Cochard et al. [59]. Bone surface modifications were identified using a binocular microscope at x10 to x40 magnification, according to the recommendations of Blumenschine [60,61], and using the specific diagnostic criteria of Blumenschine and Selvaggio [62], Fisher [63], Domínguez-Rodrigo et al. [64], Shipman and Rose [65], Behrensmeyer [66], Binford [67], Stiner et al [68], and Fernández-Jalvo and Andrews [69]. The frequency of the modifications was calculated with respect to NSP. The malacofaunal remains were taxonomically identified following De Bruyne, Pope and Goto [70,71], and Appletans et al. [72].

### Radiocarbon dating

Charcoal, seashells, and bones were chosen for dating, depending on the layer. For layer IIIn, a specimen of *Homalopoma sanguineum* was used. For layers IIIc and IV, charcoal was selected since this was the most abundant occupation-related datable remain. In the case of layer IIIc, samples were individually hand-collected from the main section, selecting charcoal fragments according to the different combustion features observed. Independently, two pieces of charcoal from the “SW gallery” squares F-G/9 were also selected for stratigraphic control purposes. For layer IV, in the absence of clear combustion features, isolated charcoal fragments, individually recorded, were selected. In this case, spatial projections of the material distribution were used to select the samples most suitable for dating. When the charcoal was large enough, it was split into two subsamples for contrasting purposes, and, in three cases, these subsamples were submitted for dating to two different laboratories. In each case, one was dated in Beta Analytic labs using the AAA pretreatment method [73], while the other was analyzed in the Oxford Radiocarbon Accelerator Unit using ABOX-sc [74]. ABOX-sc is claimed to be more efficient in removing contaminants from the samples, therefore producing significantly older dates [75]. Finally, a preliminary attempt was made to date bone. In the absence of anthropogenically modified bones, unmodified ungulates, carnivores, and carnivore-modified bones were selected, to look for different patterns in the hominin-carnivore use of the cave, as well as the time-range represented by each lithostratigraphic unit. All the bone samples were pretreated in the Department of Human Evolution at the Max Planck Institute for Evolutionary Anthropology (MPI-EVA, Leipzig, Germany) using the method described in Talamo and Richards [76]. Unfortunately, the poor collagen preservation has, thus far, only allowed us to obtain one reliable date deriving from a lynx remain. This sample was sent to the Mannheim AMS laboratory (Lab Code MAMS) where it was graphitized and dated [77].

## Results

### Stratigraphy

**Fig 6:**
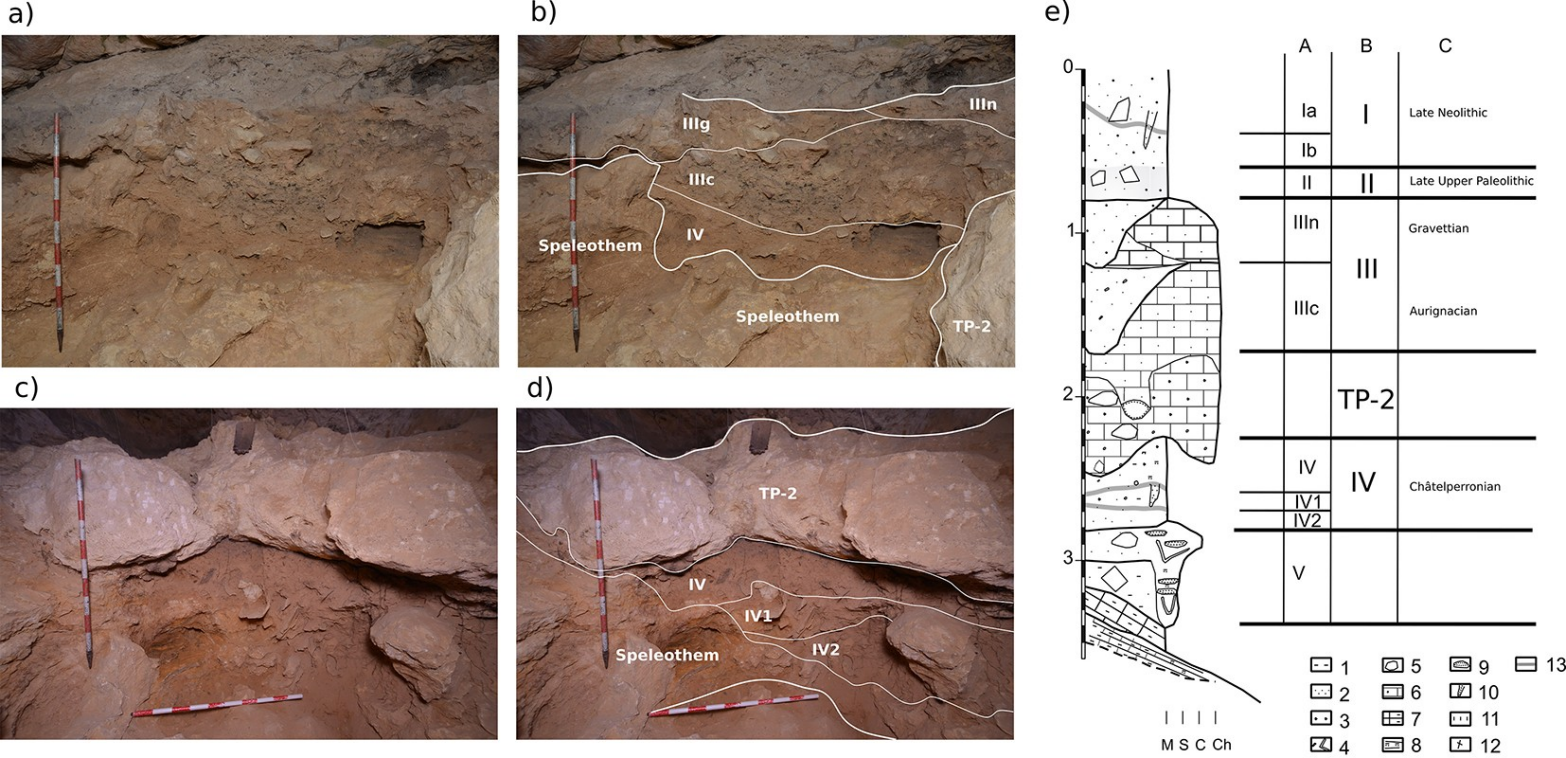
Stratigraphic succession. Stratigraphic succession at different locations within the site: a) and b) E9-D9 section where Unit III and Unit IV are stratigraphically continuous and are directly in contact. Layer IIIn has been almost completely eroded; c) and d) D6-7 section where TP-2 separates Unit III and Unit IV; e) lithostratigraphic column from the E6-D6 E-W section. Lithology: M = muds; S = sands; C = cobbles and boulders; Ch = chemical deposits. Symbols: 1, mud; 2, sand; 3, pebbles; 4, cobble and boulder slabs; 5, boulder; 6, calcarenite; 7, calcilutite and marls; 8, black impregnations; 9, cryptokarst; 10, root casts; 11, black cryptocrystalline impregnations; 12, massive; 13, archeological sublayers. Columns: A, archaeological layers; B, lithostratigraphic units; C, cultural attribution.

Work in the main excavation hall uncovered a stratigraphic sequence of ca. 2.5 m from the uppermost Late Neolithic layers to the basal speleothems. This stratigraphic succession contained four major lithostratigraphic units and ten archeopaleontological layers (see Fig 5, Fig 6 and S1 Fig). From top to bottom these were:

-Unit I. The uppermost unit of the sedimentary infill, comprising breccias of calcarenite cobbles and boulders with dusty organic sand and pebbles, and including an important presence of charcoal and ashes in a tabular, massive stratum. Unit I was intensively affected by bioturbation, mainly burrowing and root growth, but also, until recently, by anthropic activities. Subdivided into two archaeological sublayers (Ia and Ib) based on its integrity, the layer was dated as mid-Holocene, with a Late Neolithic collective burial.

-Unit II. A small lenticular stratum of brownish sandy clays containing abundant calcarenite cobbles and boulders from the collapse of the cave roof. Severely affected by trampling, bioturbation, and sediment removal during the latest funerary activities. The original deposition was associated with the Late Glacial-Early Holocene.

-Travertine Platform 1 (TP-1). TP-1 is a small relic of a larger, soft (tufa-like) travertine deposit. Identified only in the NE area of the excavation. Stratigraphically, TP-1 overlies Unit III and probably sealed it. During the excavation, only some remnants of TP-1 were identified on the northern and eastern walls of the cave.

-Unit III. Formed by the archeological layers IIIn, IIIg, and IIIc, this is the most complex unit documented. It is unevenly distributed throughout the excavation hall because of erosive processes affecting the upper sub-units. At its top, it contains the archaeological layer IIIn, a breccia comprising calcarenite slabs infilled by yellowish calcareous sands and charcoals. It is truncated in the south and only preserved in a small sector at the northern end of the excavation hall, close to the cave walls. It represents the sedimentary infilling of a scoured surface with cryptokarstic structures (Fig I in S1 Supporting Information). The excavated area is less than 2 m^2^. Layer IIIg is composed of weakly cemented and massive yellow calcareous sands and is only preserved close to the cave walls. Archeological remains are almost absent. Layer IIIg presents a scoured surface in the middle of the excavation hall, most likely related to the truncation of layer IIIn. Layer IIIc is the first continuous archeological layer within the Pleistocene sequence. It is a tabular stratum 0.3–0.4 m thick, comprising massive lithochromic calcareous sands of a very pale brown (10 YR 7/3) color with a few calcarenite slabs of cobble and boulder size. Layer IIIc contains abundant charcoal, combustion structures and reddened stones (Figs M to P in S1 Supporting Information).

-Travertine Platform 2 (TP-2). This is a poorly bedded breccia of calcarenite boulders infilled with yellow (10YR 8/1), weathered calcite precipitates, and has a 0.5 m-thick dome shape. TP-2 is stratified between Unit III and Unit IV, extending across almost half the excavation hall (lines B, C and D). Layer IIIn rests on TP-2 in squares B-C/6-7. Layers IIIg and IIIc have a lateral contact with TP-2, indicating that it had already formed at the time Unit III was laid down. Unit IV underlies TP-2 across its entire extent.

-Unit IV. Stratified breccia of boulder-sized travertine (calc-sinter) slabs, with massive calcareous muddy sands of reddish-yellow color (7.5 YR 6/8), partially or totally infilling between the boulders, with a thickness of 0.6 m. Two continuous tabular strata of travertine boulders form natural pavements separating sublayers IV, IV1, and IV2. Unit IV is homogeneously distributed across the entire excavated area. It appears directly below layer IIIc in the central and southern areas of the excavation. In the north it is well stratified below layers IIIn, IIIc, and TP-2.

-Unit V. This is the basal unit of the excavation and is characterized by a breccia made up of yellow (10 YR7/8) cobbles and boulders of travertine (calc-sinter) slabs, infilled with reddish-yellow (7.5 YR 6/8) calcareous muddy sands. Unit V contains cryptokarstic structures. The breccia overlies marls and calcilutite deposits with black cryptocrystalline impregnations (Fig AD in S1 Supporting Information).

Chemical precipitation deposits from Unit V, and stratified breccias from Units V and IV suggest the opening of the lower cave entrance. In Unit IV, the breccias were deposited and human occupations occurred during the latest episodes of collapse of the calc-sinter travertine infill and the Miocene calcarenite forming the roof and walls of the cavity. The boulder and cobble breccia deposits of Unit IV record root-cast sedimentary features, suggesting biochemical weathering (Fig 6) of the calc-sinter travertines and Miocene calcarenite. The color of the matrix infill points to an eolian provenance, suggested by the lithochromic dissimilarity between the reddish yellow color of the muddy sand matrix and the yellow color of the basal calc-sinter travertine with cobbles and boulder slabs.

The sedimentary processes described for TP-2 and Unit III suggest weathering and erosion of the deposits from Units V and IV. Weathering of the cave wall and breccia erosion (TP-2) processes are likely recorded in the color and massive structure, suggesting a stratigraphic gap (paraconformity) and burial of layer IIIc. Erosive episodes can be deduced from scour surfaces and cryptokarstic hollows infilled by the colluvial breccias of layers IIIn and IIIIg. This latter layer presents the smallest calcarenite cobbles and boulder slabs. The erosive and depositional processes described for the top of Unit IV to the top of Unit III probably agree chronologically with the opening of the upper entrance of the cave.

### Occupational dynamics

**Fig 7:**
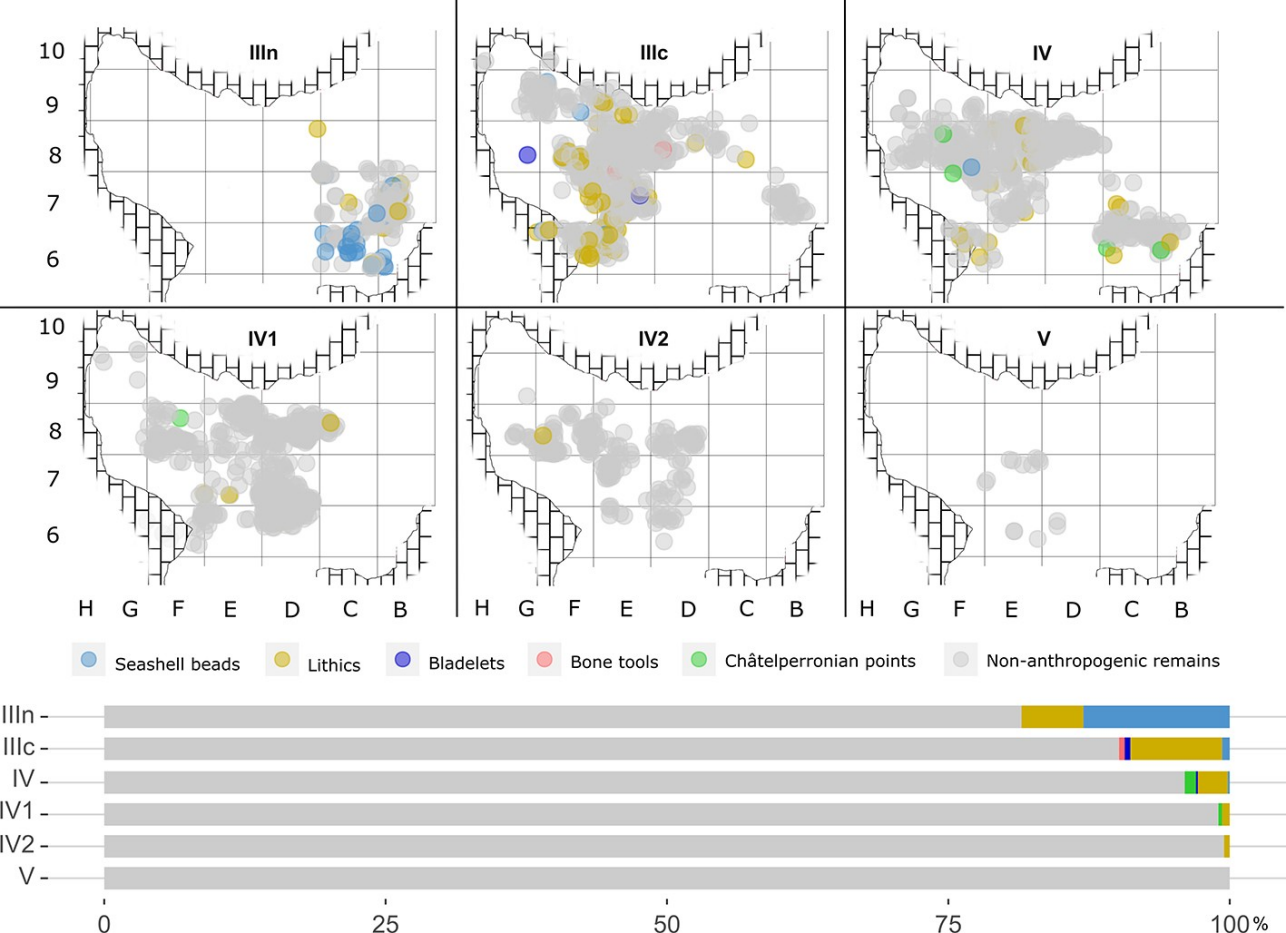
Plot of the findings recorded per layer. Above, XY plot of the 3D-registered remains from each layer. Below, stacked percentage bar plot showing the proportions of non-anthropogenic (unmodified or carnivore modified bones) versus the various anthropogenic remains per layer.

**Fig 8:**
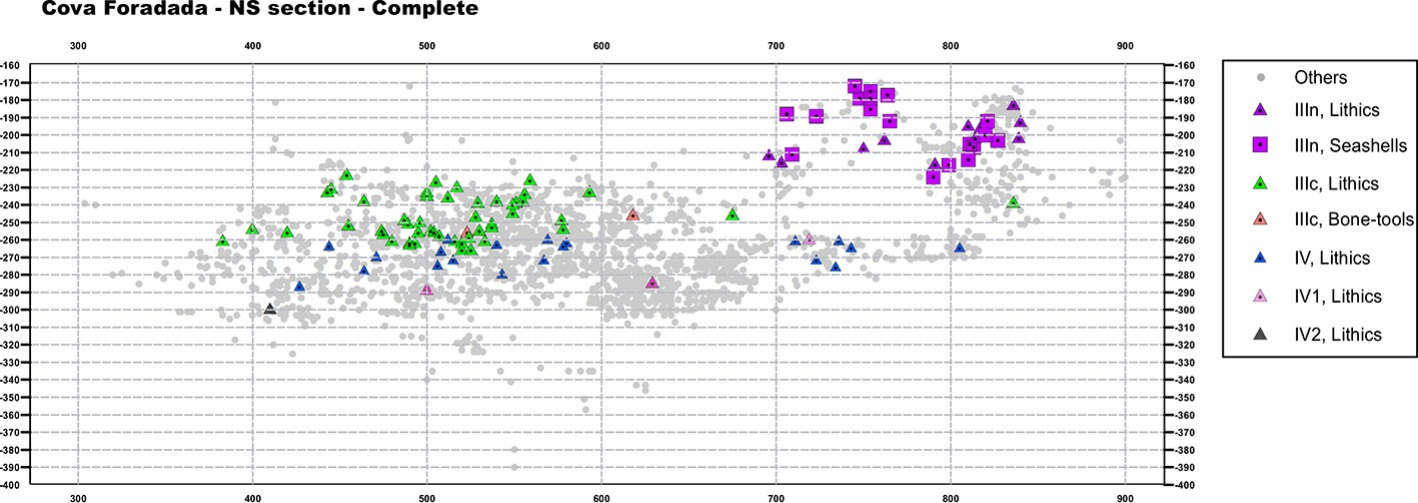
Complete X-Z projection of the archaeological materials. NS plotting of all the 3D-registered archaeological materials at the site. In grey, non-anthropogenic remains. In blue, green, and black, anthropogenic remains from each archeological layer. Partial overlap derives from the EW slope of the Unit III and IV layers.

**Fig 9:**
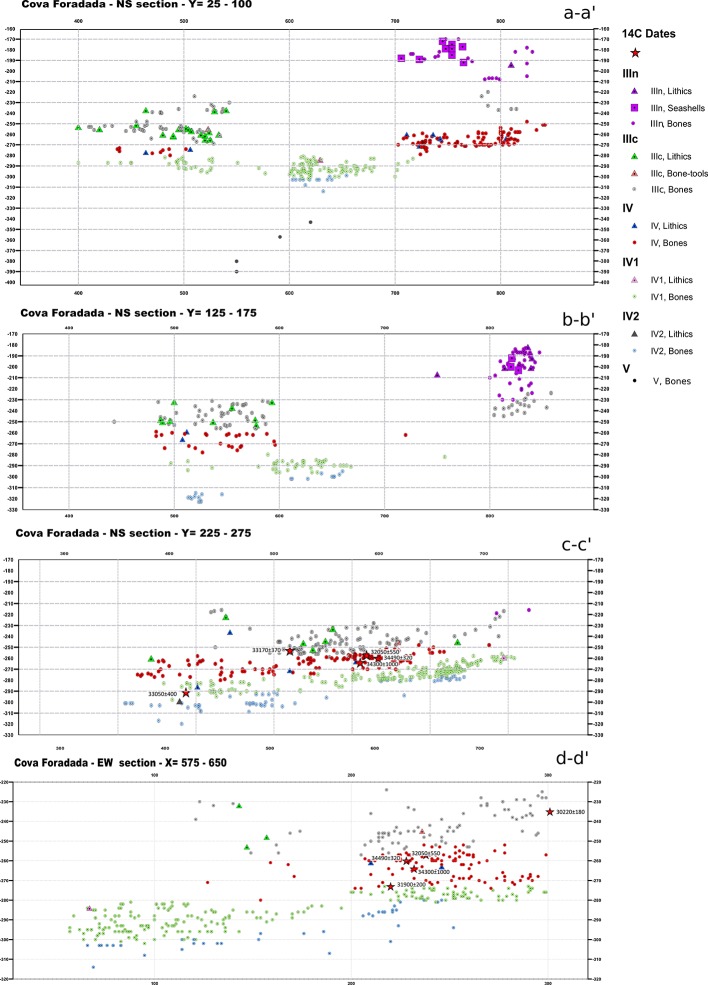
Partial X-Z and Y-Z projections of the archaeological materials. NS (a-a’, b-b’, c-c’) and EW (d-d’) projections of the 3D-registered archaeological materials. Different symbols are used for anthropogenic (lithics and seashells), non-anthropogenic or undetermined (bones) remains, and 14C dates. Depending on the slope of the layers and the representativeness of the section, wider or narrower sections have been plotted. a-a’ and d-d’ = 75cm; b-b’ and c-c’ = 50cm. The position of each section in the cave is shown in Fig 4.

Based on the quantitative and qualitative characteristics of the archaeological assemblages, the Pleistocene hominin occupation at Cova Foradada can be ascribed to a low intensity pattern (Figs 7, 8 and 9).

The by-products of anthropogenic activity, both technological or economic remains and archeological features, are scarce throughout the entire Pleistocene sequence (Table 1); the most solid evidence of a hominin presence at Cova Foradada is provided by the lithic assemblages recovered from the various layers (Table 2). A common pattern in the sequence is the almost-exclusive use of chert as a lithic raw material. Ten petrographic varieties have been identified, mainly associated to Triassic, Lutetian, and Bartonian formations located within the southern spurs of the Catalan Prelittoral Range and the Ebro basin. The Triassic silicifications formed in limestones and dolomites from marine shallow platforms and tidal flats, relate to Tethyan transgressive episodes [78,79]. Paleogene silicifications are replacement products in lutite, marl, gypsum, limestone, and conglomerate facies from lacustrine evaporitic and fluvial formations [80–82]. Systematic geoarchaeological prospections have identified silicifications that are macroscopically and petrographically concordant with those of the Cova Foradada assemblage, in the Montsant, Francoli, Gaia, Foix, and Anoia basins, both in primary positions and within their dispersive systems. The geo-spatial distribution of these source areas ranges from 5 to 60 km from the cavity, suggesting a local-regional procurement range in every layer. Structurally, the lithic assemblages recovered are characterized by fragmented reduction sequences putatively related to short-term occupation dynamics. Formal cores are totally absent, and the only evidence of *in situ* lithic production derives from a few informal and opportunistic bipolar-on-anvil cores from Unit IV. Similarly, the absence of knapping debris suggests that core reduction and tool shaping was rarely if ever undertaken at the site. Retouched tools are the most significant component in the lithic assemblages of each layer, and blades and bladelets represent very high percentages (Table 2). Pointed tools and other alleged hunting-related implements, such as microliths, dominate, including fragmented antler points in the case of layer IIIc. These elements are rarely complete, presenting different kinds of fractures, mostly transversal, from the time of discarding. In addition, a preliminary high-power analysis of the use-wear patterns indicates that between 60% and 70% of the lithics analyzed display use-related micro polishes (S1 Table and S2 Fig).

**Table 1:** Absolute occurrence of the recovered materials per layer. Archeological distribution of the archeological materials from Cova Foradada across the different layers. Lithics, bone tools, seashells, cut-marked bones, and burned bones represent anthropogenic impact on the assemblage formation.

Layer	Lithics	Bone Tools	Seashells	Bones (NSP)	Cut-marked Bones	Burned Bones	Tooth-marked Bones
**IIIn**	39	-	186	160	-	23	1
**IIIc**	75	4	-	934	1	41	20
**IV**	25	-	1	470	1	23	37
**IV1**	6	-	-	528	1	2	12
**IV2**	3	-	-	190	-	6	12
**V**	-	-	-	14	-	0	0
***∑***	148	4	188	2296	3	95	82

**Table 2:** Structural composition of the lithic assemblages. Lithic remains recovered from the Cova Foradada archaeological layers tabulated according to their category. Unret = unretouched, Ret = retouched.

**Layer**	Cortical Flakes			Flakes			Flake Fragments			Blades			Blade Fragments			Bladelet			Bladelet Fragments			Fragments			On-anvil Cores	Debris	∑
	*Unret*	*Ret*	*∑*	*Unret*	*Ret*	*∑*	*Unret*	*Ret*	*∑*	*Unret*	*Ret*	*∑*	*Unret*	*Ret*	*∑*	*Unret*	*Ret*	*∑*	*Unret*	*Ret*	*∑*	*Unret*	*Ret*	*∑*			
**IIIn**	1		1	10	2	12	3		3	2		2	2	3	5	3	1	4	1	3	4					8	**39**
**IIIc**	6		6	22	1	23	9	1	10		1	1	3		3	4	11	15	5		5	1	1	2		10	**75**
**IV**		2	2	4	1	5	2		2	2	3	5		4	4				2		2	2		2		3	**25**
**IV1**				2	1	3					1	1														2	**6**
**IV2**				1		1																1		1	1		**3**
**∑**																											**148**

### Layer IIIn

Layer IIIn was preserved only in the northeastern corner of the excavation hall, in squares B-C/6-7 and partially in D8 (see Fig 7). To the south, in squares D6-D7, it was destroyed by a test pit from an illegal excavation performed prior to 1997. In the central and southern area of the excavation hall, it was severely affected by erosion, and probably also by the sedimentary mixing that occurred during the Holocene funerary use of the cave. Notwithstanding, lithic tools and ornaments typologically attributable to the Gravettian were recovered from both the illegal test pit infill and mixed within the Holocene layers. The archaeological evidence presented here comes exclusively from the excavation of the small intact area.

**Fig 10:**
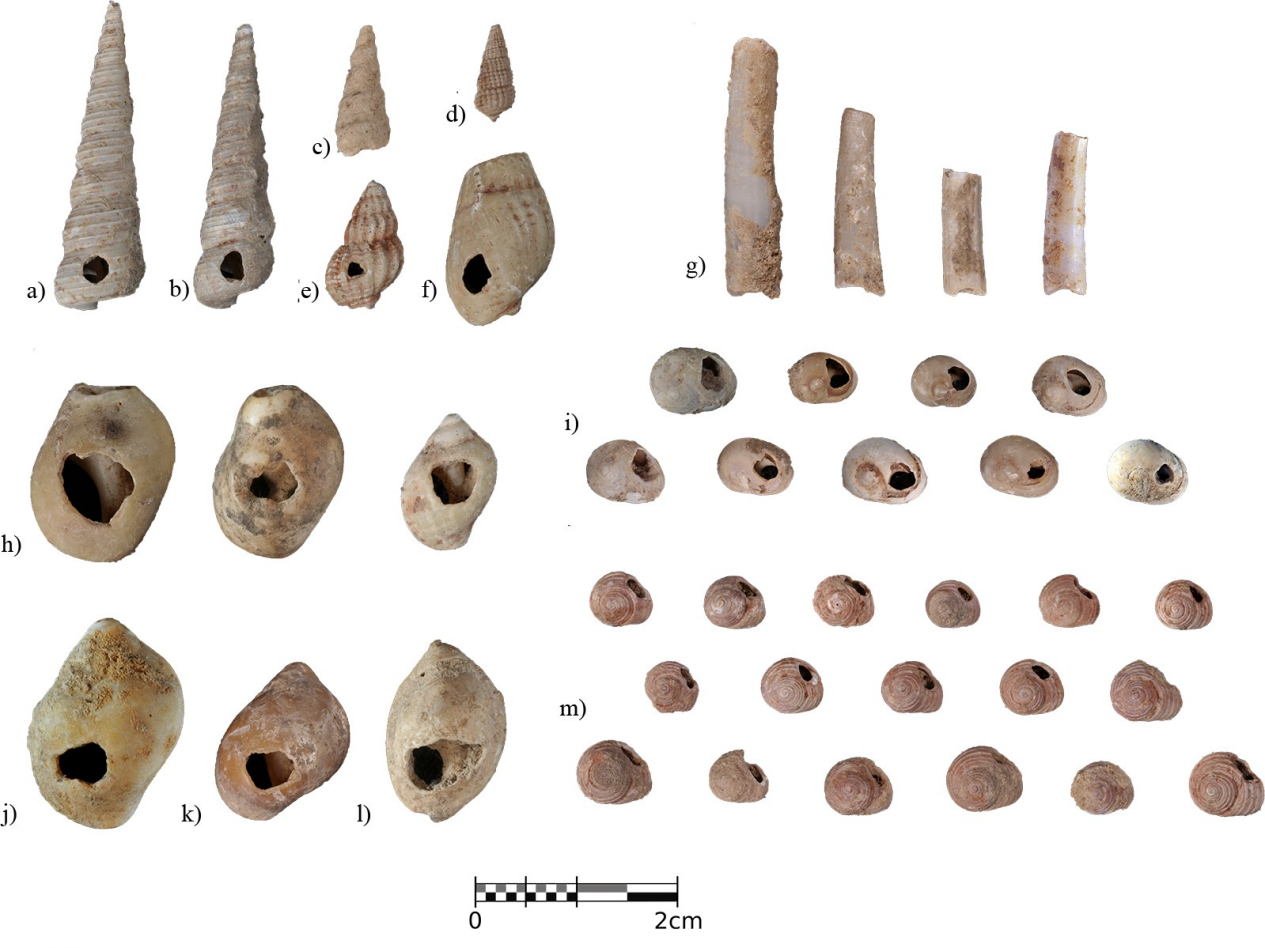
Malacological record from layer IIIn. a-c), *Turritella communis*. d), *Bittium* sp. e), *Tritia incrassata*. f), *Tritia reticulata*. g), *Antalis* sp. h), *Nucella lapillus*. i), *Tritia neritea*. j-k), *Euspira catena*. l), *Nassarius circumcinctus*. m), *Homalopoma sanguineum*.

The most remarkable feature of layer IIIn is the enormous abundance of pierced shell ornaments from a variety of marine gastropod and scaphopod species, primarily concentrated in the eastern part of square B6 (Fig 10). Nine different taxa have been documented including *Homalopoma sanguineum*, *Tritia neritea*, *Tritia incrassata*, *Tritia reticulata*, *Nucella lapillus*, *Turritella communis*, *Bittium reticulatum*, *Nassarius circumcinctus*, and *Antalis* sp. The assemblage is clearly dominated by globular gastropods, particularly *H*. *sanguineum* (n = 101) and *T*. *neritea* (n = 31), and 90% of the specimens present ochre residues in the form of complete coatings as well as isolated spots. All the species documented are Mediterranean-related, with the exception of *N*. *lapillus*, for which an Atlantic origin can be suggested [72,83].

**Fig 11:**
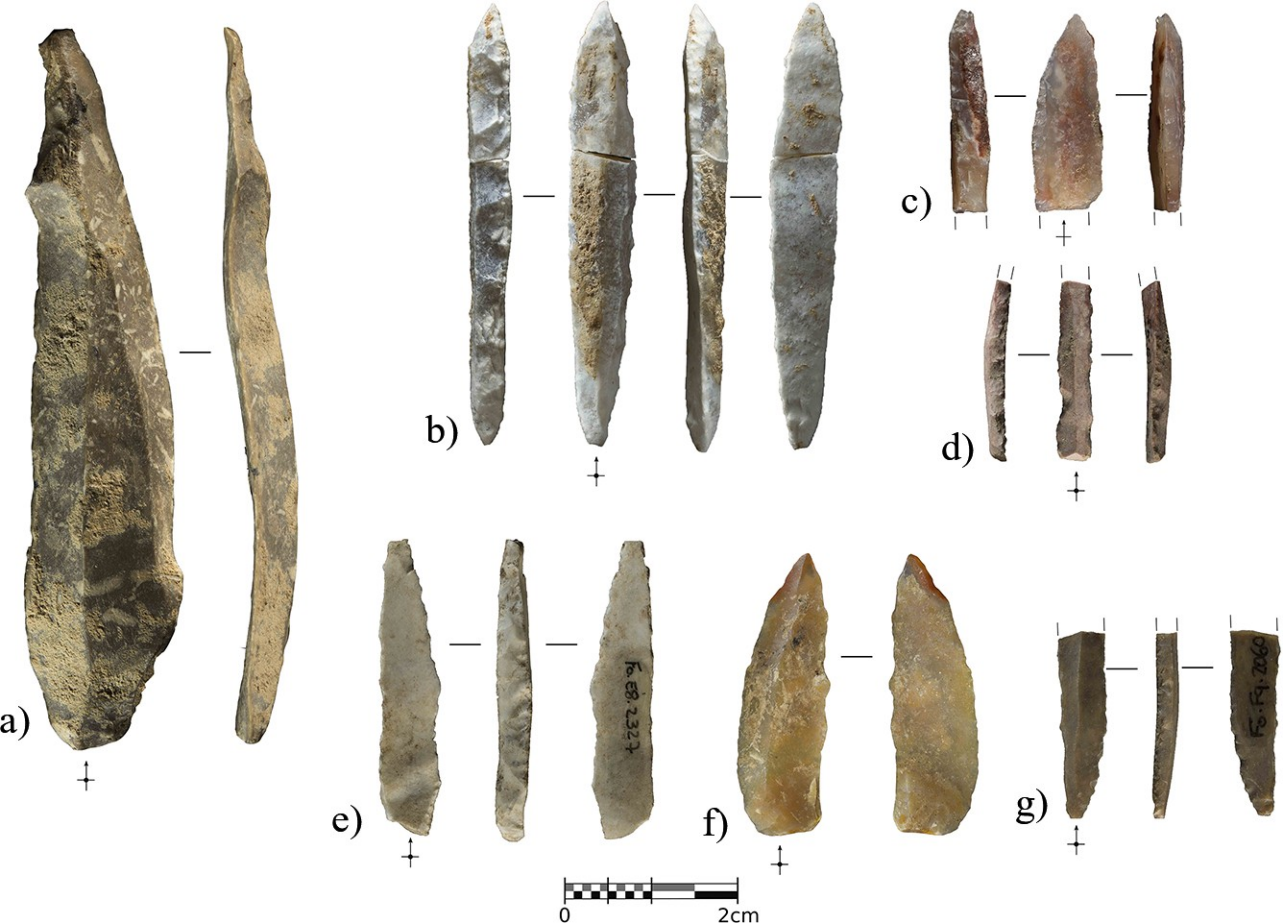
Lithics recovered from layer IIIn. a), unretouched blade with distal ridge-rectification retouching. b) and c), conjoined and distal fragment of Gravettian points with abrupt backing on one edge and direct invasive retouching on the opposite edge. d), fragmented double-backed bladelet. e), small Gravettian point with bipolar backing retouching and direct retouching shaping the proximal part of the right edge. f), inversely retouched borer. g), proximal fragment of backed bladelet with inverse flat retouching on the non-backed edge.

The stone-tool assemblage is composed of 39 pieces including 9 retouched tools (Fig 11). There is a strong laminar component, characterized by laminar blanks and retouched blades and bladelets. When modified, the bladelets always display a direct and abrupt retouch on one of the edges, configuring backed bladelets and/or microgravettes. In the case of the larger blades, they are bilaterally retouched to shape Gravettian points through abrupt direct retouching on one edge and a more invasive simple retouch on the opposite edge to make the blade pointed or regularize the edge delineation. Despite the sample size, both blades and bladelets seem to have been detached from prismatic unipolar cores, while bidirectional reduction strategies are not evidenced. Due to the assemblage size, it is not possible to elucidate much information regarding flaking strategies, in addition to the fact that only one core rejuvenation flake, a semi-crest from a bladelet reduction sequence, and some evidence of ridge rectification within laminar exploitation have been observed. The platforms of all the blades and proximal blade fragments are flat, but there is evidence of strong cornice abrasion in all cases. The laminar products are mostly straight or slightly bent, also presenting convergent edges and triangular cross sections.

### Layer IIIc

**Fig 12:**
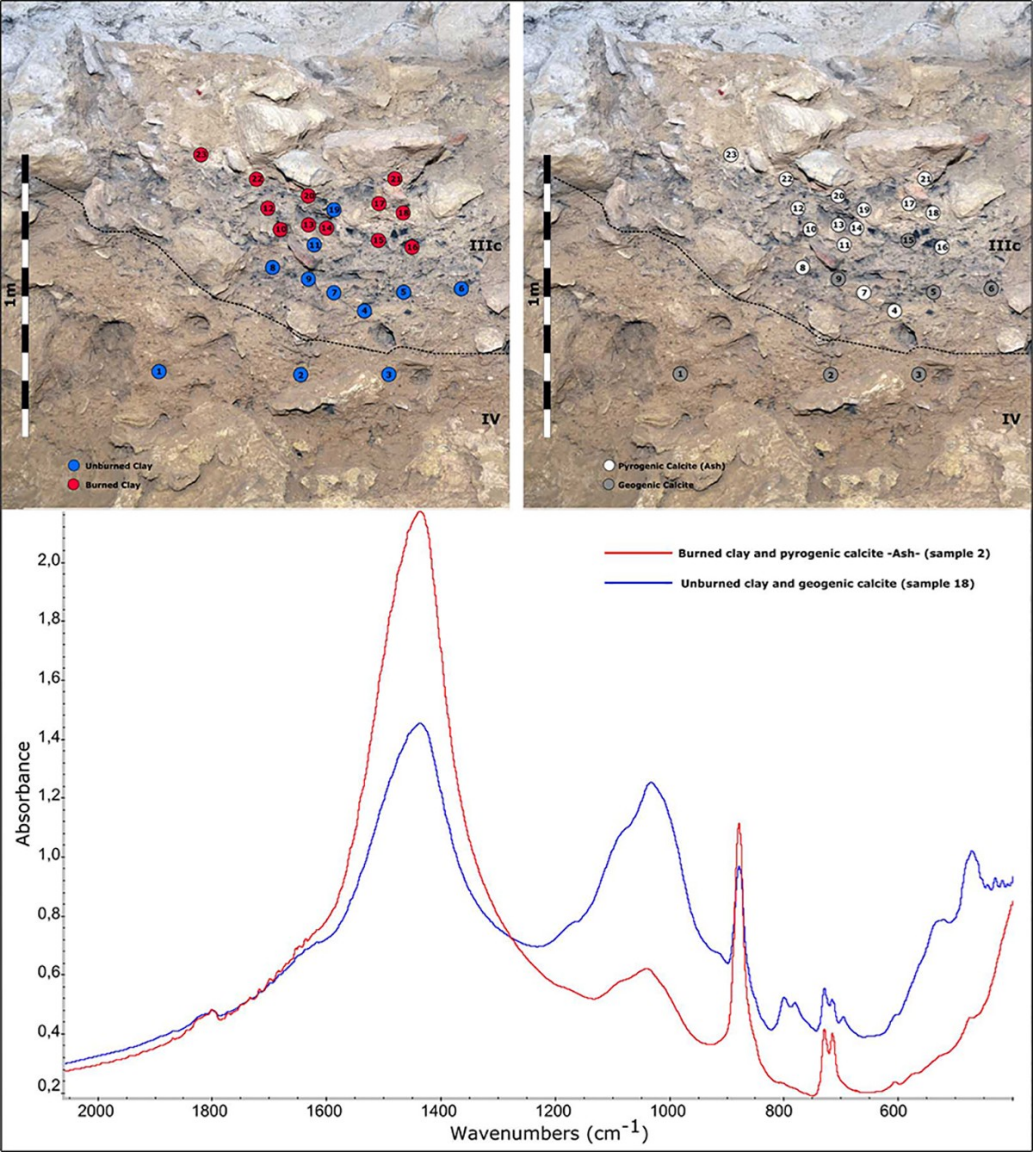
FTIR sampling and spectra. Above, distribution of the FTIR results from layers IIIc and IV (control) samples. Left, distribution of the signals indicating thermally altered clay (red) and non-thermally altered clay (blue). Right, distribution of the results on calcite origin: ash (white) or geogenic (gray). Below, examples of two different FTIR spectra indicating anthropic impact (red) and natural sediments (blue).

Layer IIIc represents the Early Aurignacian occupation. It is characterized by repeated short occupations leaving behind a diagnostic assemblage of stone-tools, mainly bladelets, and some bone and antler artifacts. This occupation pattern is represented in the main stratigraphic section by the interstratification of thin burned horizons, including charcoal and fire-altered sediment and stones, with unburned ones (Fig 12). These fire-related features were only partially identified during the excavation of squares D8 and E8, because their stratigraphic position in these locations coincided with the base of the erosion (Fig G in S1 Supporting Information). However, they were fully preserved in squares E9 and D9, where the reference section for future samplings was maintained. Despite some of the reddish or fire-altered sediment lines having lateral continuity and being clearly visible to the naked eye, we decided to perform Fourier-transform infrared spectroscopy (FTIR) to characterize them. In this way, we avoided misinterpreting charcoal lines generated by other agents as being anthropic-related (i.e., water), or aprioristically attributing the reddish sediments to impact from fire.

For this reason, 20 samples were taken from the main section in IIIc, in addition to others, exclusively for control purposes, from different locations within the layer. In the main section three samples were taken from layer IV, also for control and comparison. The distribution of the FTIR results displays the existence of a dual dynamic of altered and unaltered (samples 19 and 11 in Fig 12) sediments. The observed features are formed by combustion residues (ash and charcoal) and thermal impact (mineral alterations and changes in the color of the sediments), confirming the presence of small and repeated *in situ* fires in layer IIIc. Most of the samples from the upper part of layer IIIc evidenced the presence of fire-altered clay (above 400°C) and pyrogenic calcite, supporting evidence of *in situ* combustion. It is also worth noting that in some parts of the section the combustion structures have been partially dismantled by post-depositional taphonomic processes, leaving only micro-archeological fingerprints. Samples collected from layer IV and the lowest part of layer IIIc presented unaltered clays, and, only in some cases was calcite from pyrogenic origin documented. In this context, the decay of more evident combustion structures can be partially explained by taphonomic processes, but it is also very likely that the combustion record deriving from short-term and low-intensity occupations was characterized by the presence of very little waste and a low thermal impact on the sediments [84]. It is, therefore, important to note that only a small proportion of the faunal remains recovered from layer IIIc (4.4%) show signs of thermal alteration (Table 1).

**Fig 13:**
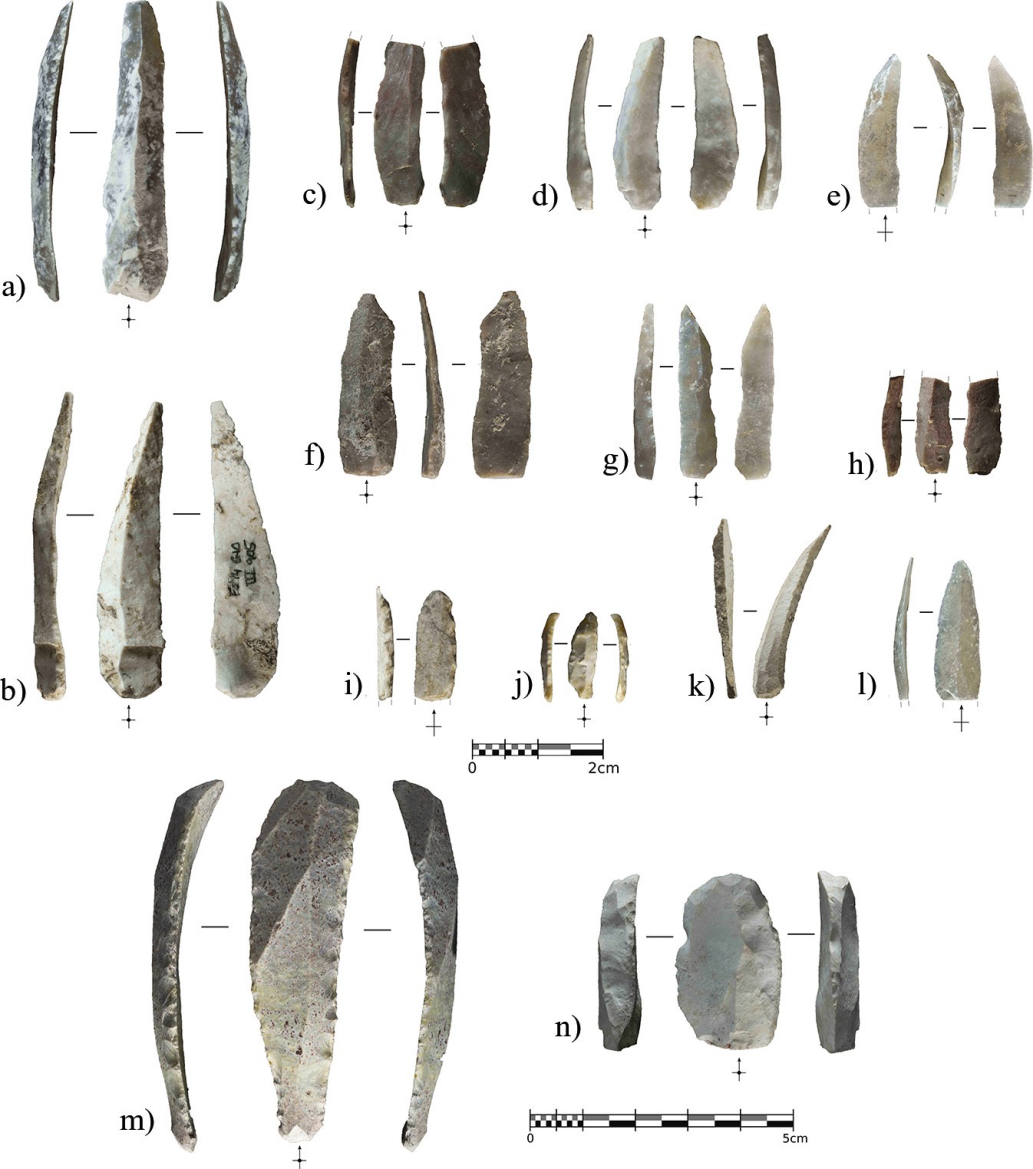
Lithics recovered from layer IIIc. a) and b), bladelets with partial marginal and direct semi-abrupt retouching. c-j), Retouched bladelets (c and h) with marginal inverse semi-abrupt retouching on the right edge; d-g) and i-j) with different distributions of marginal direct and semi-abrupt retouching). k and l), unretouched bladelets. m and n), end-scrapers.

The lithic assemblage from layer IIIc mainly comprises cortical flakes, blades, and bladelets (Table 2 and Fig 13). The blades were detached from prismatic cores and used as blanks for the configuration of domestic tools, namely end-scrapers and side-scrapers. Notwithstanding, the most abundant sub-assemblage is formed of retouched and unretouched bladelets. Despite the absence of cores, most of the bladelets display characteristics related to the exploitation of carinated or end-scraper-like cores. The size of the complete bladelets ranges from 13.5 to 33.4 mm in length (mean = 23.49 mm), and from 5 to 12.3 mm in width. Any statistical approach to the assemblage must be considered cautiously given the small sample size; however, as an exploratory analysis, the average length differs when comparing retouched and unretouched bladelets (26.8 mm vs. 21.08 mm), although there are no statistically significant differences according to either a T-test analysis of the means (p-value = 0.344) or the Kolmogorov-Smirnov distribution test (p-value = 0.958). These results indicate that size was not determinant in the selection of blanks for retouching. In addition, two retouched elements classified as blades are in the typometric boundary between blades and bladelets (44–45 mm in length), and, according to their dimensions and rectilinear ventral delineation, could have been detached from prismatic cores.

Morphologically, bladelets are mostly straight or deviate to the right in the horizontal plane, with parallel edges slightly dominating over convergent ones in the same way that trapezoidal cross-sections are more typical than triangular ones. The ventral delineation is curved in eight cases, curved and slightly twisted in seven, and straight or convex in two. The scar distribution shows unipolar longitudinal exploitation with distally convergent negatives. Of the 21 bladelets recovered, ten have modified edge/s, forming a highly heterogeneous group where unilateral, direct, and semi-abrupt marginal retouching dominates. In these cases, retouching is continuous along the entire edge, apart from the tip, or discontinuous, affecting only the proximal and distal parts. In two cases it is inverse, semi-abrupt, marginal, and located on the right edge, configuring Dufour subtype Dufour bladelets. In the two cases of bilateral modification, the retouching is direct and semi-abrupt, but never convergent (S3 Fig).

**Fig 14:**
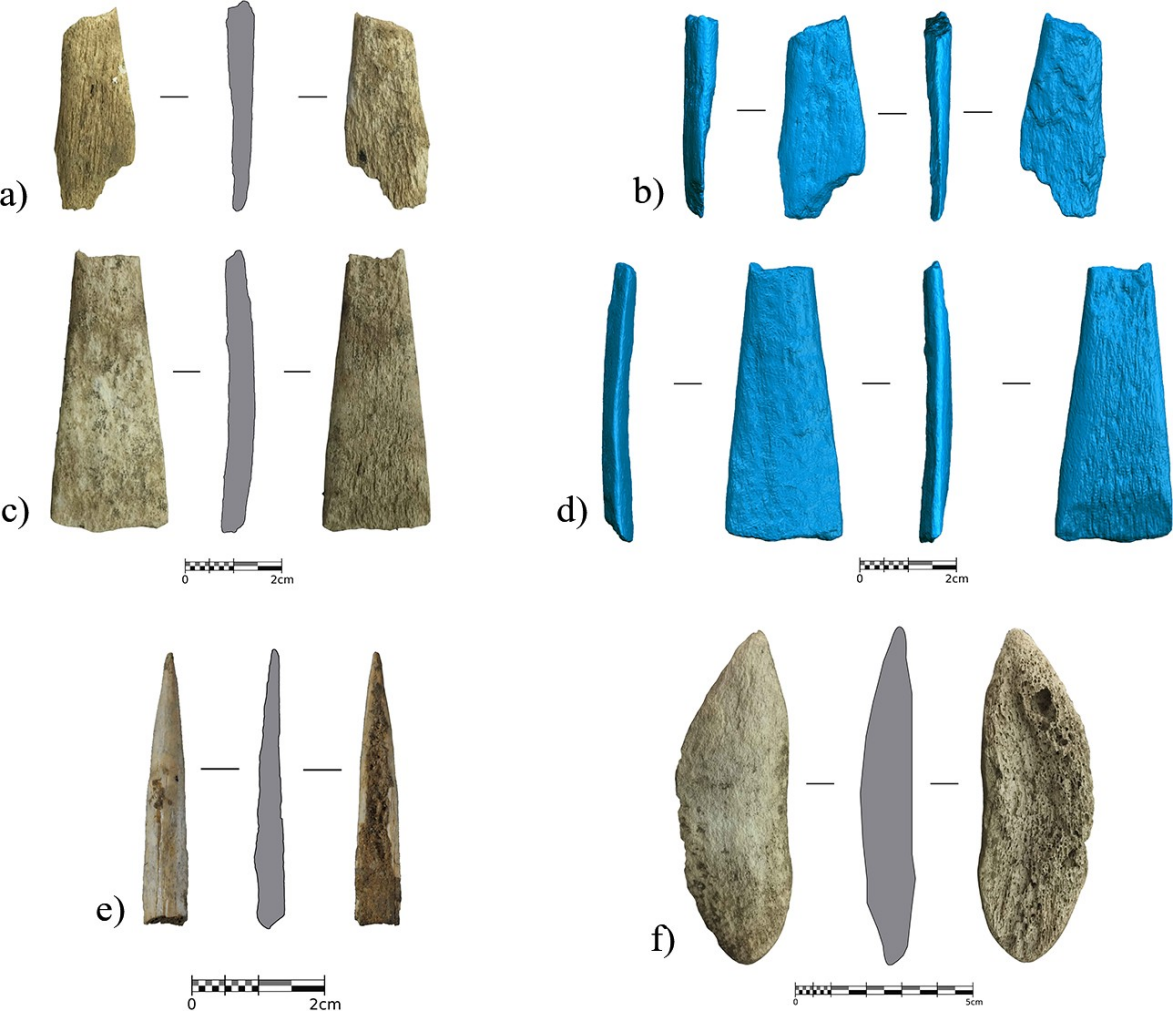
Antler and bone tools from layer IIIc. Antler tools from layer IIIc. a-d) fragments of split-based points; a and c photographs of the tools; b and d) untextured views extracted from the 3D models where the fracture surfaces can be much more clearly observed. e), distal fragment of a bone awl. f), antler blank.

A significant and diagnostic aspect of layer IIIc is the presence of a small osseous assemblage (Fig 14) comprising one bone awl (Fig 14E) and two antler point fragments which are interpreted as broken split-based points (SBPs) (Fig 14A, 14B, 14C and 14D), as well as one antler blank, likely linked to the production of organic projectile points (Fig 14F). The two antler points correspond to the medial-proximal part, displaying a loss of one of the lips probably because of functional breakage, as evidenced by a tongue-type fracture generated during an impact [85,86]. Several criteria, such as the raw material; the extent of the work on the surface of the piece; the cross-section; the identification of the maintenance and hafting system have been taken into consideration in order to classify the Foradada antler tools as SBPs. Like most organic projectiles of the Aurignacian [87–91], these elements are made of antler that probably came from a red deer (*Cervus elaphus*), a species represented at the site, and for which the average osseous cortical tissue matches well with the Foradada elements (~5/6 mm).

The antler points were wholly worked by scraping, having the elliptical cross-section most common in European Aurignacian SBPs, and derived from their production through longitudinal splitting [92]. The edges of these blanks were then regularized by scraping, resulting in an elliptical cross-section. The tools measure 51 and 37 mm in length. We can thus assume, that, after being broken, they were discarded on-site due to the impossibility of their being reappointed because of both their small size and the proximal fractures, which would have hindered hafting.

The complete blank is slightly bigger (87x27x10 mm), but highly compatible morphometrically with the two points from the site, being. As a result of the longitudinal splitting of the antler beams, it has a sub-rectangular morphology (modified by scraping at its distal and proximal ends, where shaping of the point and the base of the projectile was initiated), straight lateral fracture planes, and a proximal tongue fracture plane resulting from the final extraction by flexion. Considering its width and thickness, the most constant values of the SBP [91], in addition to the loss of material during the final shaping of points, it seems that the blank and the points are part of the same antler exploitation sequence. This does not necessarily mean that the points and the blank were produced at the site, but it can be assumed that they are part of the technical behavior of the groups exploiting this territory.

### Layer IV

Lithostratigraphic Unit IV, and sublayers IV, IV1, and IV2, represent a depositional interval when the cave, according to the archaeological assemblage, was even less frequented by human groups than layers IIIn and IIIc. The anthropogenic remains recovered include 34 lithics, a cut-marked raptor phalange, and a seashell. The uppermost sublayer, IV, was the richest in remains, yielding 25 of the 34 lithics, while six were recovered from sublayer IV1 and three from sublayer IV2. Most of the lithics from sublayer IV were recovered in the northern area of the excavation, which was sealed under TP-2. This area is currently under excavation, so it is possible that further artifacts will be recovered from IV1 and IV2. Here we present the main characteristics of the lithic assemblage, integrating the materials from the three sublayers.

The 34 lithics include 25 complete or fragmented flaking products. Three elements bear opposed percussion marks, one of them being an angular fragment from a core on-anvil, while the other two are splintered pieces, probably used as wedges [93] or as small cores on-anvil [94].

**Fig 15:**
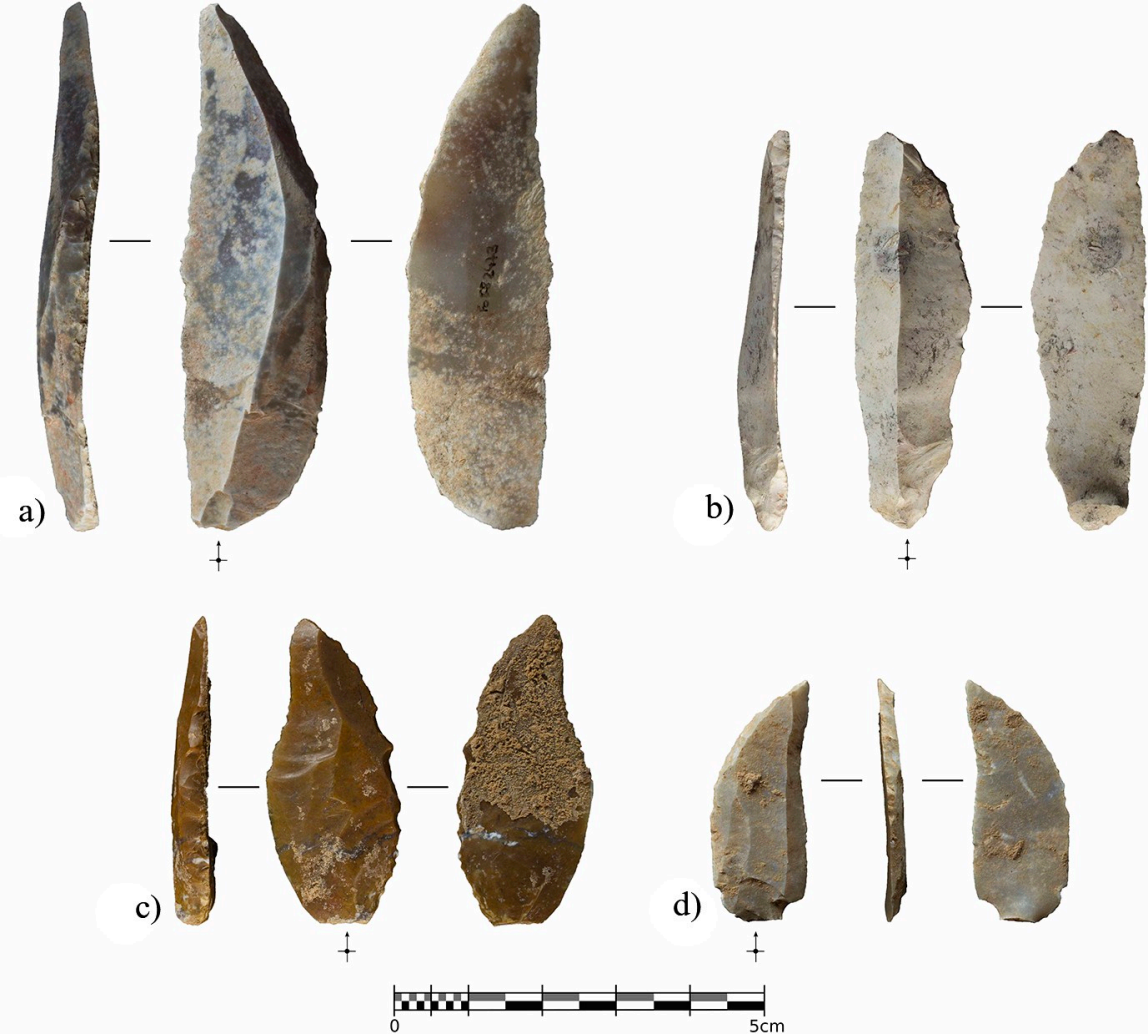
Complete backed points from Unit IV. Retouched blades from Cova Foradada Unit IV. a and b) show discontinuous retouching along the edge. c), a fully retouched edge. d), a mid-distal modification of the blade.

**Fig 16:**
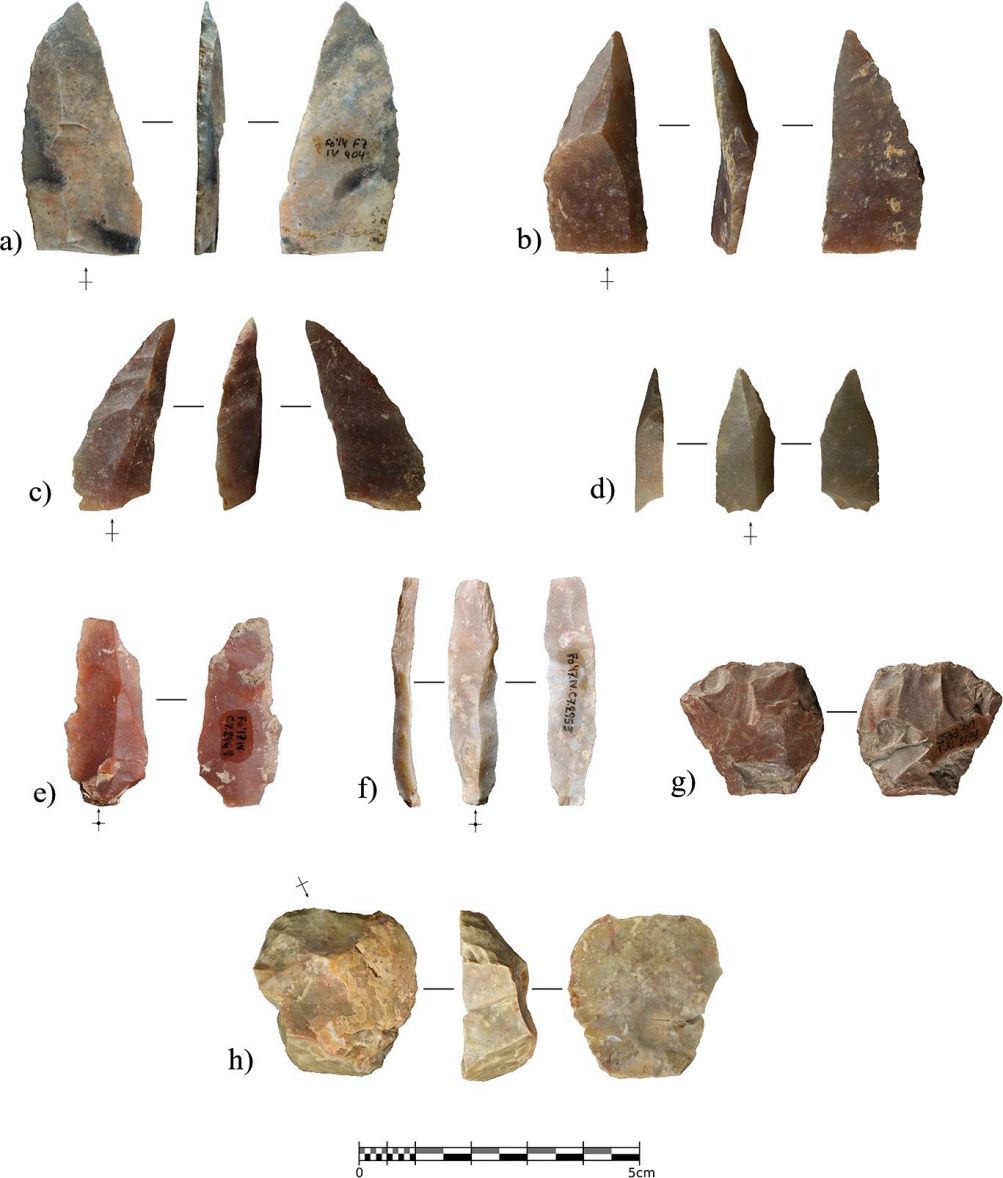
Retouched assemblage from Unit IV. a-d), distal fragments of Châtelperronian points. e), unretouched blade. f), partial *néocrête*. g), splintered tool. h), end-scraper.

However, the most significant pattern in the assemblage involves the abundance of retouched tools in relation to the total number of remains. Besides the splintered tools, formal retouching is present on nine pieces. Eight of these are complete (n = 4) or fragmented (n = 4) blades with lateral abrupt, or backing, retouching (Fig 15A–15D and Fig 16A–16D). Significant intra-group variability between these retouched blades can be appreciated both in the raw material type and in the retouching patterns. Chert provenance studies are still being developed, however each of the retouched blades is clearly made from a different variety of raw material. This does not necessarily suggest different chert provenance since it can be reflecting the natural variability within the same procurement area, but clearly indicates separated knapping sequences for each of the tools. Complete elements show three different distribution patterns for the abrupt edge retouching, in all cases being direct and unilateral. In one case (Fig 15C), the retouching was continuous from the base to the tip. In the basal and mid parts of the blade, the retouching was invasive and strongly modified the edge, reaching 90% of the blade’s maximum thickness. In contrast, the distal part was only slightly modified through marginal abrupt retouching, reaching 27% of the maximum thickness, aimed exclusively at generating a pointed blade. In two other cases, (Fig 15A and 15B), the retouching was discontinuous along the edge. In the first, the retouching completely modified the proximal part of the blade, including the platform, through marginal backing generating a convex edge morphology. The mid-part of the blade presents more invasive retouching, reaching 75% of the blade’s maximum thickness at this point. In this case, the converging edges of the blade already generated a pointed tool, and this remained unmodified. In the second, marginal retouching affected the proximal part of the edge, partially modifying the platform, while more invasive retouching in the upper-mid and distal parts of the edge, generated a pointed blade. The distal tip presents a small fracture resembling certain impact fractures reported elsewhere [95, 96]. Finally, in another case (Fig 15D), the retouching was continuous from the mid-part of the blade edge to the distal tip, reaching the maximum possible thickness.

Within the fragmented tools, and considering that the proximal parts are missing, there are also fully retouched edges on one side (Fig 16A and 16B), but also marginal distal configurations on the other (Fig 16C and 16D). The former strongly modifies the edge delineation, as seen in Fig 16B. In the two cases showing marginal distal configurations, retouching is restricted to the extreme distal end, exclusively affecting the morphology of the blade tip.

In a strictly typological classification, according to existing taxonomic lists, the retouched blades represent both typical and atypical Châtelperronian points [97]. Even considering the variability showed by the retouch patterns, all the modified blades share a general shaping objective where retouching is devoted to configuring a convex edge and a pointed tip (S4 Fig). The original morphology of the unmodified blade determined the positioning and intensity of the retouching. A convergent blade was modified to generate a convex edge morphology (Fig 15A), while parallel blades were modified to the extent required to create a convex edge as well as a pointed tip (Fig 15B–15D, Fig 16A–16D). This variability in shaping strategies for the Châtelperronian points, combining the configuration of thick and continuous backs with thin and/or discontinuous ones, points to adaptation of the backing retouching according to the morphological features of the blank. Typometrically, it is noteworthy that two size modes can be inferred from this small sample. The largest points were shaped from long (65.5 and 51.1 mm), wide blades, while the smallest were made from short (38.7 and 31.6 mm), wide blades, or laminar flakes. The average dimensions of the complete elements are 57.6 x 20.7 x 4.9 mm, with an elongation index of 3.48; the dimensions of the fragmented remains (44, 38.1, 34.7 and 23.8 mm) indicate that some of these were probably even longer (Fig 16A–16C). All the points present almost straight, or slightly curved, dorso-ventral profiles; an analysis of the dorsal surface indicated a unipolar knapping modality for producing the blanks, but sometimes scars from an opposite platform are present (Fig 15C and Fig 16B and 16C). These scars (S5 Fig) are shorter, and probably resulted from the rearrangement of the flaking surface convexity from an opposite platform, and not from alternate exploitation.

The other significant lithics recovered from Unit IV are an unmodified blade, presenting a distal fracture and strong edge damage (Fig 16E), a laminar blank, showing distal ridge modifications, a possible partial néocrête (Fig 16F), a splintered piece (Fig 16G) showing a marked double patina as a result of a recycling episode after having been used and abandoned [98, 99], and one thick end-scraper shaped from a cortical flake (Fig 16H).

A cut-marked pedal phalanx of an imperial eagle (*Aquila adalberti*) was recovered from sublayer IV1. The location of the cut marks and a comparison with similar elements from other Middle Paleolithic and Châtelperronian sites [100], relates this element to the possible extraction of the claw for symbolic purposes.

**Fig 17:**
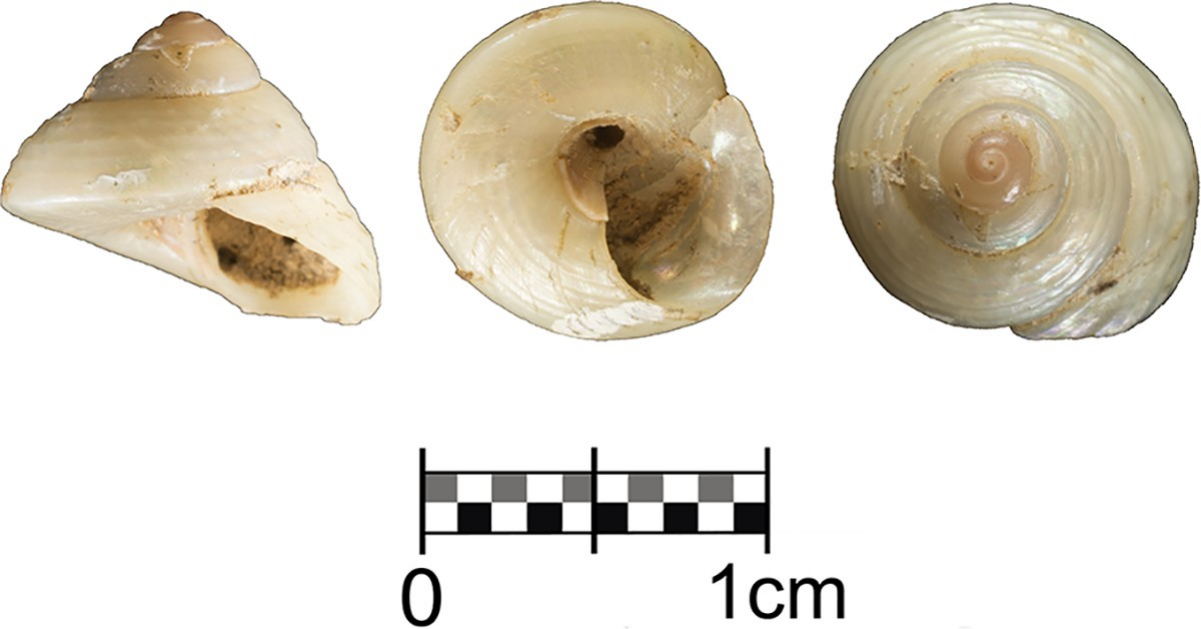
Seashell from Unit IV. Unpierced *Steromphala varia* shell recovered from layer IV (Right).

Layer IV also yielded a *Steromphala varia* gastropod (Fig 17). The shell has lost all its external coloration allowing the underlying pearly surface to stand out. It was recovered below a pile of highly cemented boulders, between these and the cave wall, formed during the deposition of layer IV. This specimen of *Steromphala varia* is unique at the site, as no other examples of this taxon have been observed among the abundant shell assemblage of layer IIIn, and it is also the only unpierced seashell found at the site.

### Zooarchaeological and taphonomic analysis

A total of 2296 faunal remains from Cova Foradada were analyzed. These remains were unevenly distributed between the various layers, with layer IIIc yielding the most (n = 934) and layer V the least (n = 14) (Table 3). The taxonomic representation, and the skeletal and mortality profiles are similar in every layer, with small variations observed between them. The presence of abundant species and specimens of carnivores and large raptors, young carnivores, tooth marks, digested bones, and coprolites all together suggest that non-human predators were predominantly responsible for the faunal accumulation. Nevertheless, all the layers, except layer V, show differing degrees of anthropogenic contribution. This provides the opportunity to explore some subsistence aspects of the hominin groups that occupied the cave.

**Table 3:** Faunal assemblage from Cova Foradada. Zooarchaeological and taphonomic accounts of the different layers of Cova Foradada. The columns show the Number of Identified Specimens (NISP) and their frequency. *Percentage calculated according to humerus + tibia + femur NISP.

Taxa	IIIn	%	IIIc	%	IV	%	IV1	%	IV2	%	V	%
Bovinae cf. *Bos*/*Bison*	1	0.8	1	0.1	10	3.1	4	0.8	0	-	0	-
*Equus sp*.	0	0	6	0.7	16	5.0	3	0.6	0	-	0	-
*Cervus elaphus*	1	0.8	32	3.8	18	5.6	12	2.5	2	1.2	0	-
Ungulate	0	0	3	0.4	8	2.5	7	1.4	2	1.2	0	-
*Ursus* cf. *arctos*	0	-	0	-	1	0.3	2	0.4	1	0.6	1	8.3
Canidae cf. *Canis*/*Cuon*	0	-	1	0.1	1	0.3	0	-	0	-	1	8.3
*Vulpes vulpes*	0	-	2	0.2	0	-	0	-	0	-	1	8.3
*Panthera leo*	0	-	2	0.2	0	-	0	-	0	-	0	-
*Panthera pardus*	0	-	6	0.7	1	0.3	0	-	0	-	0	-
*Lynx pardinus*	12	9.6	59	7.1	35	10.8	38	7.8	28	16.8	3	25
*Crocuta crocuta*	0	-	3	0.4	0	-	0	-	0	-	0	-
Carnivore	0	-	1	0.1	5	1.5	4	0.8	1	0.6	0	-
Leporidae	76	60.8	498	59.6	191	59.1	369	76.1	127	76.0	6	50
Large Raptor	2	1.6	3	0.4	7	2.2	3	0.6	2	1.2	0	-
Small Bird	33	26.4	218	26.1	30	9.3	43	8.9	4	2.4	0	-
Testudinae	0	-	1	0.1	0	-	0	-	0	-	0	-
Indeterminate	35	28	98	11.7	147	45.5	43	8.9	23	13.8	2	16.7
Total NISP	125	100	836	100	323	100	485	100	167	100	12	100
Total NSP	160	-	934	-	470	-	528	-	190	-	14	-
Cut Mark	0	-	1	-	1	-	1	-	0	-	0	-
Burned	23	14.4	41	4.4	23	4.9	2	0.4	6	0.5	0	-
Tooth Mark	1	0.6	20	2.1	37	7.8	12	2.2	12	3.2	0	-
Coprolites	0	-	8	-	7	-	0	-	0	-	0	-
**Leporidae Taphonomy**	**IIIn**	**%**	**IIIc**	**%**	**IV**	**%**	**IV1**	**%**	**IV2**	**%**	**V**	**%**
Tooth Marks	1	1.3	1	0.2	10	6.2	9	2.7	6	9.7	0	-
Burned	16	21.1	27	5.4	18	11.1	2	0.6	2	3.2	0	-
Cut Marks	0	-	0	0	1	0.6	0	-	0	-	0	-
Shaft Cylinder	5	6.6	21	4.2	13	8	3	0.9	3	4.8	0	-
% Shaft Cylinders*	-	31.3	-	17.4	-	25	-	3.6	-	7.7	0	-

Mesovertebrate remains (small birds of the families Corvidae, Phasianidae, and other small passerines, and leporids of the genus *Lepus* and *Oryctolagus*) are the most abundant taxonomic group in every layer, representing between 50% and 87% of the remains identified (NISP = 1595). Although preliminary, an analysis of the anatomical representation of this mesofauna suggests that all skeletal parts are represented in the assemblage, including small bones as well as phalanges and isolated teeth, from tens of individuals of all ages. Postcranial elements of leporids are better represented than the cranial parts, and posterior limbs, especially the tibia, are more frequent than anterior limbs. The level of breakage is low in leporids and small birds, but the main long bones of leporids have been strongly fractured by both postdepositional/excavation breakage and green breakage.

The second most important taxonomic group involves carnivorous mammals; these are represented by 209 specimens from seven different species. Some show recurrent cave behavior, such as the Iberian lynx (*Lynx pardinus*), brown bear (*Ursus arctos*), leopard (*Panthera pardus*), and hyena (*Crocuta crocuta*). Others, like the fox (*Vulpes vulpes*), wolf/dhole (*Canis*/*Cuon*), and lion (*Panthera leo*) typically visit caves sporadically. The Iberian lynx is the best-represented carnivore in Cova Foradada (175 specimens), and the only one found in layer IIIn. Most of the carnivores are represented by the isolated remains of single, prime-aged individuals, although for lynxes there is a wider representation of elements from the entire skeleton, belonging to a minimum of 15 individuals (50% of the carnivore MNI) of all ages, including perinatal (Table 3). The integrity of the carnivore elements is high. Usually, these are complete or near complete although some lynx limb bones evidence green and dry fractures. It is also important to note that 15 coprolites have been recovered, eight from layer IIIc and seven from layer IV. The coprolites can be grouped into two morphotypes, those attributable to Hyaenidae and those of small carnivores [101].

The ungulates identified (NISP = 126) are red deer (*Cervus elaphus*), large bovids (*Bos*/*Bison*), and equids (*Equus* sp.). All these taxa are represented by just a few specimens (less than 10% of the NISP in each layer) and individuals (a maximum of three). However, their importance in the assemblage may be greater, since many of the indeterminate remains may correspond to medium-sized and large ungulates. It has been estimated that there are at least 18 individuals of ungulates: five large bovids, five equids, and eight red deer. Younger individuals are dominant (n = 11), and there is only one old-aged deer. The level of bone breakage is high in this taxonomic group and no elements have been recovered fully intact, with the exception of small bones like phalanges and articular bones.

Other taxa are only occasionally represented but have important implications in the interpretation of the Cova Foradada deposits. On one hand, the Testudinae (cf. *Testudo hermanni*) is represented by one isolated specimen in layer IIIc. Its presence must be indicative of a mild climate and the survival of some populations in NE Iberia after their disappearance in the region at the end of Middle Paleolithic times [102]. On the other hand, large raptors are represented by 17 remains corresponding to the golden eagle (*Aquila chrysaetos*), Iberian imperial eagle (*Aquila adalberti*), griffon vulture (*Gyps fulvus*), and red kite (Accipitridae cf. *Milvus milvus*). Most of the bones are complete phalanges of adult individuals. Their taphonomy indicates interactions between raptors and humans in layer IV [100].

Analyses of bone surface modifications indicate the predominant role of the cave’s post-depositional environment, with features ranging from manganese coatings, chemical corrosion, cementation, and others connected to biological activity, such as root etching and microbial bioerosion. Additionally, a small proportion of specimens show modifications relating to carnivores and humans (Table 1). With regard to carnivores, tooth marks in the form of pits and scores (especially in layer IV), furrowing, digested bones, and macromammal limb bone shaft cylinders have been documented. Leporid tooth marks and digested bones are present, but the percentages of occurrence are low, except in layer IV (Table 3).

Finally, some anthropogenic modifications in the form of cut marks, burned bones, and bone breakage have been observed. There are cuts on three bones: one vertebra of a red deer from layer IIIc; one tibia of rabbit from layer IV; and the pedal eagle phalange from layer IV1. Burned bones (n = 95) are found in all the layers except layer V, varying in quantity from 14% in layer IIIn to 0.4% in layer IV (Table 1). Color changes were observed on ungulate and carnivore remains, leporids, and a Testudinae plaque fragment from layer IIIc.

**Fig 18:**
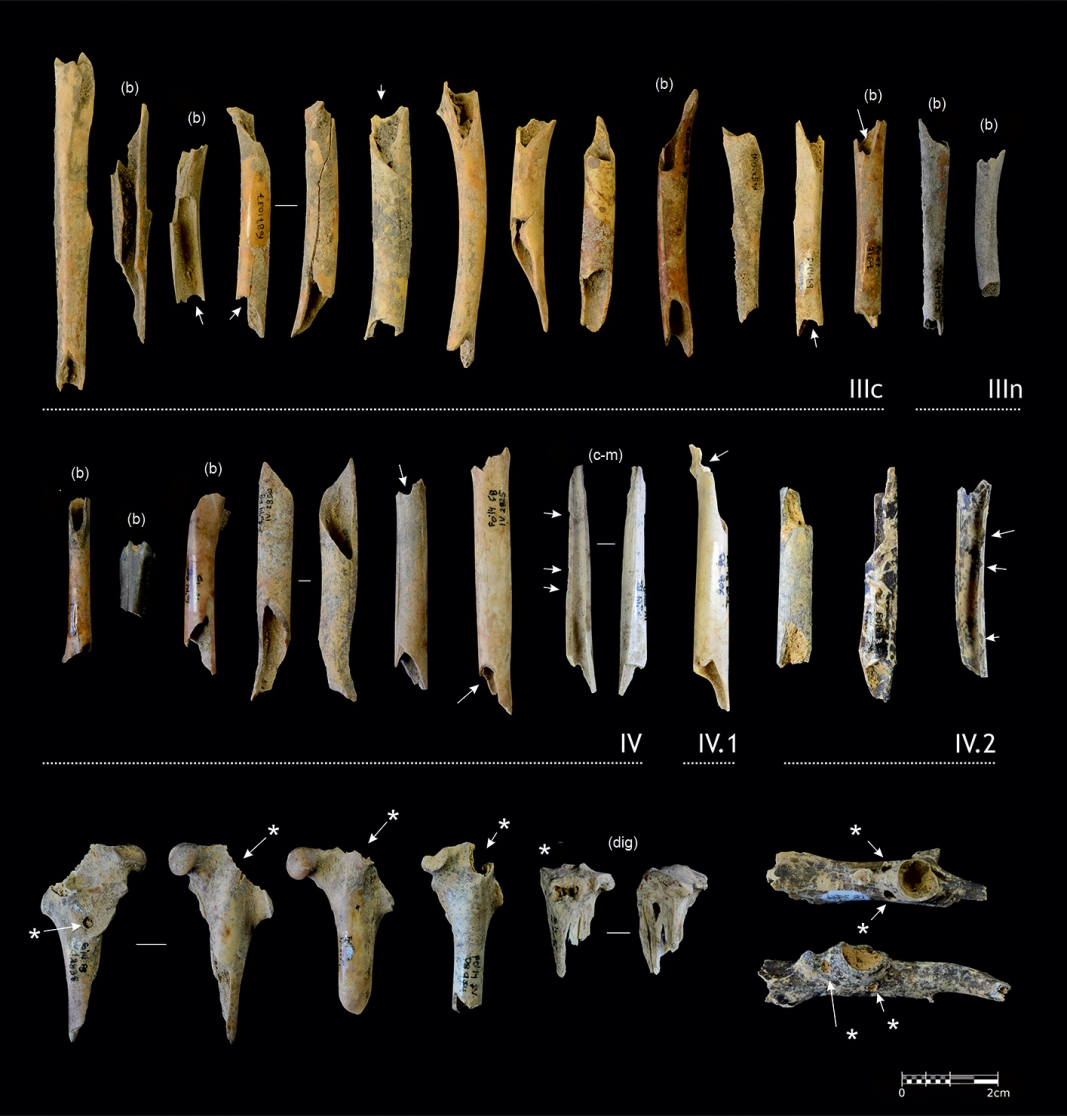
Breakage patterns on rabbit limb bones. Examples of human (midshaft bone cylinders) and carnivore bone modifications on rabbit bones from Cova Foradada. The white arrows point to notches, probably produced by human bites, (b) indicate burned bones, (c-m) indicate cut-marked bones, * indicate carnivore tooth marks, (dig) indicate digested bones.

Bone breakage patterns are exclusively seen on rabbit limbs, in the form of midshaft cylinders (NISP = 45) (Fig 18), and occur in all the layers except layer V. Some large notches on macromammal limb shaft fragments could indicate anthropogenic breakage, but with an absence of percussion marks, and considering the scarcity of cut marks, we must be conservative with this interpretation.

### Carnivore dynamics

The data presented indicates a clear use of the site by different non-human predators throughout the Cova Foradada sequence. On the one hand, the important presence of leporid remains, with high percentages of complete bones, significant levels of long-bone breakage, predominance of the posterior appendicular skeleton over the anterior, predominance of distal appendicular elements over the proximal one, and a low presence of tooth marks, as well as the high numbers and integrity of lynx remains, including immature individuals, indicate that the cave served primarily as a lynx den [103,104]. Large raptors, which in the Iberian Peninsula primarily prey on rabbits, contributed to the accumulation of leporid bones. However, the Iberian lynx is a specialist predator and only occasionally preys on ungulates. The presence of other large bone accumulators, such as hyenas and leopards, and their respective taphonomic signatures in the form of large tooth marks, intense furrowing, large digested bones, and coprolites, also suggest that the cave was a habitual refuge for these taxa. The high numbers of infantile ungulates and an attritional mortality profile throughout the sequence, such as that generated by other predators, reinforces the non-anthropogenic origin of most of the faunal assemblage.

### Anthropogenic activities

Although both the taphonomic results and density of cultural remains indicate scarce human occupation of the cave, almost every layer at Cova Foradada has yielded anthropically modified bones. Most lines of evidence, when considered individually, are ambiguous, but taken together suggest that hominins contributed, albeit slightly, to the formation of the faunal assemblage recovered from the cave. It is important to note that there are few differences between layer IIIc and the sublayers of Unit IV. On the one hand, around 5% of the remains from both layers are burned, and a more exhaustive analysis is necessary to be able to determine whether these elements were part of the hominin diet at Foradada. Only one cut mark, on the spinous process of a cervid vertebra from layer IIIc, points to the exploitation of large mammals at the site, while no cut-marked bones of macromammals have been recovered from Unit IV. However, in both cases it is clear that there was an important consumption of small game, specifically rabbits. Although cut marks are only present on one tibia fragment from layer IV (Fig 18), the presence of midshaft cylinders—an exclusively anthropogenic pattern [105–107]—often burned and with notches from what are possibly human bite marks, are the main elements that enable us to interpret the remains as being of anthropogenic origin. Humerus, tibia, and femur cylinders represent 17% of the midshaft cylinders in layer IIIc, and 25% in layer IV, percentages similar to those found in other Iberian Upper Paleolithic assemblages [105]. This type of small game consumption has also been documented at other Châtelperronian and Early Aurignacian sites [108], although Cova Foradada is the first case where it has been documented with such intensity. Apart from this, as noted earlier, one eagle pedal phalanx from layer IV1 presents a series of cut marks that are not related with nutritional purposes [100].

### Radiocarbon results

Short human occupations in caves produce low quantities of clearly anthropogenic remains, particularly bones, so the precise dating of the Cova Foradada Pleistocene sequence presents a serious challenge. In this way, the first ^14^C dating analyses were planned with a two-fold intention: to characterize the age of the human occupations; and to test the taphonomic integrity of the different units.

The currently available dating results are described in Table 4. The single sample dated from layer IIIn resulted in an age of 26.5 ± 0.1 ^14^C ka BP, 31.0–30.6 ka ^14^C cal BP.

**Table 4:** C-14 dates available for the Cova Foradada sequence. G = Gravettian, EA = Early Aurignacian, CP = Châtelperronian. The subsample column indicates those dates obtained from various fragments of the main sample.

Layer	Attribution	Subsample	Method	Pretreat.	Sample	Taxon	Lab-ID	^14^C Age	SD	Unmodelled BP (95.4%)
IIIn	G		^14^C AMS	Acid digestion	Seashell bead	*Homalopoma sanguineum*	OxA-24646	26570	120	31045–30610
IIIc	EA		^14^C AMS	AAA	Charcoal	Juniperus	Beta-378800	30220	180	34620–33902
IIIc	EA	1(a)	^14^C AMS	AAA	Charcoal	*Pinus sylvestris*	Beta-378801	31690	180	36050–35120
IIIc	EA	1 (b)	^14^C AMS	ABOx-SC	Charcoal	*Pinus sylvestris*	OxA-34233	33170	370	38406–36402
IIIc	EA	2 (a)	^14^C AMS	AAA	Charcoal	Juniperus	Beta-414540	30770	180	35063–34300
IIIc	EA	2 (b)	^14^C AMS	ABOx-SC	Charcoal	Juniperus	OxA-34251	32050	550	37638–34830
IIIc	EA		^14^C AMS	Ultrafiltration	Bone	*Lynx pardinus*	MAMS-33909	30760	150	35009–34341
IV	CP	3 (a)	^14^C AMS	AOx-SC	Charcoal	*Pinus sylvestris*	OxA-X-2649-9	34490	320	39774–38401
IV	CP	3 (b)	^14^C AMS	AAA	Charcoal	*Pinus sylvestris*	Beta-435465	34570	240	39679–38553
IV	CP		^14^C AMS	AOx-SC	Charcoal	Conifera indet	OxA-X-2650-9	34300	1000	41138–36445
IV1	CP		^14^C AMS	AAA	Charcoal	*Pinus sylvestris*	Beta-414539	31900	200	36255–35325
III/IV	EA / CP contact– SW Gallery		^14^C AMS	ABOx-SC	Charcoal	*Pinus sylvestris*	OxA-34232	33050	350	38300–36327
III/IV	EA / CP contact– SW Gallery		^14^C AMS	ABOx-SC	Charcoal	*Pinus sylvestris*	OxA-34250	33050	400	38375–36265

The results from layer IIIc provided two different date clusters. On the one hand, the date given for the lynx bone and AAA samples ranged between 30.2 and 31.7 ^14^C ka BP. On the other, the ABOX-sc samples resulted in an age of between 32.0 and 33.1 ^14^C ka BP. The 95.4% probability calibrated chronology of the younger age cluster places the Aurignacian occupations at between 33.9 and 36.0 ^14^C cal BP. On the other hand, the ABOX-sc calibrated age points to an occupation during GI-8, between 38.4 and 34.8 ^14^C cal BP. The dual distribution of radiocarbon ages from layer IIIc seems to be linked to the charcoal pretreatment method, with the AAA dates being ca. 1.5 millennia younger than those for ABOX-sc when the same split sample is considered. Finally, the MAMS-33909 age from a carnivore remain must be considered a chronological indicator of the sedimentological formation of layer IIIc, but not necessarily related to the timing of the human occupation.

Two ABOX-sc samples from the SW Gallery provided identical ages of 33.0 ^14^C ka BP, falling into the same range as the ABOX-sc dates from layer IIIc. As stated previously, the SW Gallery presented an unclear stratigraphy dominated by large boulders and scarce sedimentary matrix, hindering correlation with the main excavation area. The samples, taken from a depth corresponding to the base of layer IIIc, were submitted to ascertain the lateral continuity between the two sectors.

In the case of layer IV, both ABOX-sc and AAA dates for the same split sample resulted in almost identical central values of 34.5 ^14^C ka BP; an additional sample, resulting in a date of 34.3 ± 1.0 ^14^C ka BP, should be considered a minimum age due to a low carbon yield after sample combustion. The 95.4% probability calibrated ages of these dates provide central values of 39.0 ^14^C ka cal BP. In addition, a single unsplit AAA sample from sublayer IV1 yielded a date of 31.9 ± 0.2 ^14^C ka BP or 36.2–35.3 ^14^C ka cal BP, falling within the range of AAA dates from layer IIIc, and probably resulting from vertical charcoal mobility.

**Fig 19:**
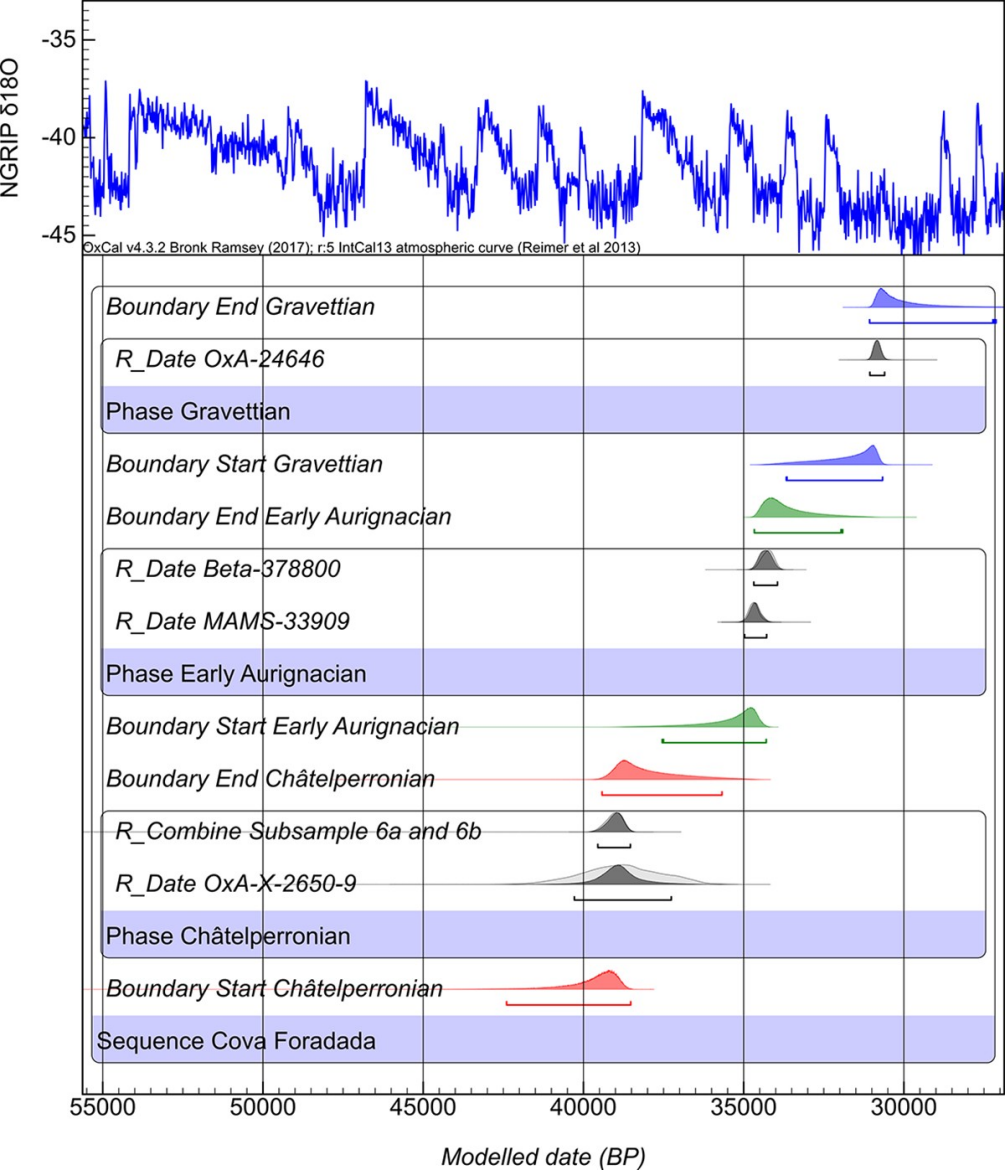
Modelling of radiocarbon dates from Cova Foradada. Colored distributions highlights end and start boundaries for the formation of each layer. Calibrated against Intcal’13 in Oxcal v.4.3.2.

Considering these results, a conservative Bayesian model was constructed to better understand the chronological sequence at Cova Foradada (Fig 19 and S2 Supporting Information). Given the discrepancy between the AAA and ABOX-sc dates, and the difficulty of dating clearly occupation-related remains, the goal of the model was to establish an *ante quem* chronology for the deposition of the sedimentary layers containing remains from both human and carnivore occupations; this should, therefore, be interpreted as providing minimum dates for the human occupations in each layer. Further work is necessary to define a more accurate and reliable chronology for the human occupations at Cova Foradada.

The OxCal R_Combine function was performed on the split samples. Subsamples 1a and b and 2a and b, all from layer IIIc, did not pass the X2-Test, hence they were not included in this model. On the other hand, subsamples 3a and b, from layer IV, are statistically identical and computed in the model. ABOX-sc dates from the SW Gallery were also excluded from the modelling at this time because of their stratigraphic uncertainty. Thus, according to the model (Fig 19 and S3 Supporting Information), layer IV finished forming at at least 35.7 ^14^C ka cal BP, layer IIIc at 34.6 ^14^C ka cal BP, and layer IIIn at 31.0 ^14^C ka cal BP. On the other hand, the upper boundaries of the modelled probability distributions place Unit IV at between 42.2 and 38.5 ^14^C ka cal BP, layer IIIc between 39.4 and 35.6 ^14^C ka cal BP, and layer IIIn between 33.6 and 30.6 ^14^C ka cal BP, however this latter point should be confirmed with new dates generated using materials other than marine shells.

## Discussion

The Cova Foradada sequence provides important insights into the narrative of the Late Neanderthal–Modern Human substitution in southern Europe, including Mediterranean Iberia within the regions of interest for the Middle-to-Upper Paleolithic Transition. The claimed appearance of both the Châtelperronian and Early Aurignacian industries provides the first unambiguous evidence of the occupation of a Mediterranean coastal environment, thereby extending the boundary of the Châtelperronian and Early Aurignacian transition southwards from the Pyrenean and Cantabrian mountain ranges.

This work reports the first pre-Aurignacian assemblage with backed pointed blades found south of the Cantabrian and Pyrenean belt. The exceptional nature of this finding and its geographical implications for the Neanderthal–Modern Human replacement requires a thorough analysis starting from the taxonomy of the assemblages, their phylogeny, chronology and the way they fit into regional evolutionary models.

The main feature of the lithic assemblage from Unit IV from Cova Foradada is the prominent overrepresentation of retouched tools, and within this assemblage, the presence of eight blades or blade fragments with unilateral abrupt retouching, configuring convex edge delineations and pointed tips. Considering the general shaping objectives and the way they were specifically implemented on each blade, the backed tools from Foradada fit the original definition of typical/atypical Châtelperronian points or knives [97]. One feature observed in Unit IV, is the lack of standardization of these blades, at least at the time they were discarded, showing that the desired morphology and characteristics of the tools were attained following various modalities adapted to the morphologies of the blanks. On the one hand, this could be related to expedient technological behavior related to the very-short term, and probably task-specific, occupations attested at Cova Foradada. The internal variability of the raw material, each blade coming from a different group of raw materials, could also have been necessitated by technical adaptation to raw material constraints. However, variability in retouching intensity and blank size has been reported extensively from many other Châtelperronian assemblages, including Grotte de la Verpillière I [95], Quinçay [30], Roc-de-Combe et La Côte [15], and Aranbaltza [40]. In addition, the typometric results are comparable to those observed by Roussel et al. [30] in assemblages such as those from Quinçay and other French sites mentioned therein, as well as the systematically reported average dimensions of Châtelperronian points [15,95,109,110]. The absence of a larger technological assemblage from Unit IV prevents the contextualization of these tools in their specific technological context, and only tool attributes provide insights into the reduction sequences related with their production. At this point, the transversal asymmetry of some (Fig 15A–15D; Fig 16B and 16C) and the reported presence of core convexity, produced from opposite platforms, are considered characteristic features of the Châtelperronian blade production systems [15,36,111,112].

On the other hand, this tool type is reported to be a *fossil directeur* exclusively related to the Châtelperronian culture [15], being absent from both the Protoaurignacian and Early Aurignacian assemblages, where large blades are generally shaped into domestic tools like end-scrapers or Aurignacian blades with scalar retouching [111], but which do not usually present backed retouching and pointed morphologies. Furthermore, the relative abundance of retouched blades from the Protoaurignacian is significantly low, as it is generally a bladelet-dominated technocomplex [113,114]. The possibility of asserting Protoaurignacian occupations exclusively through the presence of abruptly retouched and pointed blades is quite odd, independently of the fact that these tools could appear sporadically within Aurignacian laminar assemblages, as reported from the Leroi-Gourhan excavations at Grotte du Renne [115] or listed in the typological lists from Morin [116]. Considering the typological, typometric, technological, and stratigraphic evidence, the eight points recovered so far from Cova Foradada’s Unit IV represents one of the largest assemblages of Châtelperronian points in Iberia, also providing a reliable chrono-cultural attribution for the documented human occupations.

It is far beyond both the scope of this paper and the potential of the evidence currently available from Cova Foradada to discuss the taxonomy of the Châtelperronian makers and the demographic, ecological, or cultural drifts driving the appearance of transitional assemblages [19,31]. However, at a technological level, evidence for the rooting of some transitional technocomplexes, including the Châtelperronian, in earlier Middle Paleolithic traditions seems to be less controversial.

Volumetric reduction methods are mentioned as early as MIS-5 Neanderthal-made industries [117]. However, the appearance of abruptly retouched blades detached from volumetric laminar cores, is a technological innovation that some authors links with the final Middle Paleolithic of central and southern France, namely the Mousterian of Acheulean Tradition [15,118]. Independently of the geographical co-occurrence, the direct relationship between the reduction technique and the desired products for the shaping of a specific tool-type is proposed to connect the Mousterian of Acheulean Tradition and the Châtelperronian Upper Paleolithic-like industries [14,17,119].

However, backing retouch is not actually an exclusive trait of Châtelperronian industries, they manifest as a common trait of southern European transitional assemblages. Usually smaller than Châtelperronian points, abruptly retouched lunates are a distinctive feature of Uluzzian assemblages from the central Mediterranean area [120]. The Uluzzian displays a flake-dominated technology with a blade/bladelet component and a significant presence of splintered tools [8, 121]. It is claimed that there is a technological break between the Uluzzian and the last Middle Paleolithic occupations, particularly at Grotta di Fumane where the Middle Paleolithic A5-A6 units exhibits a unipolar or laminar Levallois tradition and the Final Mousterian—Uluzzian complex A4-A3 units is dominated by centripetal recurrent Levallois in A4 and by a variety of flake knapping technologies in A3 [7,121].

Independently of the causes triggering these shifts in both the Châtelperronian and Uluzzian technocomplexes, technological connections with the local Middle Paleolithic assemblages, despite being controversial, are present in both narratives. There is not a great deal of evidence linking the transitional techno-complexes documented in Iberia with the local Middle Paleolithic, mostly because of the scarcity of evidence and lack of large enough assemblages. The appearance of volumetric Upper Paleolithic-like bladelet strategies during the Cantabrian late Middle Paleolithic has been proposed as a progressive trend resulting in the controversial Transitional Aurignacian from layer 18 of Cueva del Castillo [122,123,33,124,125] formed at least 44.9–42.1 ^14^C ka cal BP [41]. In the few Iberian sites with Châtelperronian assemblages, these occupations lie at the base of the stratigraphic sequence, at Labeko Koba and Ekain [37], and late Middle Paleolithic occupations have not yet been reported from Aranbaltza [126]. At Cueva Morin, a Discoid-Levallois late Middle Paleolithic technology was reported for layers 11–12, where a small laminar component was also described [127,128]. In the NE of Iberia, isolated Châtelperronian artifacts have been recovered from Ermitons and L’Arbreda, occurring in recurrent centripetal Levallois assemblages, leading them to be defined as Mousterian with Châtelperronian points [129,130]. In addition, a poorly diagnostic early Upper Paleolithic assemblage from layer 497D at Cova Gran de Santa Linya has been cautiously related to the Châtelperronian from other locations [131,132]. 497D overlies the Middle Paleolithic layer 1B characterized by recurrent centripetal Levallois technology. Putative Châtelperronian points from Abric Romaní layer A have been also been reported [133] overlying the late Middle Paleolithic occupations in layer B, but this attribution does not seem to be supported by data and formal lithic analysis [134]. It is remarkable that any evidence of MTA Middle Paleolithic technocomplexes has ever been found in NE or Mediterranean Iberia. This is clearly illustrated by the entire Middle Paleolithic sequence from Abric Romaní, ranging from the MIS-5 [135] to ca. 45 ka BP [35,136], and providing an alternation of Levallois and Discoid-oriented occupations [137,138]. The same is true of the latest Middle Paleolithic occupations from sites such as Cova Gran de Santa Linya [131,139], Roca dels Bous [140], Teixoneres [141,142], and L’Arbreda [143].

The technological characterization of the Châtelperronian layers reported from northern Iberia appears to be very unbalanced. L’Arbreda and Ermitons do not seem to clearly represent defined Châtelperronian occupations, but there is an aggregation of a small Châtelperronian component at the top of the Middle Paleolithic layers. At Labeko Koba and Ekain, both interpreted as hunting stands [37], technological information is limited by the small size of the assemblages, and the formation processes bear a striking resemblance to those described for Cova Foradada Unit IV. In layer IXinf at Labeko Koba, the reduction sequence has been proposed to be strictly laminar, the flakes in the assemblage resulting from core configuration or derived from hammerstone breakage. Only eleven lithic remains have been recovered from layer Xa at Ekain, six of these being retouched blades or bladelets. In layer 497D from Cova Gran de Santa Linya the flaking strategies are also strongly blade oriented, and reduction from single and opposed platforms prismatic cores [144] dominates the assemblage, while Discoid or Levallois cores characterizing the underlying Middle Paleolithic occupations are not documented [132]. Châtelperronian assemblages from Aranbaltza and Morin layer 10 are surely the largest well-defined collections available, showing both a preponderance of structured, volumetric laminar strategies, dominated by laminar cores, but with centripetal flake cores and micro-Levallois production also evidenced [36,40].

One of the main issues of the data from Cova Foradada Unit IV is related to the chronology of the layer formation and the occurrence of human occupation. The widest distribution of the modelled boundaries places the formation of Unit IV between 42.2 and 35.7 ^14^C ka cal BP, a timespan partially overlapping the youngest boundary for the Châtelperronian temporal distribution across SW Europe, proposed between 45 and 41 ^14^C ka cal BP [5,41,42,144,145,146]. According to the data currently available from NE Iberia, the end of Middle Paleolithic is at 43.0–41.8 ^14^C ka cal BP in L’Arbreda layer H [41], at 43.2–42.0 ^14^C ka cal BP in Cova Gran layer 1B [130,138], at 45.09–43.8 ^14^C ka cal BP in Teixoneres Cave [142], and at 43.2–42.2 ^14^C ka cal BP in Abric Romaní. The general trend of this distributions indicates that the last Middle Paleolithic Neanderthal occupations took place sometime between 43.8 and 41.8 ^14^C ka cal BP.

At this point, the 42.2–38.5 ^14^C ka cal BP start boundary provided by the modelling dates from Unit IV suggests a consistent succession between the end of the NE Iberian Middle Paleolithic and the appearance of the Châtelperronian. The unmodelled upper distribution of dates from 497D at Cova Gran, 39.3–38.1 ^14^C ka cal BP [131], also seems to be consistent with that. Furthermore, the dating of the Protoaurignacian occupations at L’Arbreda, 42.2–41.0 ^14^C ka cal BP [41], and Abric Romaní, 42.3–41.2 ^14^C ka cal BP [133], suggests a possible chronological overlap of the Protoaurignacian and Châtelperronian in NE Iberia during the immediate period after the last Middle Paleolithic occupations. Within this context, the significance of the Châtelperronian at Cova Foradada will be even greater if a more precise dating of the geological dynamics and the ephemerous human occupations from Unit IV is soon achieved. The currently available dates are younger than the accepted range for the Châtelperronian and, could be related to a late, southward demographic migration of Châtelperronian groups from north of the Pyrenees. However, dates can be a direct proxy of the human occupations, but also be rejuvenated by modern carbon contamination or proceed from percolated samples, so further dating is needed to obtain more consistent arguments. Refining the chronology of Unit IV with further ^14^C dating, in addition to the ongoing OSL programs, and the upcoming studies from layer 497D at Cova Gran de Santa Linya will play a pivotal role in characterizing the southernmost territories where the succession between the Middle Paleolithic, Châtelperronian, and Protoaurignacian is recorded. To date, evidence for the Middle-to-Upper Paleolithic transition found at a more southerly latitude than that of Cova Foradada, both in Mediterranean and Central Iberia, has not pinpointed the existence of the earliest Aurignacian phases or transitional assemblages.

The recently published assignation of Bj/13 from Bajondillo cave in Málaga (southern Iberia) as Aurignacian [147] still requires further technological assessment as there is a lack of diagnostic Aurignacian tool-types, the known blade and bladelet production strategies appearing during the latest Middle Paleolithic at sites such as El Castillo and Mirón, in addition to the blade and bladelet strategies ubiquitously documented during the Châtelperronian. Even so, the dates obtained for Bj/13, of 43–42 ^14^C ka cal BP, could be even older than the Protoaurignacian occupations at Abric Romaní and L’Arbreda, as well as most of the Aurignacian evidence found elsewhere in Europe [41]. While the cultural attribution of Bj/13 is still to be confirmed or refuted by further excavation, the late dispersal scenario for Modern Human expansion into southern Iberia suggested by the Ebro Frontier model [45,148] is still prevalent. To trace the timing of an earlier or later advance of Modern Humans towards the south, it became evident than robust chronological models are required for the Cova Foradada Aurignacian and Gravettian occupations. According only to the chronology of the younger date cluster places the layer IIIc occupations at 36.0–33.9 ^14^C cal BP, quite recent for the Early Aurignacian and probably fitting with the latest occurrences of SBP in the Aurignacian record [91]. On the other hand, the ABOX-sc calibrated ages point to an occupation during GI-8, between 38.4 and 34.8 ^14C^ cal BP, correlating more closely with the Early Aurignacian chronology inferred from directly dated SBPs at Mere Clochette, Spy, Brillenhohle, and Tischoferhohle [91]. According to recently published compilations [149], the date from layer IIIn, 26.5 ± 01 ^14^C ka bp, or 31.0–30.6 ka ^14^C cal BP, represents one of the earliest appearances of the Gravettian in Mediterranean Iberia. However, the problems associated with marine shell dating [150,151] and the absence of further dates from this level necessitates a cautious interpretation of the layer’s antiquity.

Related to this, a point of interest is the attribution of Cova Foradada layer IIIc to the Early Aurignacian, especially considering the current uncertainty of the radiocarbon dates. This attribution is mostly based on the interpretation of the antler point fragments as SBPs [87–89,91,152–153]. As stated before, the main arguments for these fragments being interpreted as SBPs are related to morphometric and technological attributes deriving from the analysis, namely the work on the point surface, cross-section, and maintenance and hafting system. These points were likely to have been discarded as they had reached the functional size limits for the points. Amongst the almost 150 complete SBPs from European Aurignacian sites in France, Spain, and Italy, only two specimens are shorter than 40 mm and only nine are shorter than 50 mm. Even in such cases, the width of the points remains quite regular, always being more than 10 mm, with the exception of one specimen from La Quina-Aval (France). Within the chronological span of layer IIIc, the SBPs constitute the main organic projectile points documented in the European archaeological record, while in the Near East, the Levantine Aurignacian is characterized by simple/massive antler points [85,99,154,155,156]. These simple/massive bone points are only documented in the European record from the Evolved Aurignacian, when SBPs disappear [87,90], and clearly differ morphologically and morphometrically from the SBPs. The elliptical cross-section commonly displayed by the SBPs, as in the specimens from Foradada, becomes mainly oval or circular in the simple/massive points. Also, the hafting system of the two projectile categories, typologically defining them, makes the attribution of the Foradada specimens clear. Therefore, there can be no doubt about the classification of the Foradada specimens, or the integration of the technical piece, namely the blank, as Early Aurignacian antler exploitation schemes, characteristic of south-western and central Europe. Recent work on the technical aspects of antler exploitation at the beginning of the Early Upper Paleolithic in Eurasia utilizes longitudinal splitting as a technical marker of the Aurignacian antler working system. Although this practice is also associated with Gravettian contexts [157], this type of debitage is especially characteristic of the Early Aurignacian, being the only debitage procedure employed to obtain SBP blanks. The artifact demonstrating the use of this procedure at Cova Foradada is the “baguette”-type blank. Nevertheless, given the scarcity of archeological remains and the dispersion within the radiocarbon dataset, the possibility of some Evolved Aurignacian occupation events occurring within the formation time span of layer IIIc cannot be dismissed entirely.

The exiguity of occupation-related remains is also a noteworthy aspect of the Cova Foradada sequence. The record from the extensive excavation of the Châtelperronian and Early Aurignacian layers denotes recurrence of a very specific use of the cave for very short time periods, an aspect that is coincident with other Iberian Middle-to-Upper Paleolithic sequences [37]. This is evidenced by the lithic record, which demonstrates a probable lack of *in situ* knapping episodes, as well as by the faunal assemblage, where the anthropogenic impact on bones is almost negligible. The overrepresentation of retouched tools, the high ratio of tool use, the raw material variability, and the presence of broken antler projectiles in layer IIIc, point to a shared specific occupation pattern during both periods. A crucial question deriving from this is whether Cova Foradada represents a logistic, or even opportunistic, use of the cave within the overall mobility pattern of well-established hunter-gatherers, or whether it is direct evidence of a scarce territorial settlement. Considering the regional scarcity of Middle-to-Upper Paleolithic evidence, data from Cova Foradada could be consistent with an essentially low-density demographic pattern, or even combined with highly mobile groups not permanently settled in the area. In this context, the few cases already documented of an Aurignacian presence below the 42^nd^ parallel north in NE Iberia all have similarities to the dynamic seen at Cova Foradada. Layer A from Abric Romaní involves a lithic assemblage of a few hundred remains [134], including less than 50 retouched tools [158], and mimics the Aurignacian-Gravettian package from Cova Foradada Unit III. At Terrasses de la Riera dels Canyars, Daura et al. [159] reported the presence of some artifacts that included a single blade with Aurignacian retouching within a strongly carnivore-dominated open-air context dating back to 39.0–37.5 ^14^C ka cal BP. At Cova del Gegant, sporadic human presence evidenced both by fire structures and scarce lithic artifacts has been dated as Early Upper Paleolithic and Middle Paleolithic [160,161]. Our ongoing, unpublished excavations at La Griera and Cova del Trader sites, both within a 10 km radius of Cova Foradada, are also providing diagnostic Aurignacian artifacts associated with isolated fire structures or found in otherwise almost sterile layers.

The archaeological evidence recovered from Units IV and III at Cova Foradada represents the southernmost episode of Châtelperronian and Early Aurignacian documented to date in western Europe. This geographical expansion of the Middle-to-Upper Paleolithic Transition leads the way to new research in a territory where hypotheses on Neanderthal-Modern Human replacement have previously been proposed based on exiguous and fragmentary lines of evidence. Further research in the Penedès region will no doubt provide new and robust data that will aid our understanding of the Middle-to-Upper Paleolithic transition in western Europe.

## Supporting information

S1 Supporting Information Expanded information about the excavation process at Cova Foradada. (PPTX)

S2 Supporting Information CQL code of the Bayesian model. (DOCX)

S3 Supporting Information Results from the Bayesian chronological modelling. Tabulated results from the Bayesian model. (DOCX)

S1 Table Use-wear analysis. Percentage of lithics analyzed through high-power optical and digital microscopes and percentages of tools displaying any kind of use-related polishes. Hafting wear and post-depositional damage are not considered in the results.
(DOCX)

S1 Fig Unit III—IV stratigraphic contact. Detail of the stratigraphic contact between Units III and IV at the E9-D9 reference section (Fig 10A–10B and Fig 12 of the main text). a) View of the E9-D9 section, b) view of the E9-D9 section after image enhancement applying decorrelation stretch with the DStretch plugin for ImageJ (http://www.dstretch.com/), LXX colorspace. The enhancement clearly highlights the stratigraphic boundary between layer IIIc and the top of Unit IV (red). It also picks out combustion features from layer IIIc by identifying charcoal lines (blue) and altered sediments (magenta).
(JPG)

S2 Fig Examples of use-wear traces from Cova Foradada’s lithic assemblage. Scale bar represents 100μm in the microscope photos. 1. FO’97-IIIn-C7-815, denticulated flake with working traces of a medium-hard material, probably wood. The tool shows microchipping and intense polish and rounding along the edge on both sides. 1-Aa (100x), 1-Ab and 1B (200x) and 1-Ac (500x). 2. FO’13-III-F6-48. Scraper from the Aurignacian layer with intense rounding and polish provoked by its use on hard materials, possibly bone or antler. 2-A is a “gigapixel-like” image of the edge with more intense traces. 2-Aa shows the most intense polish, and 2Ab shows rounding, polish, and striation-like features. All images of this tool were taken at 200x.
(JPG)

S3 Fig Retouched bladelets from layer IIIc. Details of the marginally retouched edges of the bladelets from layer IIIc (Fig 13 3,4,8–10 of the main text). Panoramic views of the retouched edges have been processed using a KH8700 Hirox digital microscope at the IPHES lithic analysis laboratory. Scales are only applicable to magnified edges, numbered bladelets are in a relative scale.
(JPG)

S4 Fig Retouch comparison on blades from Unit IV. Comparative lateral views of the extent and distribution of retouching on the retouched blades from Unit IV. Lateral views of blades 1, 2, 3 and 8 have been mirrored for comparative purposes.
(JPG)

S5 Fig Diacritic schemes of the blades from Unit IV. 1–8 retouched blades or Châtelperronian points. 9–10 unretouched blades.
(JPG)
