# Supplementary material for: The Middle-to-Upper Paleolithic transition occupations from Cova Foradada (Calafell, NE Iberia)
Source: PLoS One. 2019 May 16;14(5):e0215832. doi: 10.1371/journal.pone.0215832 (PMC6522054; doi:10.1371/journal.pone.0215832)
Supplement: S1 Supporting Information — (PPTX) [file pone.0215832.s001.pptx]

## Slide 1
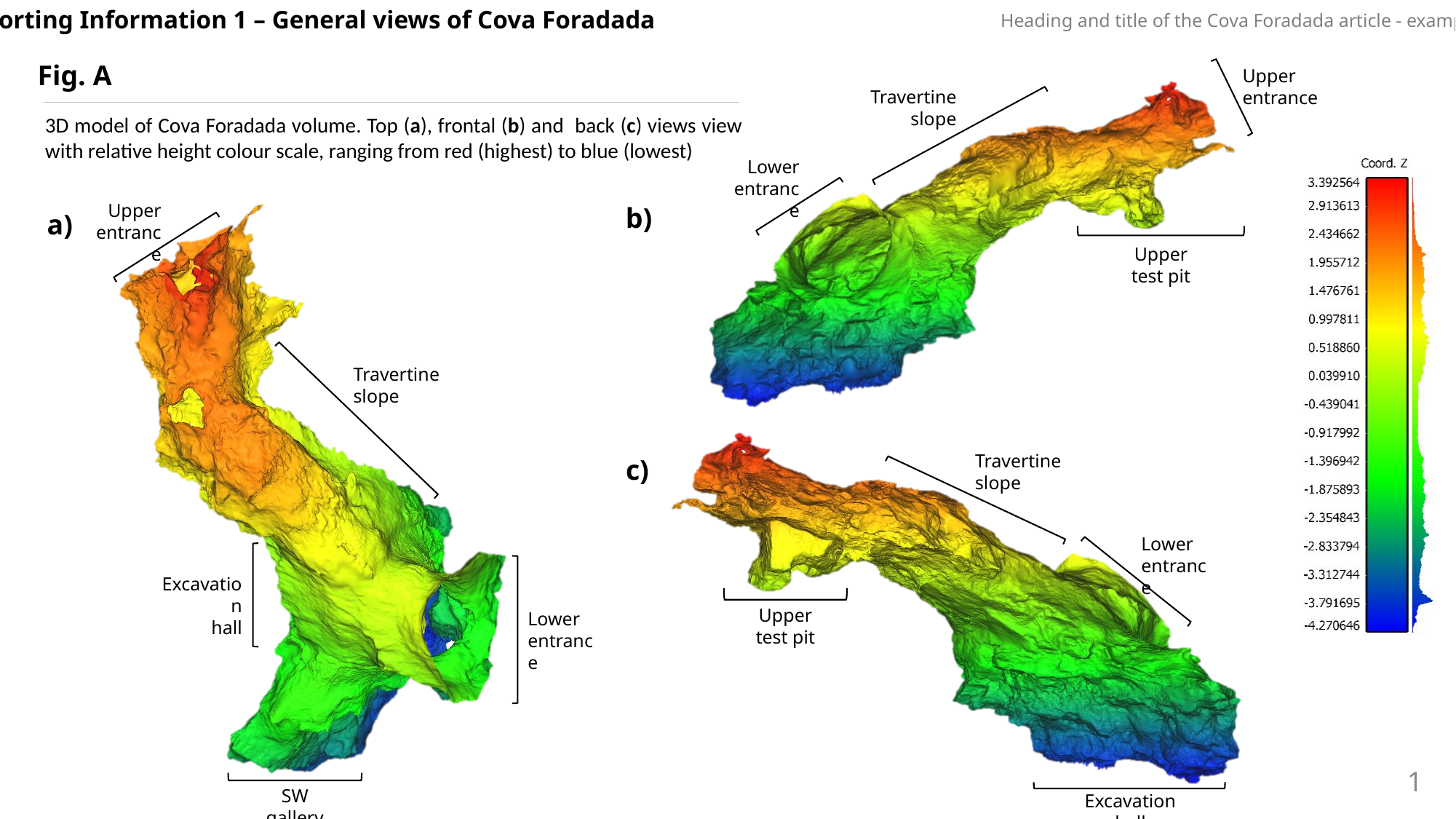

Supporting Information 1 – General views of Cova Foradada
Heading and title of the Cova Foradada article - example
Fig. A
Upper
entrance
Travertine slope
3D model of Cova Foradada volume. Top (a), frontal (b) and back (c) views view with relative height colour scale, ranging from red (highest) to blue (lowest)
Lower
entrance
Upper
entrance
Travertine slope
Excavation
hall
Lower
entrance
SW gallery
b)
a)
Upper
test pit
Travertine slope
Lower
entrance
Upper
test pit
Excavation hall
c)
1

## Slide 2
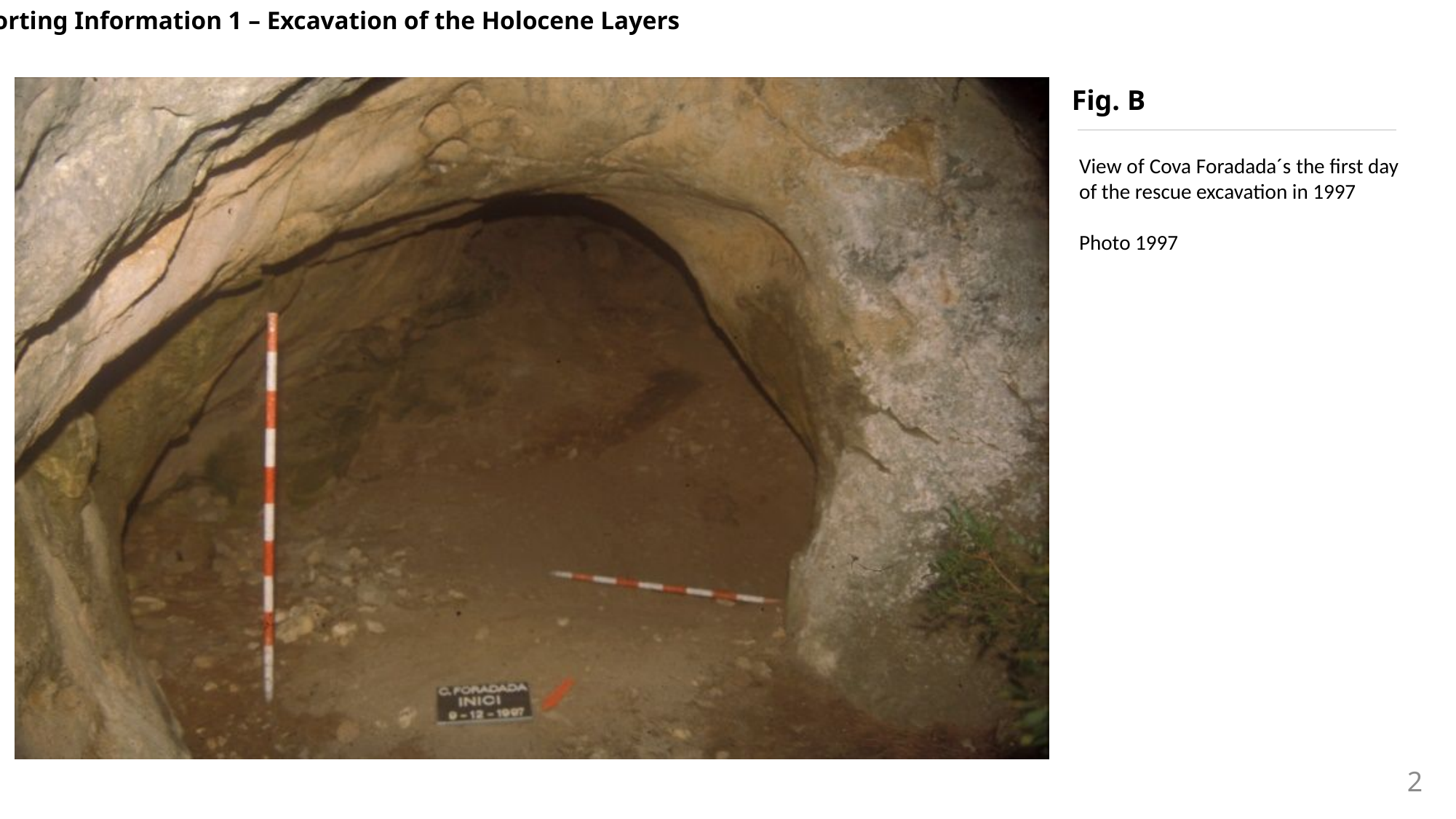

Supporting Information 1 – Excavation of the Holocene Layers
Fig. B
View of Cova Foradada´s the first day of the rescue excavation in 1997
Photo 1997
2

## Slide 3
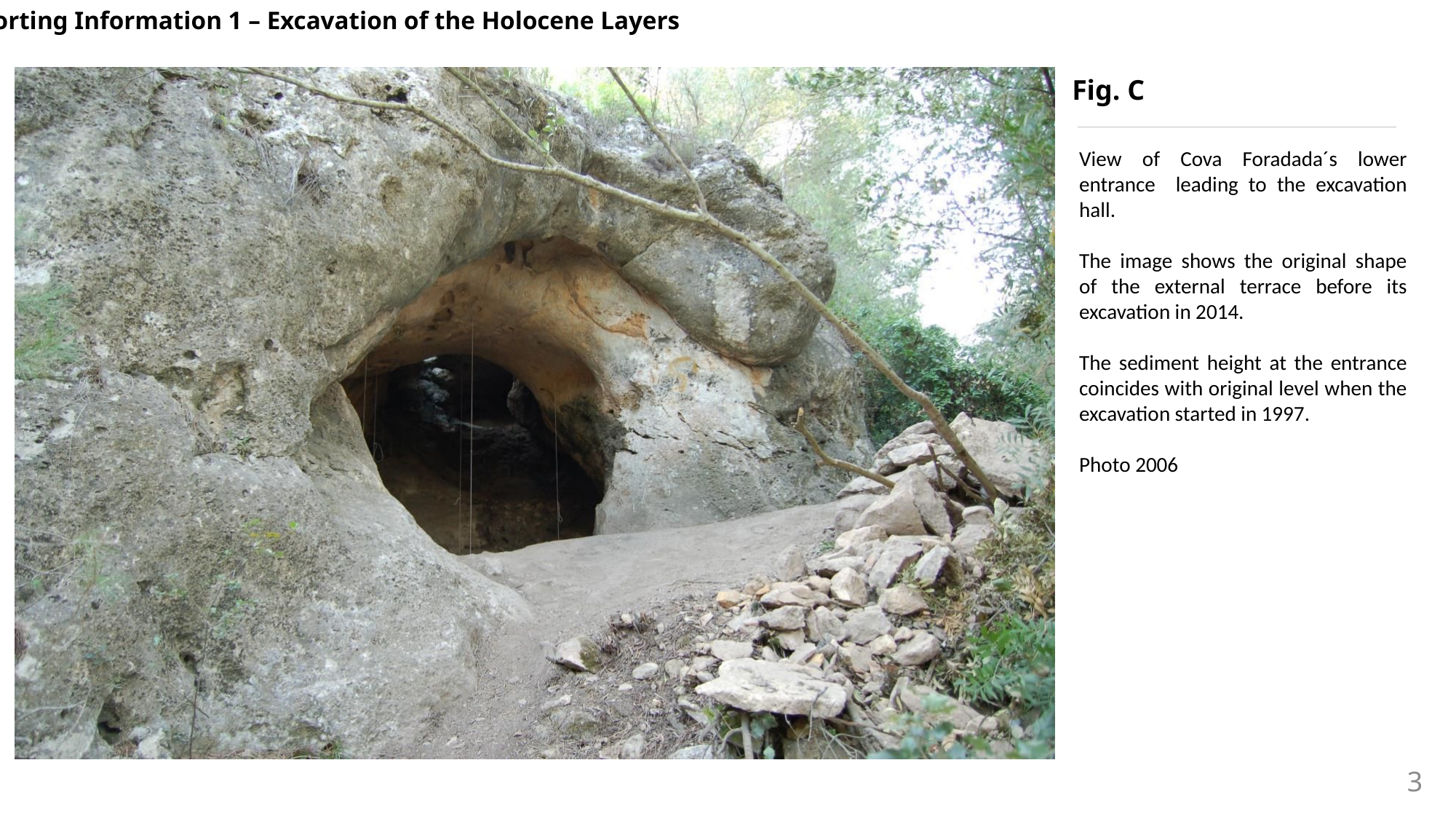

Supporting Information 1 – Excavation of the Holocene Layers
Fig. C
View of Cova Foradada´s lower entrance leading to the excavation hall.
The image shows the original shape of the external terrace before its excavation in 2014.
The sediment height at the entrance coincides with original level when the excavation started in 1997.
Photo 2006
3

## Slide 4
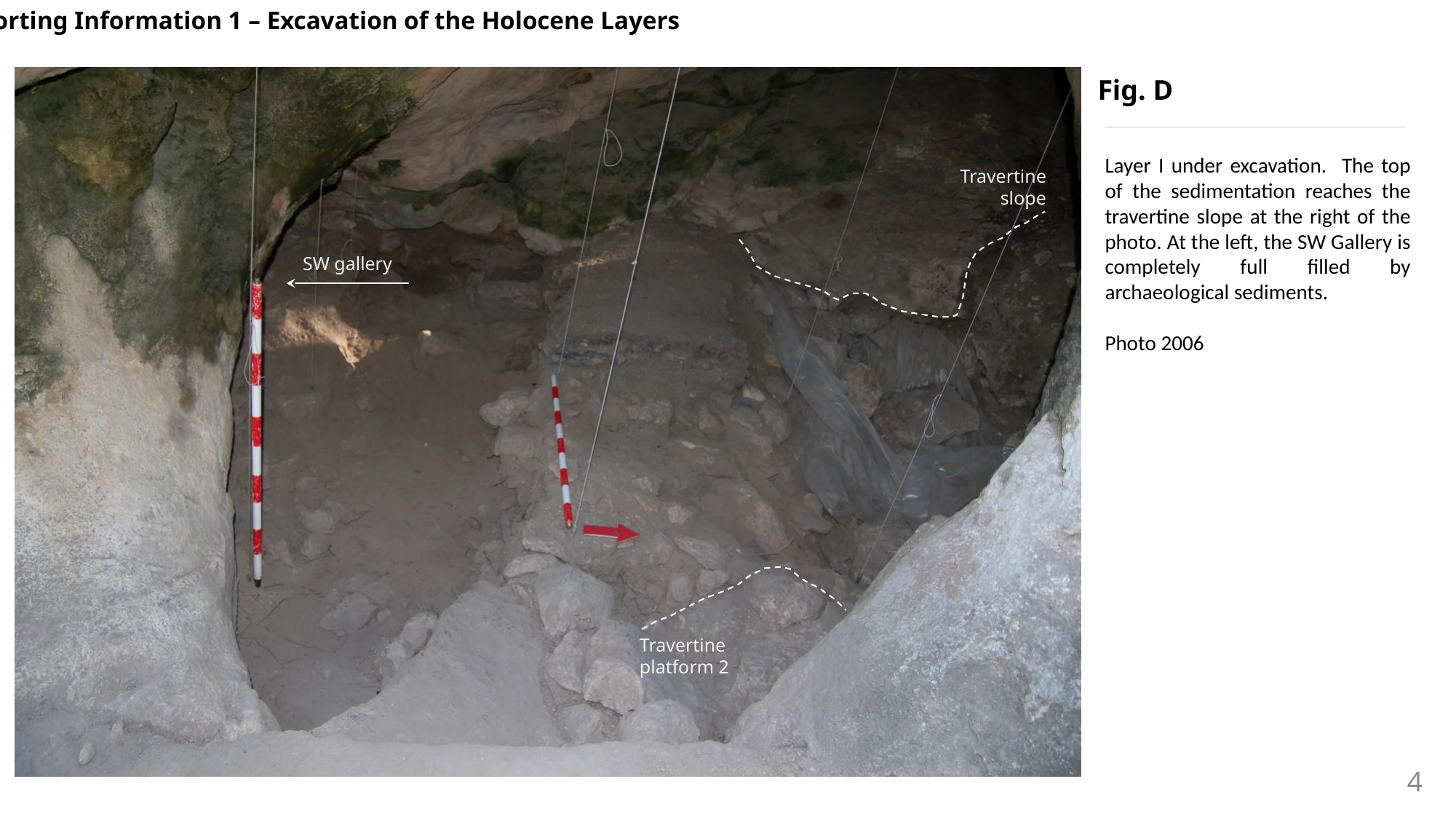

Supporting Information 1 – Excavation of the Holocene Layers
Fig. D
Layer I under excavation. The top of the sedimentation reaches the travertine slope at the right of the photo. At the left, the SW Gallery is completely full filled by archaeological sediments.
Photo 2006
Travertine
slope
SW gallery
Travertine
platform 2
4

## Slide 5
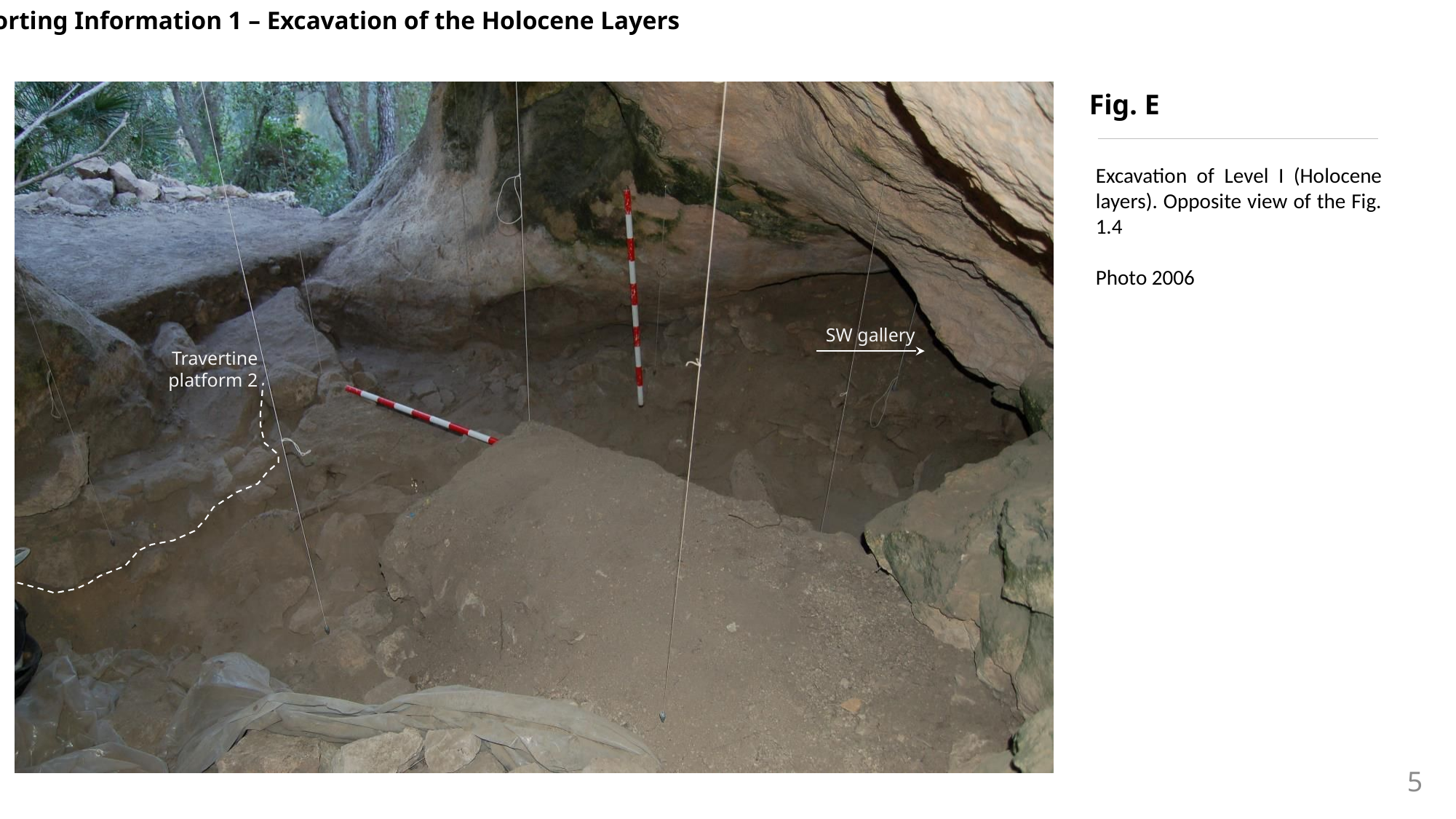

Supporting Information 1 – Excavation of the Holocene Layers
Fig. E
Excavation of Level I (Holocene layers). Opposite view of the Fig. 1.4
Photo 2006
SW gallery
Travertine
platform 2
5

## Slide 6
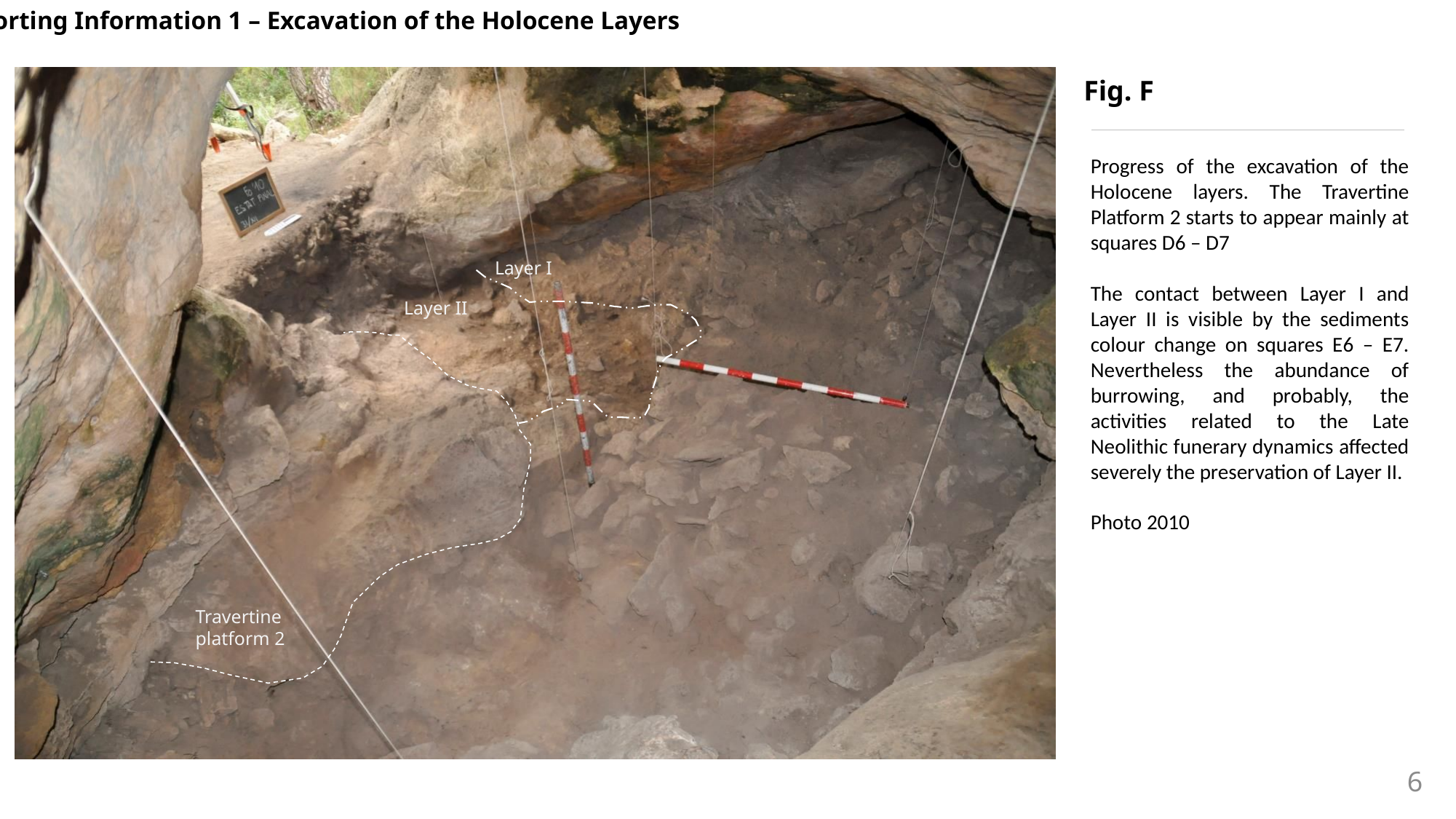

Supporting Information 1 – Excavation of the Holocene Layers
Fig. F
Progress of the excavation of the Holocene layers. The Travertine Platform 2 starts to appear mainly at squares D6 – D7
The contact between Layer I and Layer II is visible by the sediments colour change on squares E6 – E7. Nevertheless the abundance of burrowing, and probably, the activities related to the Late Neolithic funerary dynamics affected severely the preservation of Layer II.
Photo 2010
Layer I
Layer II
Travertine
platform 2
6

## Slide 7
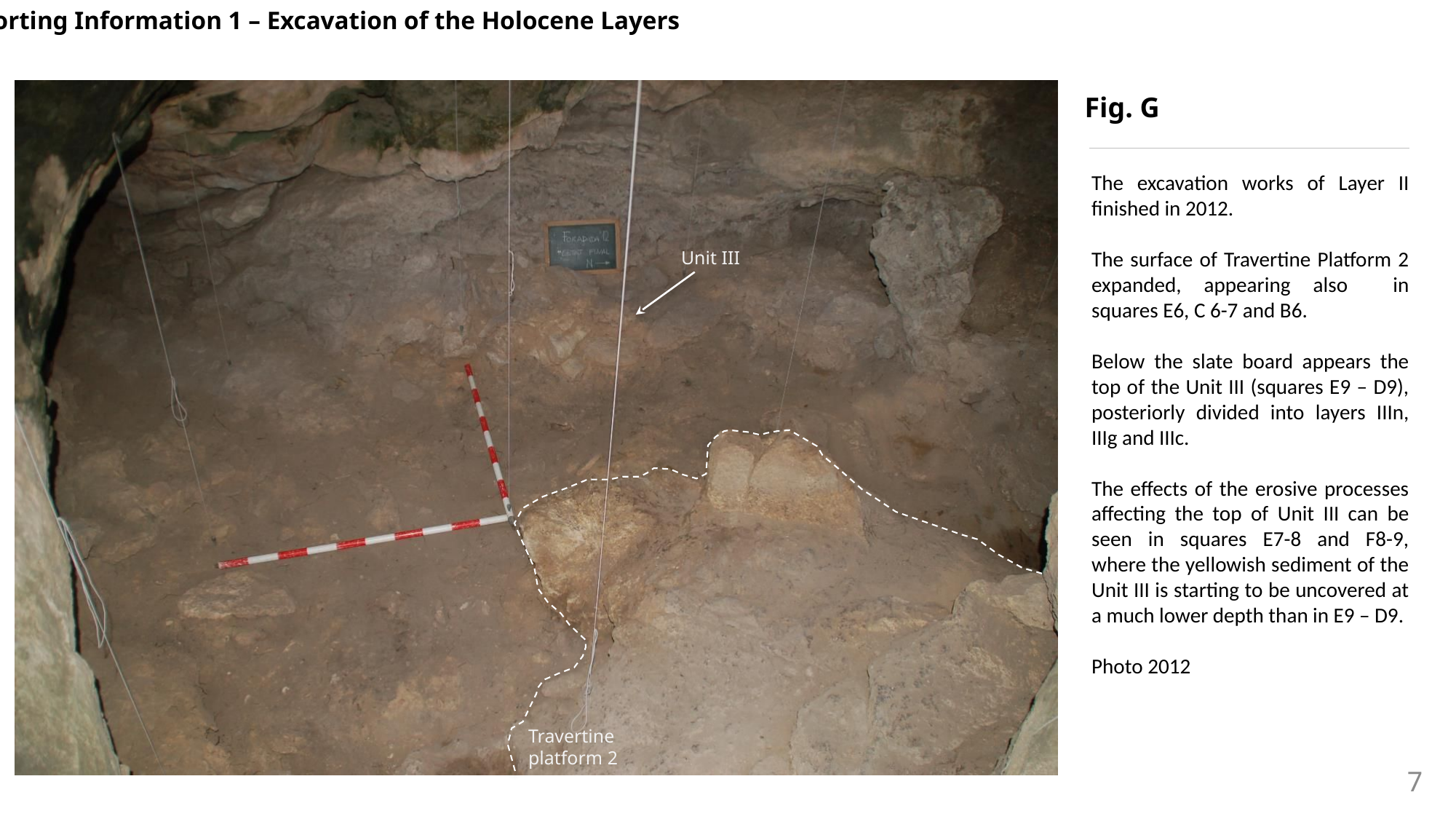

Supporting Information 1 – Excavation of the Holocene Layers
Fig. G
The excavation works of Layer II finished in 2012.
The surface of Travertine Platform 2 expanded, appearing also in squares E6, C 6-7 and B6.
Below the slate board appears the top of the Unit III (squares E9 – D9), posteriorly divided into layers IIIn, IIIg and IIIc.
The effects of the erosive processes affecting the top of Unit III can be seen in squares E7-8 and F8-9, where the yellowish sediment of the Unit III is starting to be uncovered at a much lower depth than in E9 – D9.
Photo 2012
Unit III
Travertine
platform 2
7

## Slide 8
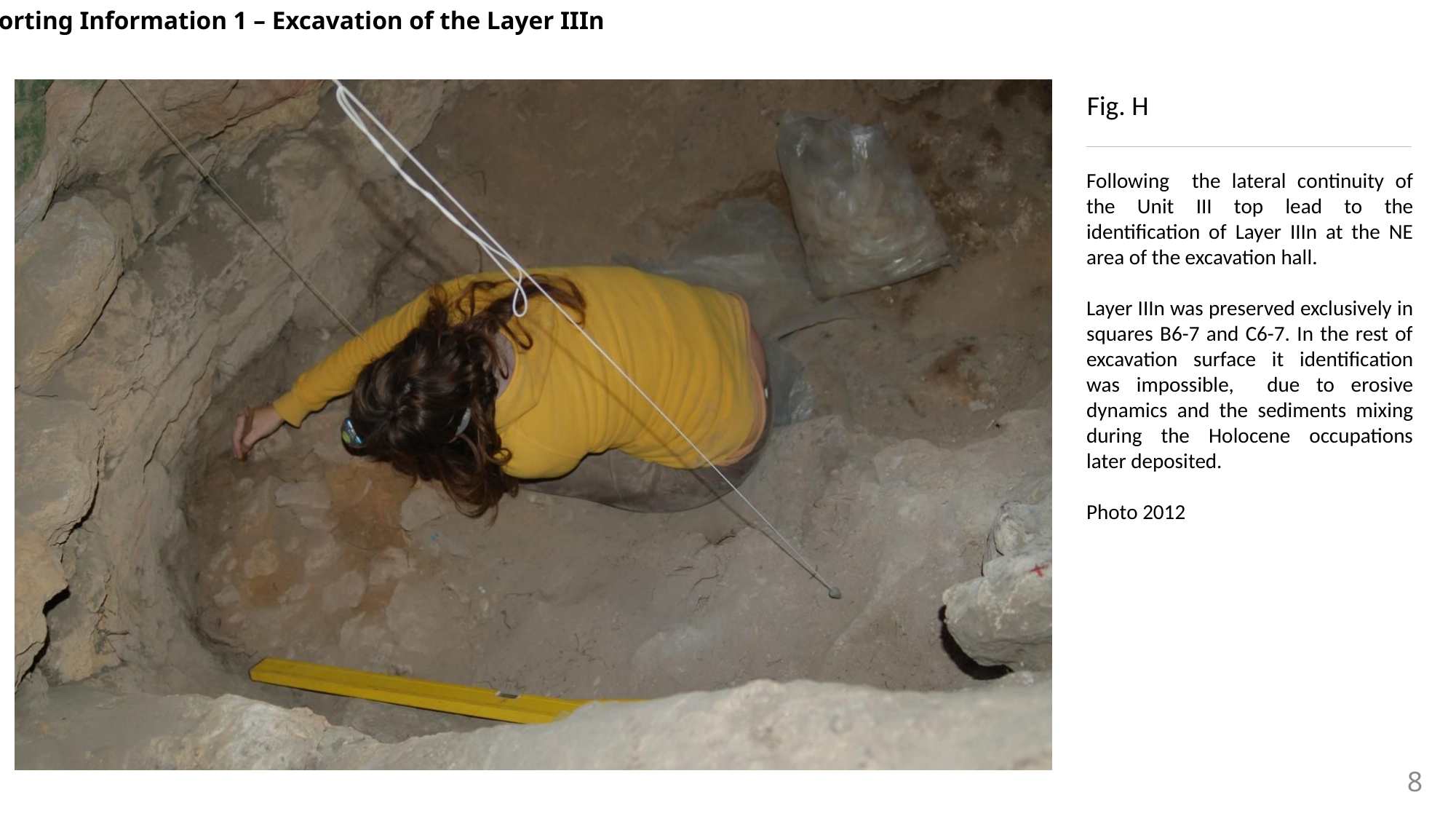

Supporting Information 1 – Excavation of the Layer IIIn
Fig. H
Following the lateral continuity of the Unit III top lead to the identification of Layer IIIn at the NE area of the excavation hall.
Layer IIIn was preserved exclusively in squares B6-7 and C6-7. In the rest of excavation surface it identification was impossible, due to erosive dynamics and the sediments mixing during the Holocene occupations later deposited.
Photo 2012
8

## Slide 9
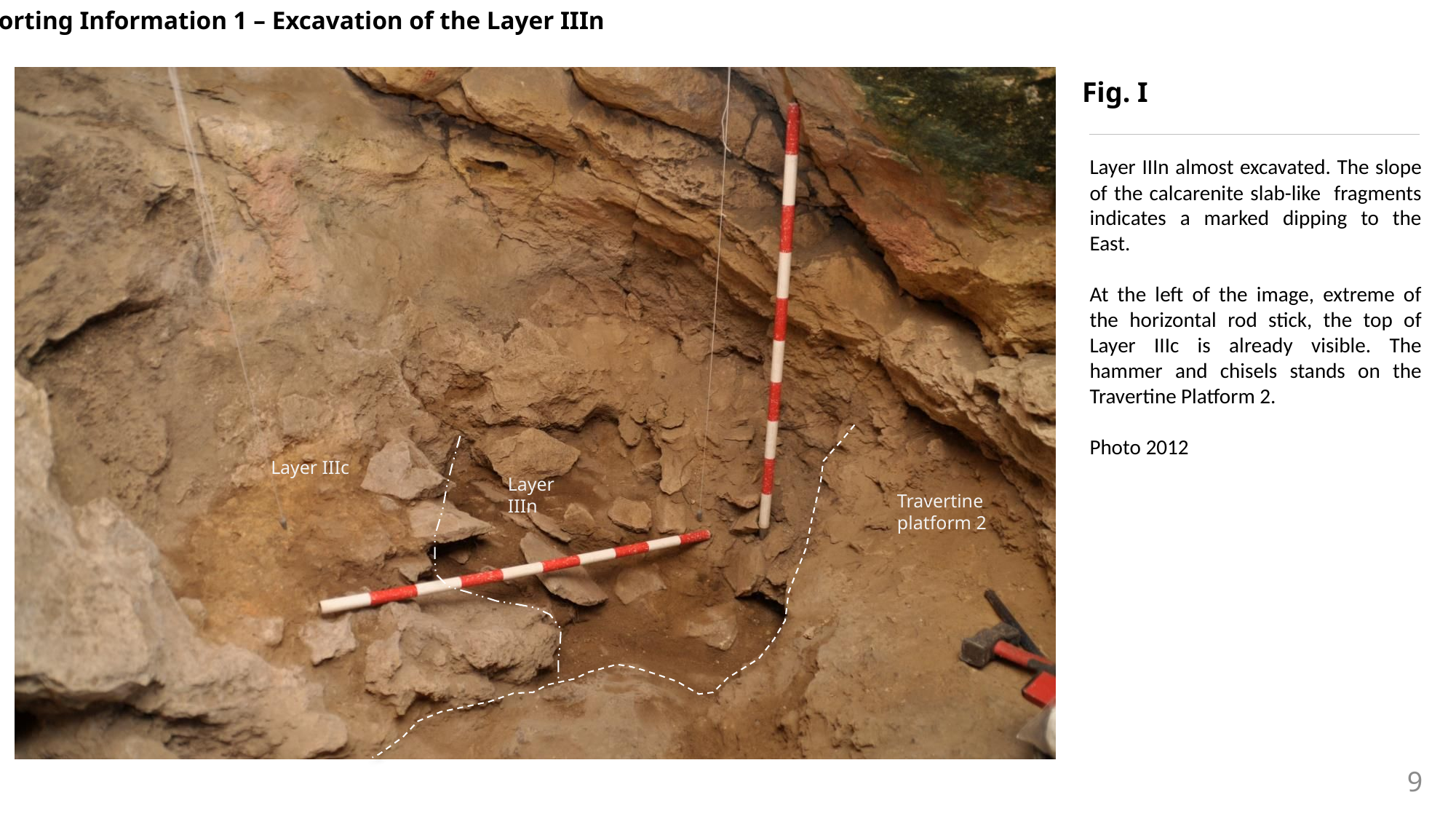

Supporting Information 1 – Excavation of the Layer IIIn
Fig. I
Layer IIIn almost excavated. The slope of the calcarenite slab-like fragments indicates a marked dipping to the East.
At the left of the image, extreme of the horizontal rod stick, the top of Layer IIIc is already visible. The hammer and chisels stands on the Travertine Platform 2.
Photo 2012
Layer IIIc
Layer IIIn
Travertine
platform 2
9

## Slide 10
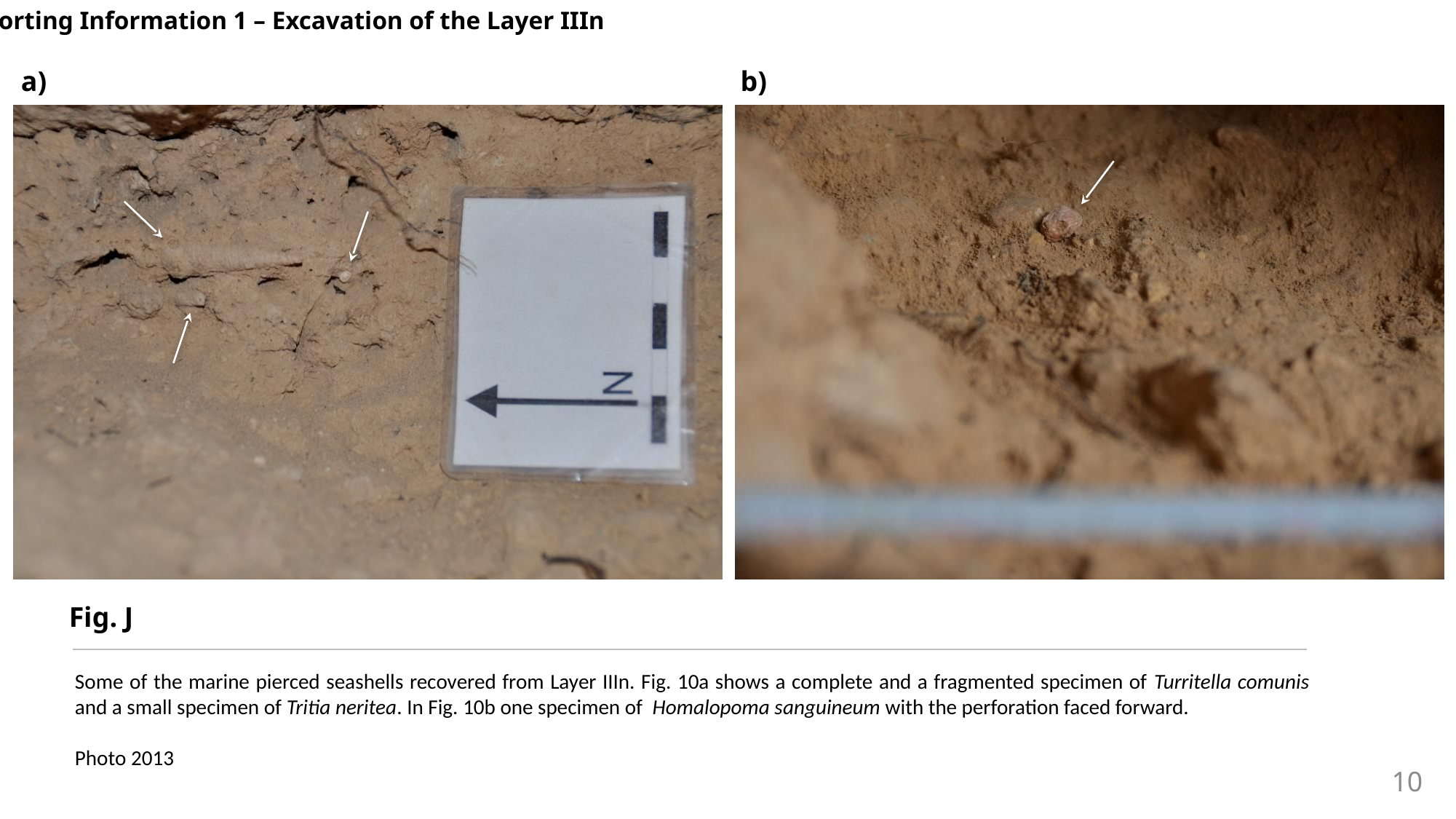

Supporting Information 1 – Excavation of the Layer IIIn
a)
b)
Fig. J
Some of the marine pierced seashells recovered from Layer IIIn. Fig. 10a shows a complete and a fragmented specimen of Turritella comunis and a small specimen of Tritia neritea. In Fig. 10b one specimen of Homalopoma sanguineum with the perforation faced forward.
Photo 2013
10

## Slide 11
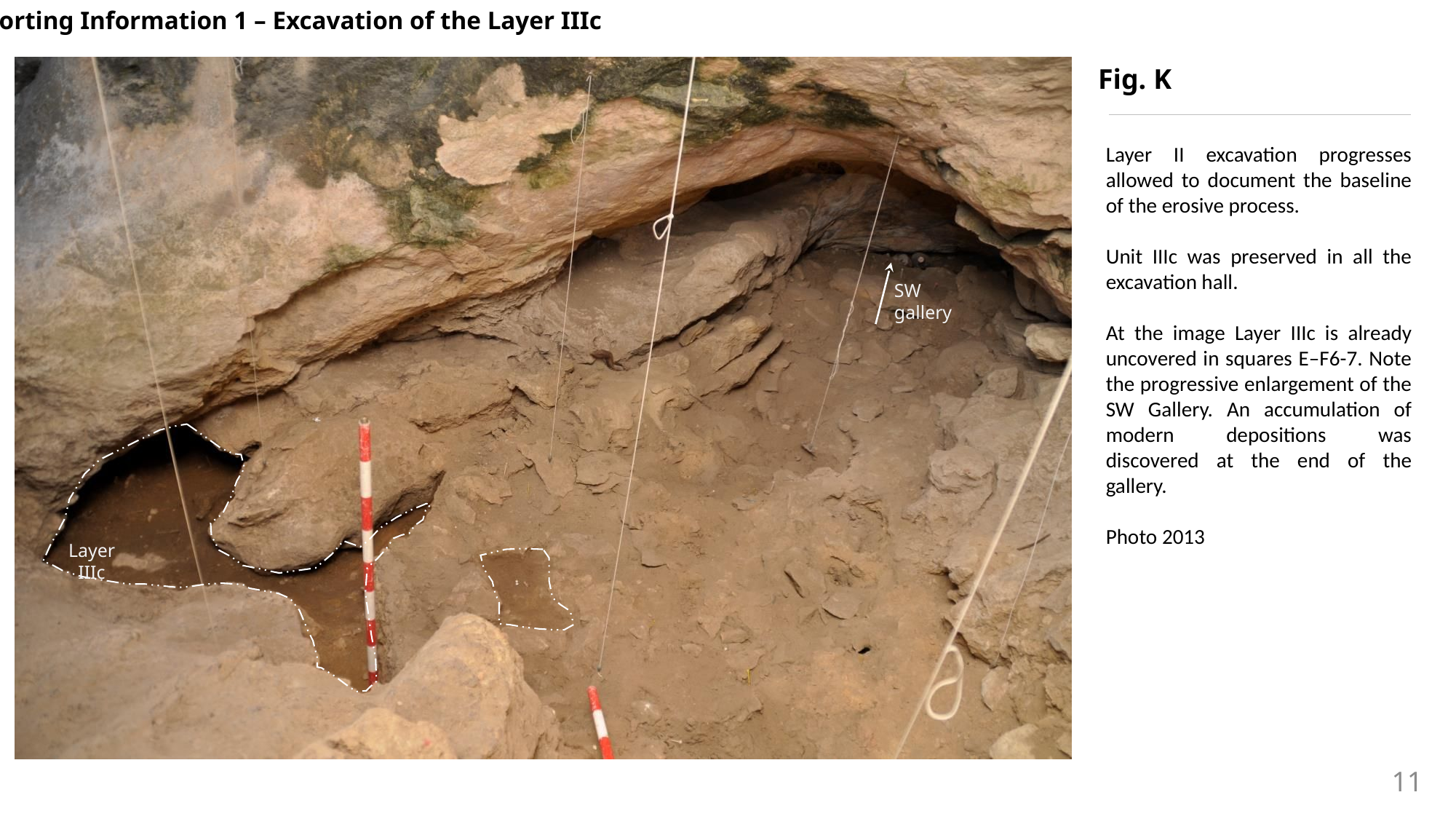

Supporting Information 1 – Excavation of the Layer IIIc
Fig. K
Layer II excavation progresses allowed to document the baseline of the erosive process.
Unit IIIc was preserved in all the excavation hall.
At the image Layer IIIc is already uncovered in squares E–F6-7. Note the progressive enlargement of the SW Gallery. An accumulation of modern depositions was discovered at the end of the gallery.
Photo 2013
SW
gallery
Layer IIIc
11

## Slide 12
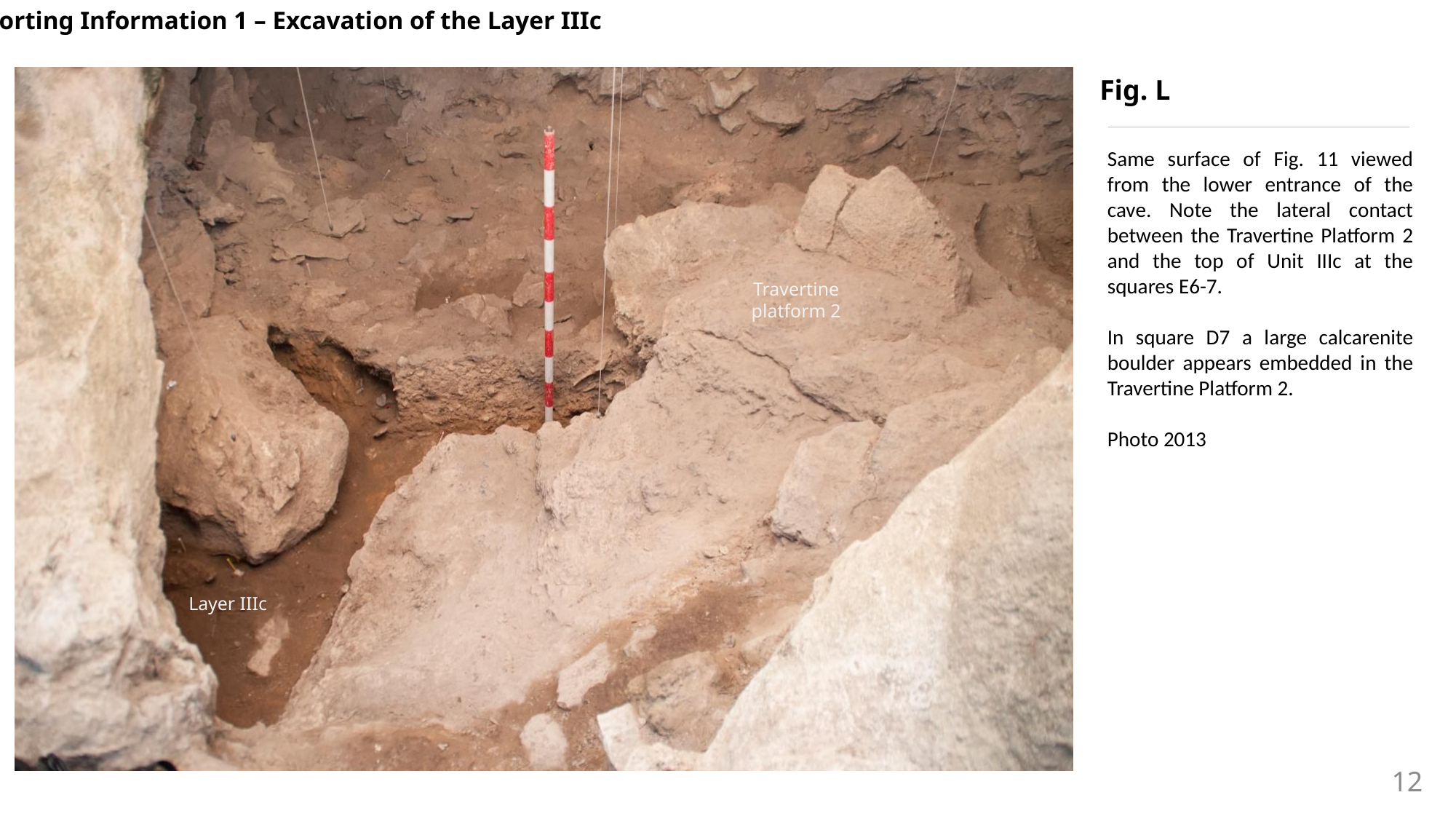

Supporting Information 1 – Excavation of the Layer IIIc
Fig. L
Same surface of Fig. 11 viewed from the lower entrance of the cave. Note the lateral contact between the Travertine Platform 2 and the top of Unit IIIc at the squares E6-7.
In square D7 a large calcarenite boulder appears embedded in the Travertine Platform 2.
Photo 2013
Travertine
platform 2
Layer IIIc
12

## Slide 13
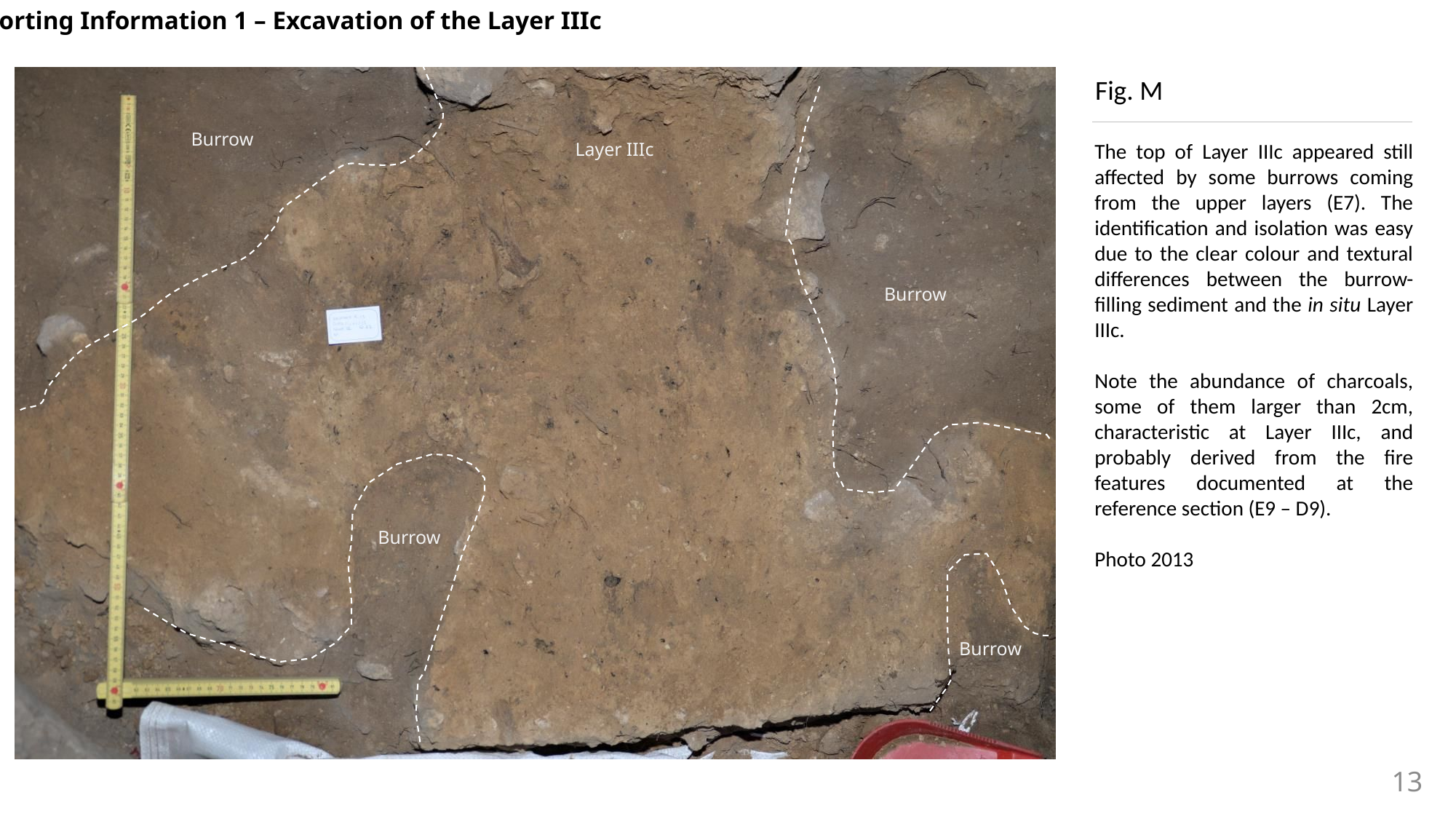

Supporting Information 1 – Excavation of the Layer IIIc
Fig. M
Burrow
Layer IIIc
The top of Layer IIIc appeared still affected by some burrows coming from the upper layers (E7). The identification and isolation was easy due to the clear colour and textural differences between the burrow-filling sediment and the in situ Layer IIIc.
Note the abundance of charcoals, some of them larger than 2cm, characteristic at Layer IIIc, and probably derived from the fire features documented at the reference section (E9 – D9).
Photo 2013
Burrow
Burrow
Burrow
13

## Slide 14
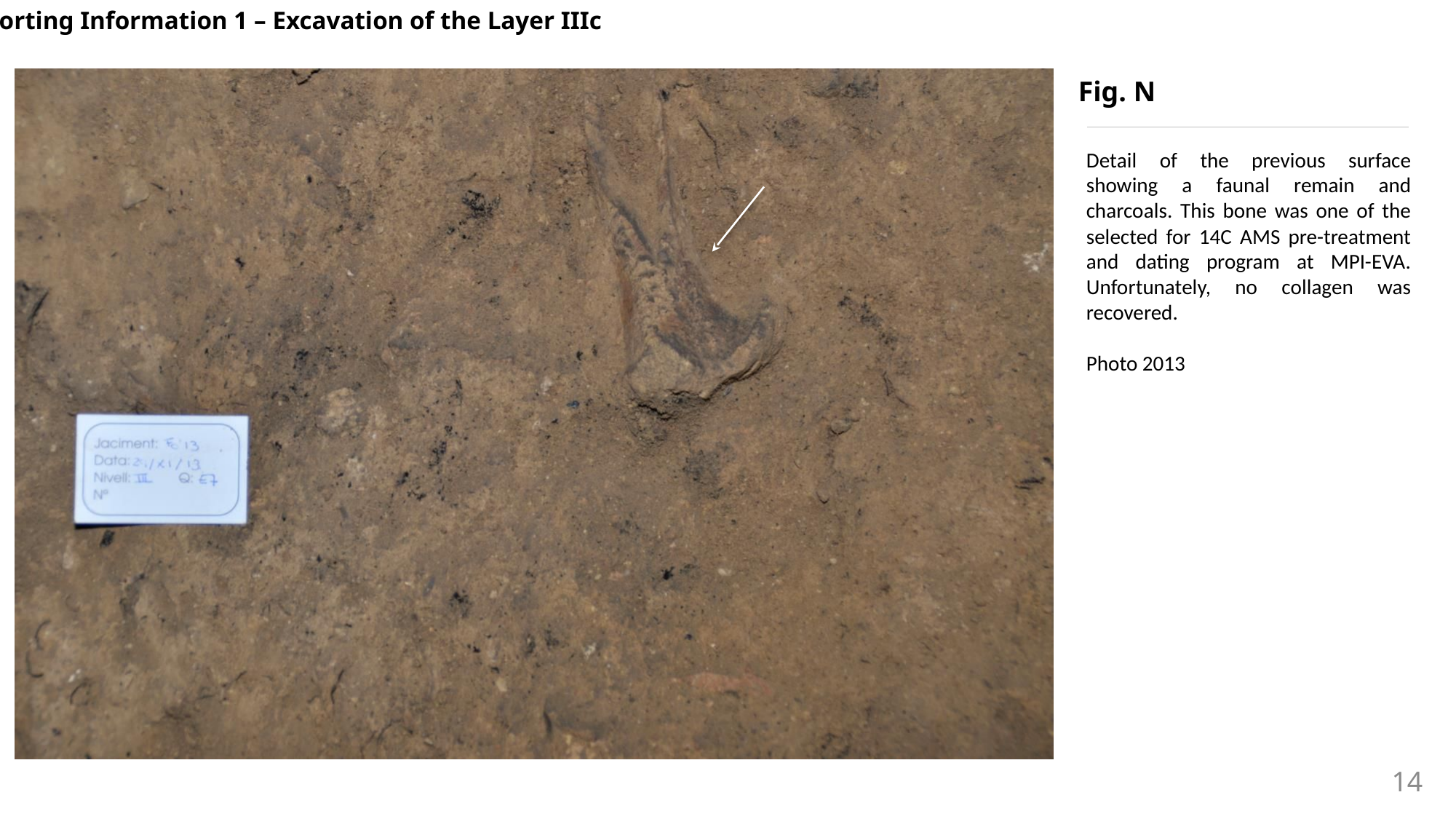

Supporting Information 1 – Excavation of the Layer IIIc
Fig. N
Detail of the previous surface showing a faunal remain and charcoals. This bone was one of the selected for 14C AMS pre-treatment and dating program at MPI-EVA. Unfortunately, no collagen was recovered.
Photo 2013
14

## Slide 15
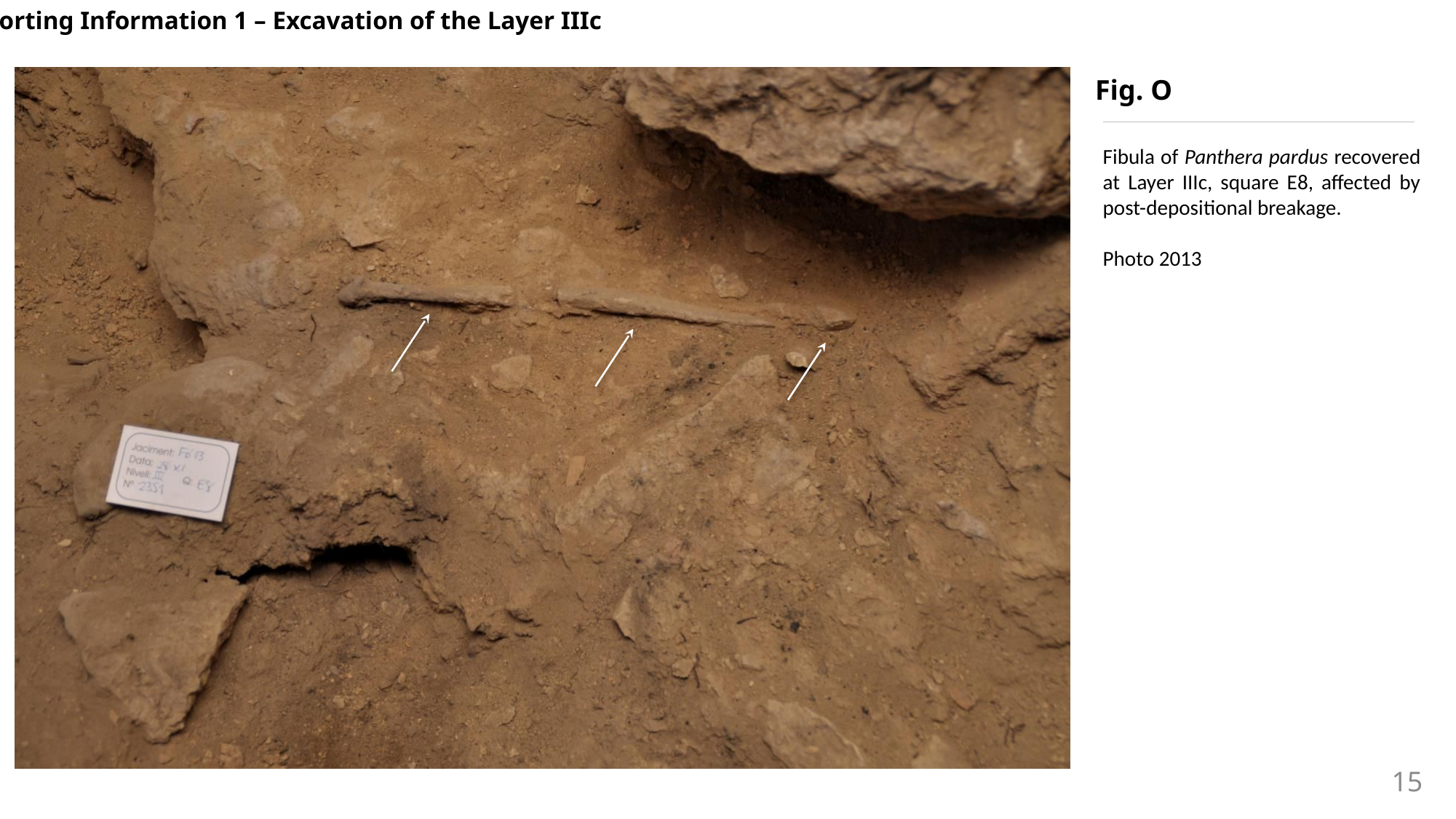

Supporting Information 1 – Excavation of the Layer IIIc
Fig. O
Fibula of Panthera pardus recovered at Layer IIIc, square E8, affected by post-depositional breakage.
Photo 2013
15

## Slide 16
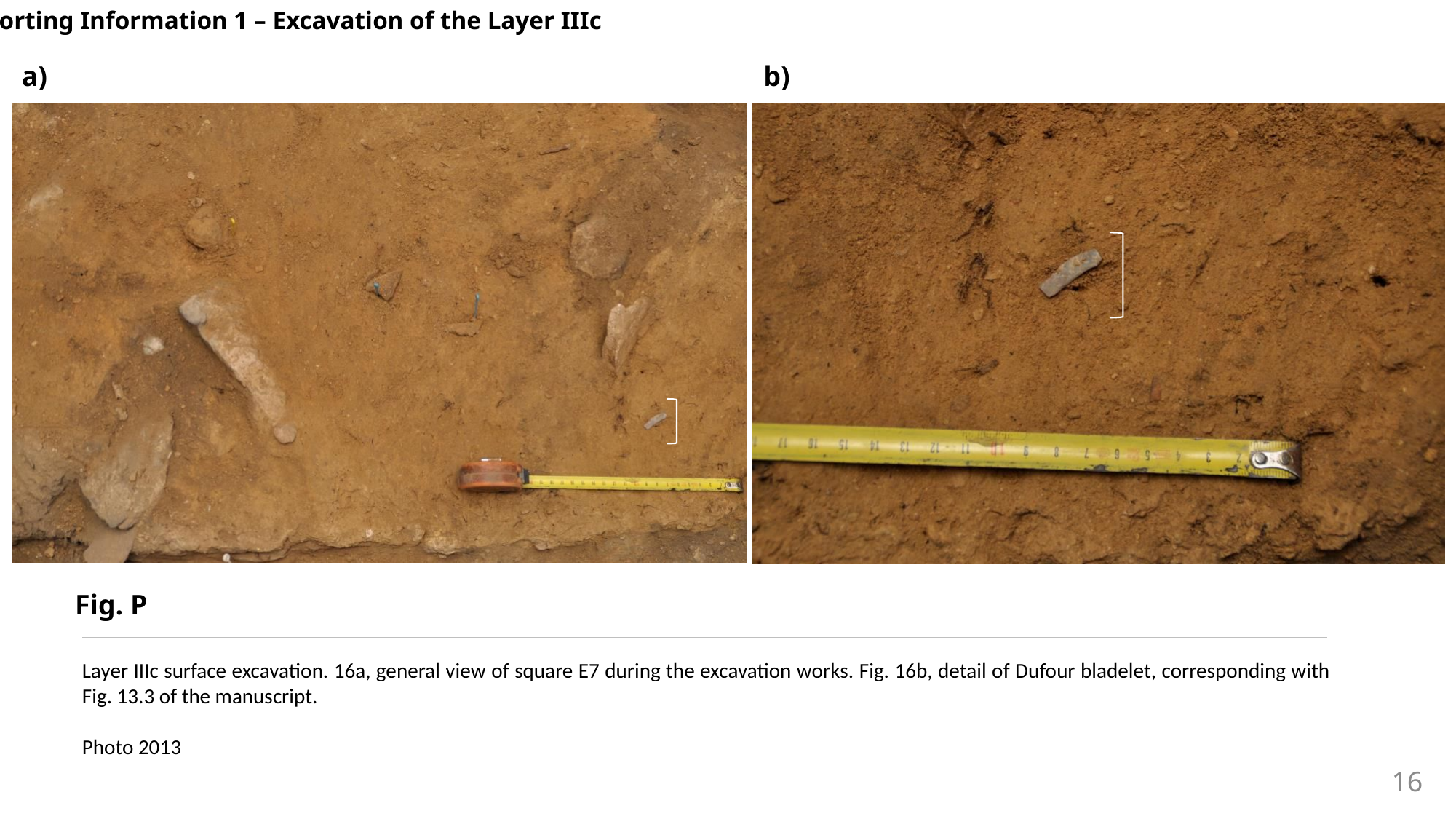

Supporting Information 1 – Excavation of the Layer IIIc
a)
b)
Fig. P
Layer IIIc surface excavation. 16a, general view of square E7 during the excavation works. Fig. 16b, detail of Dufour bladelet, corresponding with Fig. 13.3 of the manuscript.
Photo 2013
16

## Slide 17
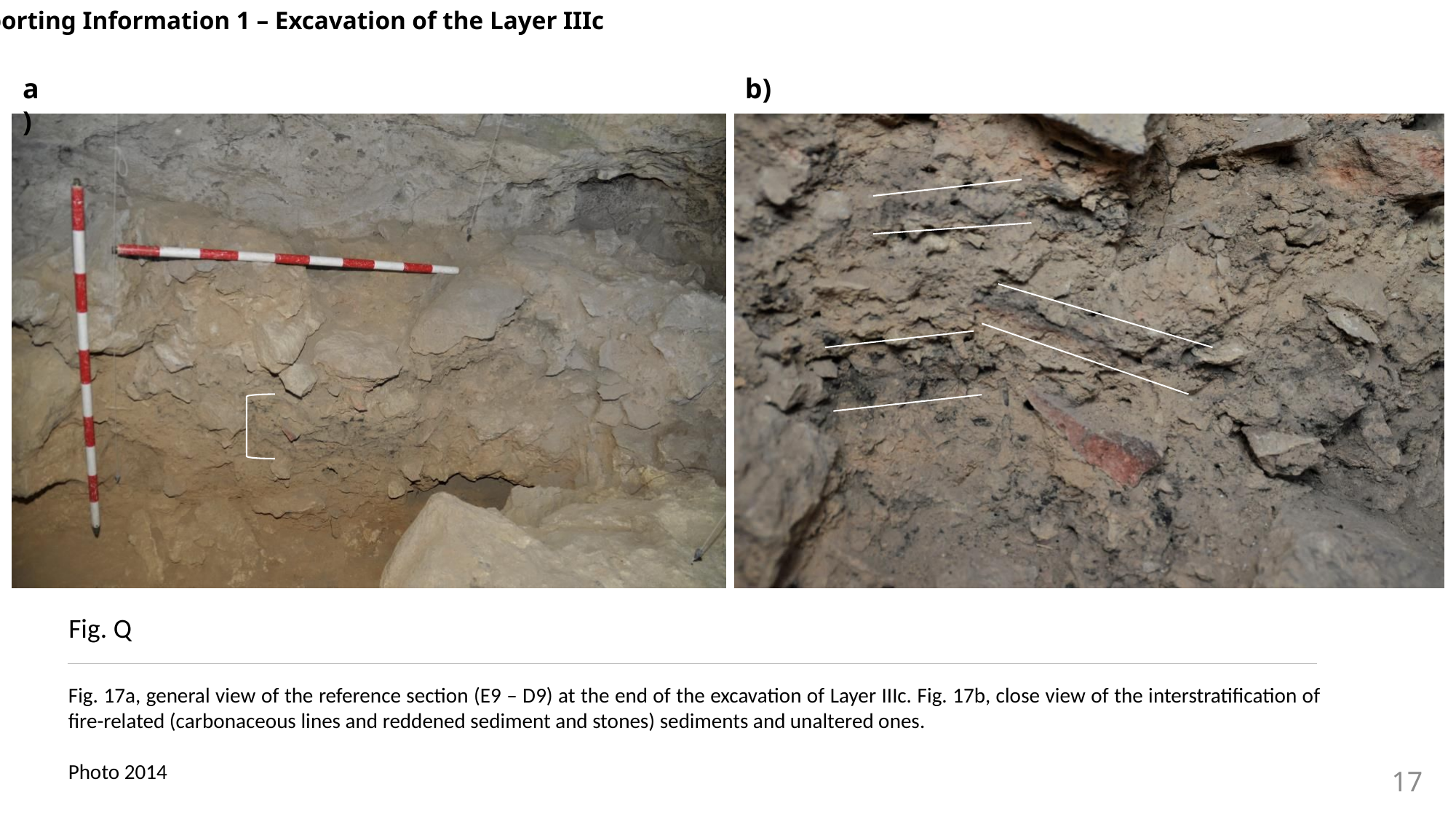

Supporting Information 1 – Excavation of the Layer IIIc
a)
b)
Fig. Q
Fig. 17a, general view of the reference section (E9 – D9) at the end of the excavation of Layer IIIc. Fig. 17b, close view of the interstratification of fire-related (carbonaceous lines and reddened sediment and stones) sediments and unaltered ones.
Photo 2014
17

## Slide 18
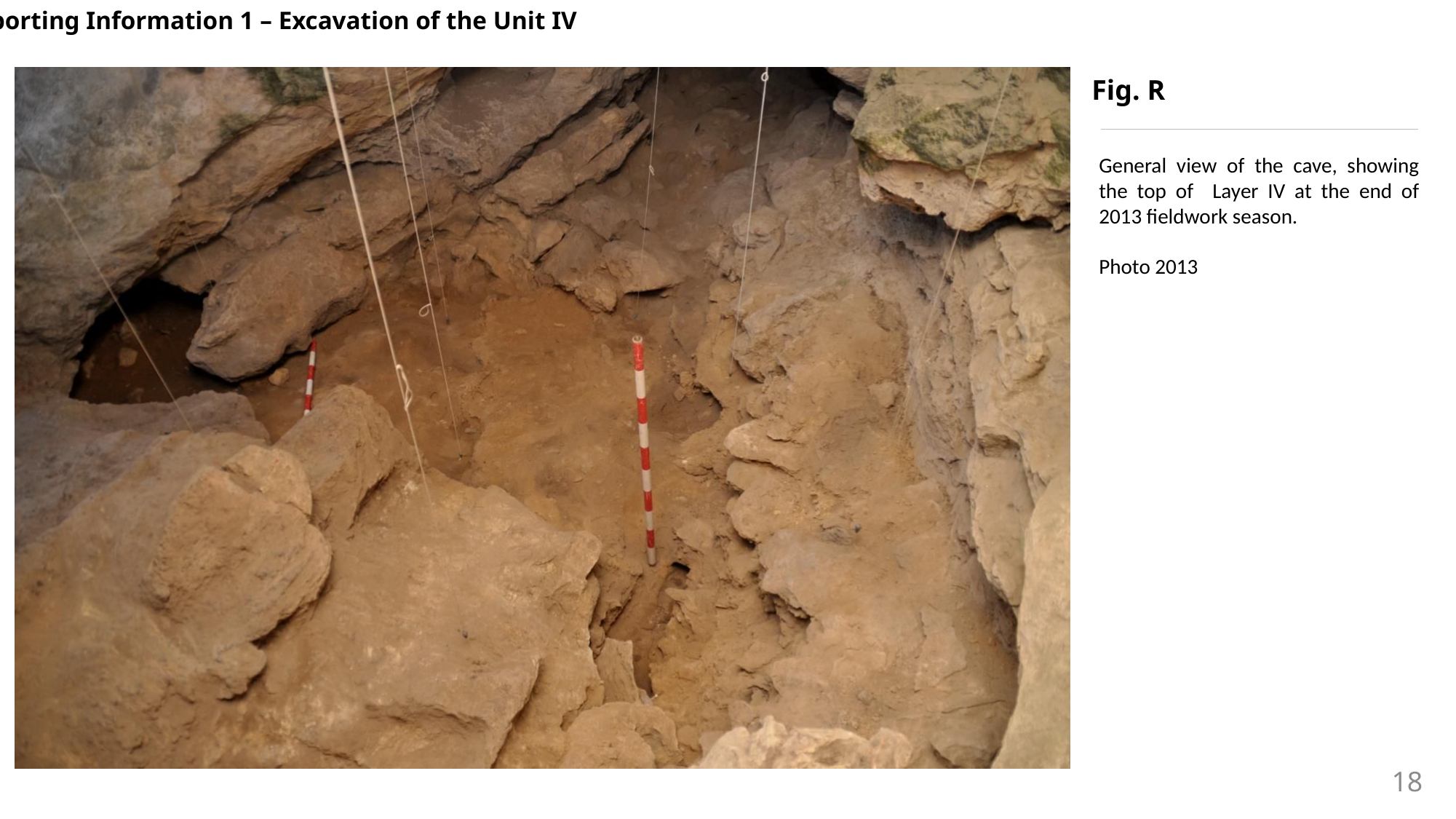

Supporting Information 1 – Excavation of the Unit IV
Fig. R
General view of the cave, showing the top of Layer IV at the end of 2013 fieldwork season.
Photo 2013
18

## Slide 19
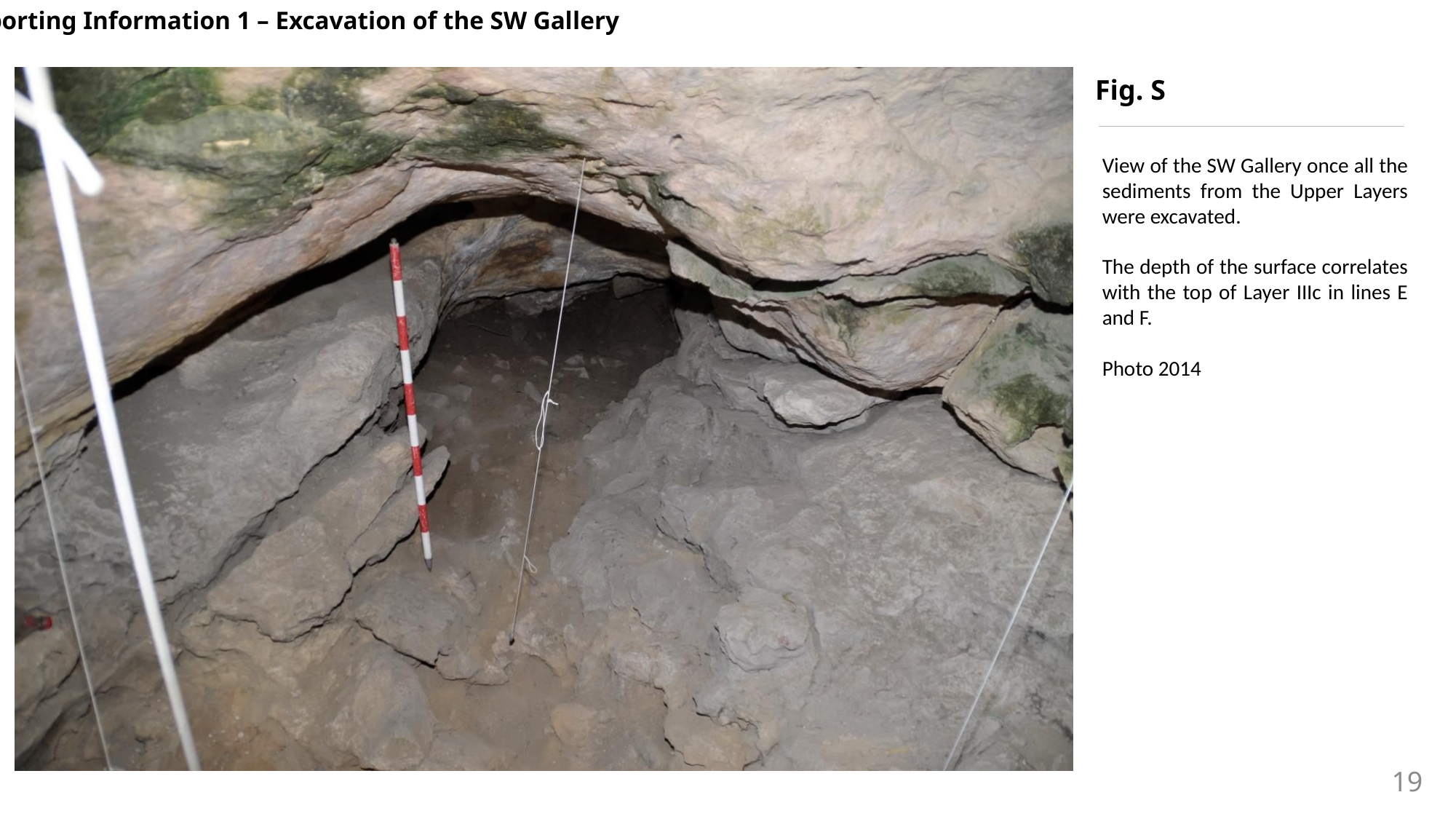

Supporting Information 1 – Excavation of the SW Gallery
Fig. S
View of the SW Gallery once all the sediments from the Upper Layers were excavated.
The depth of the surface correlates with the top of Layer IIIc in lines E and F.
Photo 2014
19

## Slide 20
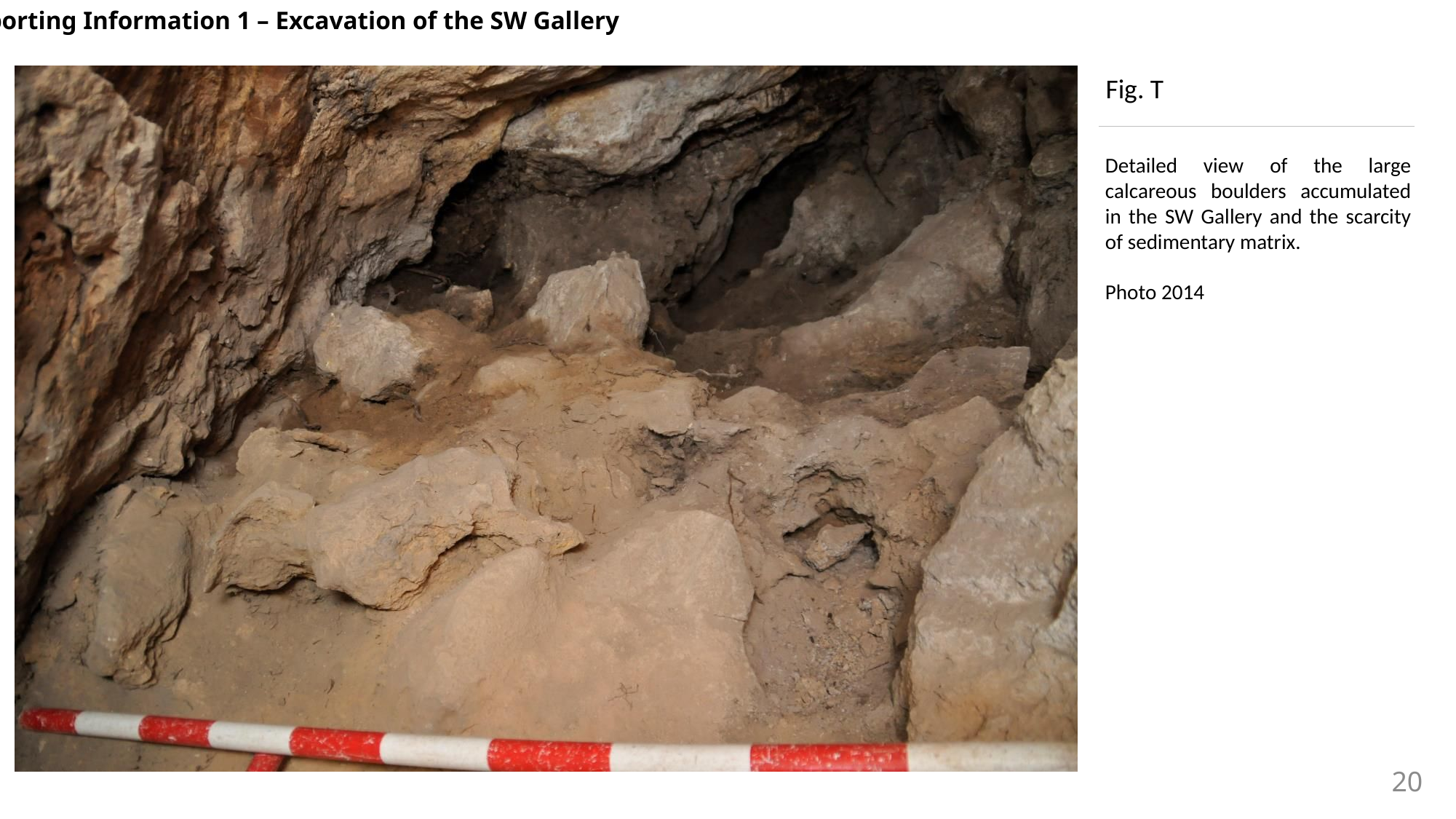

Supporting Information 1 – Excavation of the SW Gallery
Fig. T
Detailed view of the large calcareous boulders accumulated in the SW Gallery and the scarcity of sedimentary matrix.
Photo 2014
20

## Slide 21
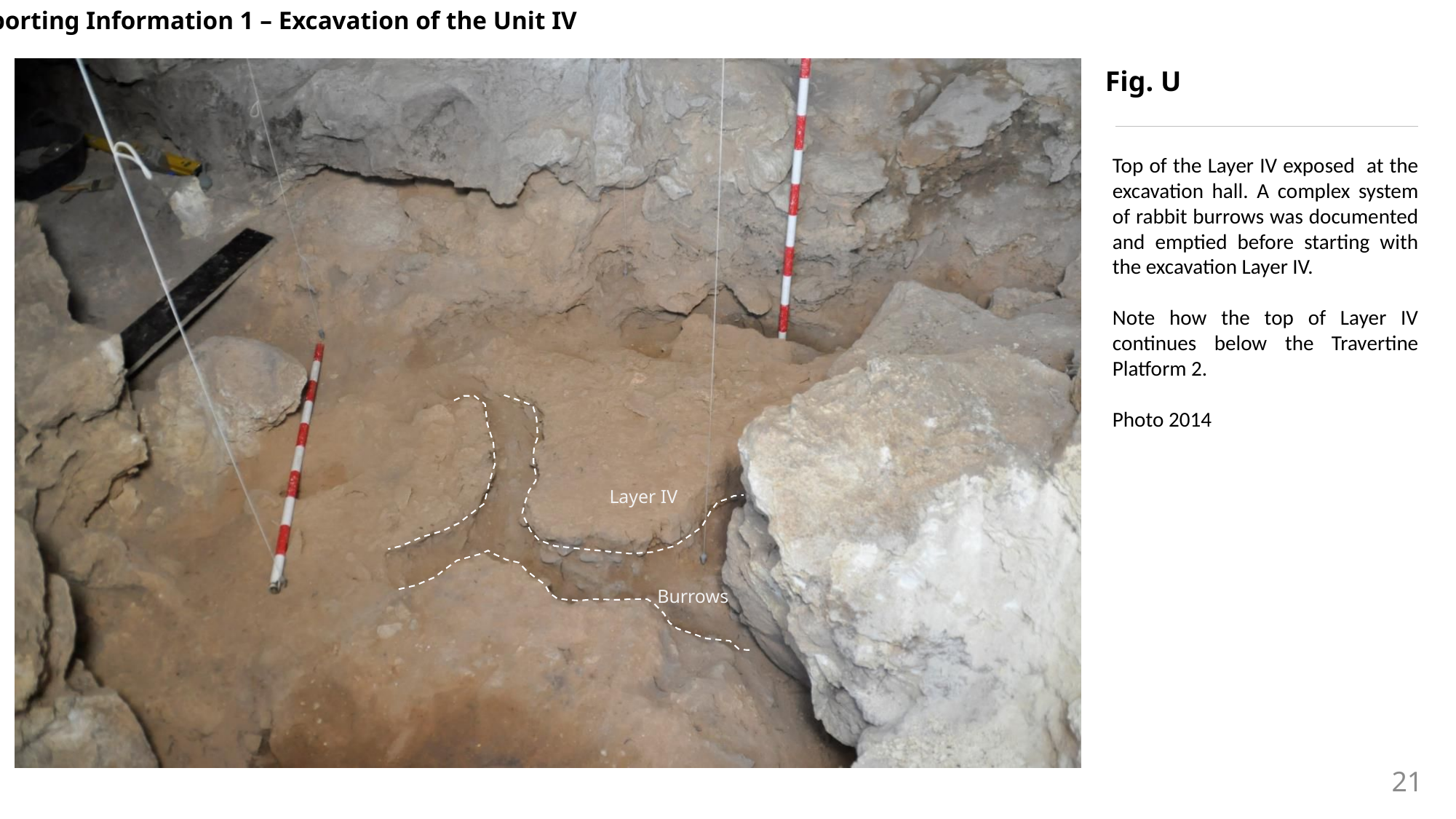

Supporting Information 1 – Excavation of the Unit IV
Fig. U
Top of the Layer IV exposed at the excavation hall. A complex system of rabbit burrows was documented and emptied before starting with the excavation Layer IV.
Note how the top of Layer IV continues below the Travertine Platform 2.
Photo 2014
Layer IV
Burrows
21

## Slide 22
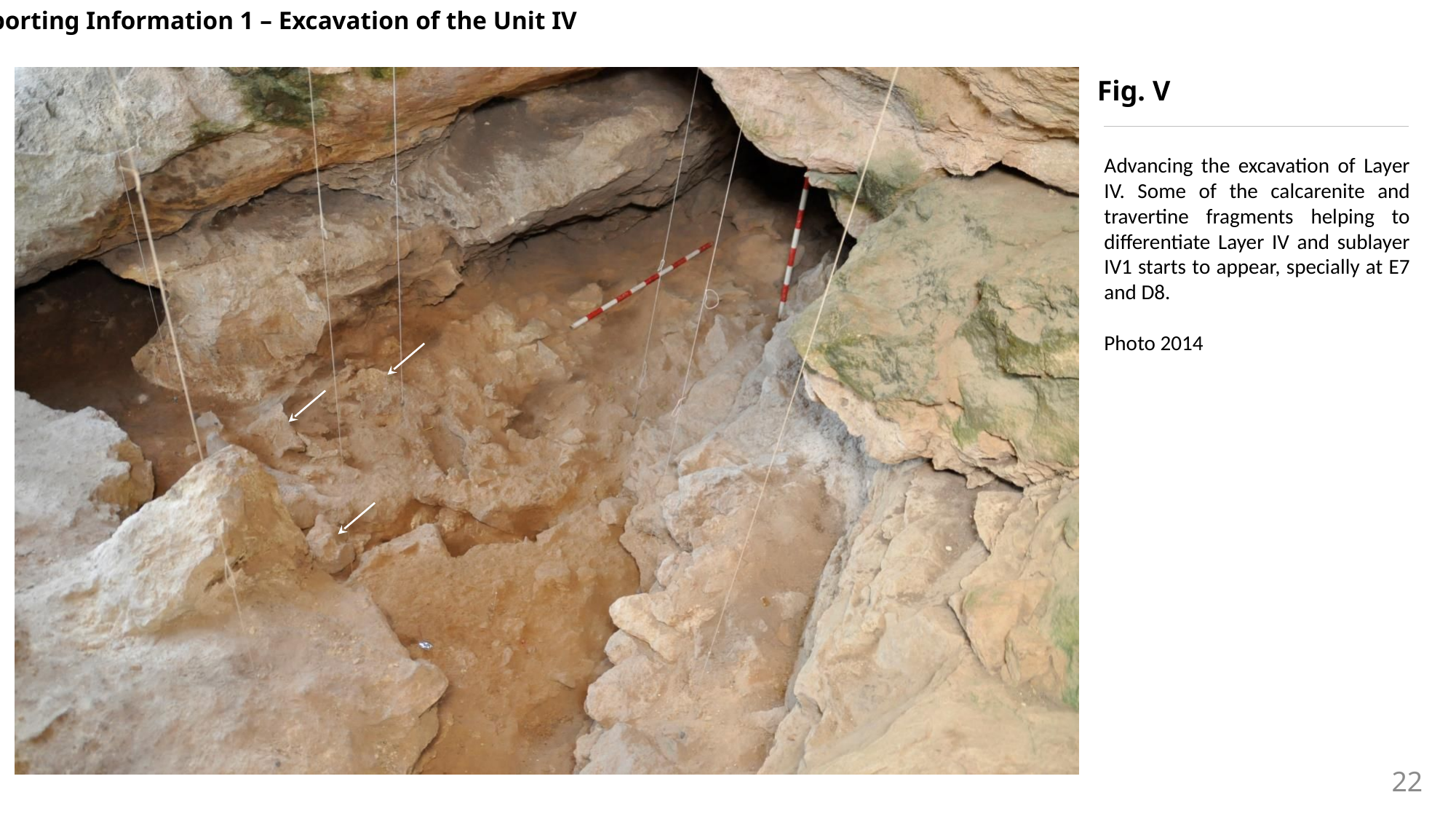

Supporting Information 1 – Excavation of the Unit IV
Fig. V
Advancing the excavation of Layer IV. Some of the calcarenite and travertine fragments helping to differentiate Layer IV and sublayer IV1 starts to appear, specially at E7 and D8.
Photo 2014
22

## Slide 23
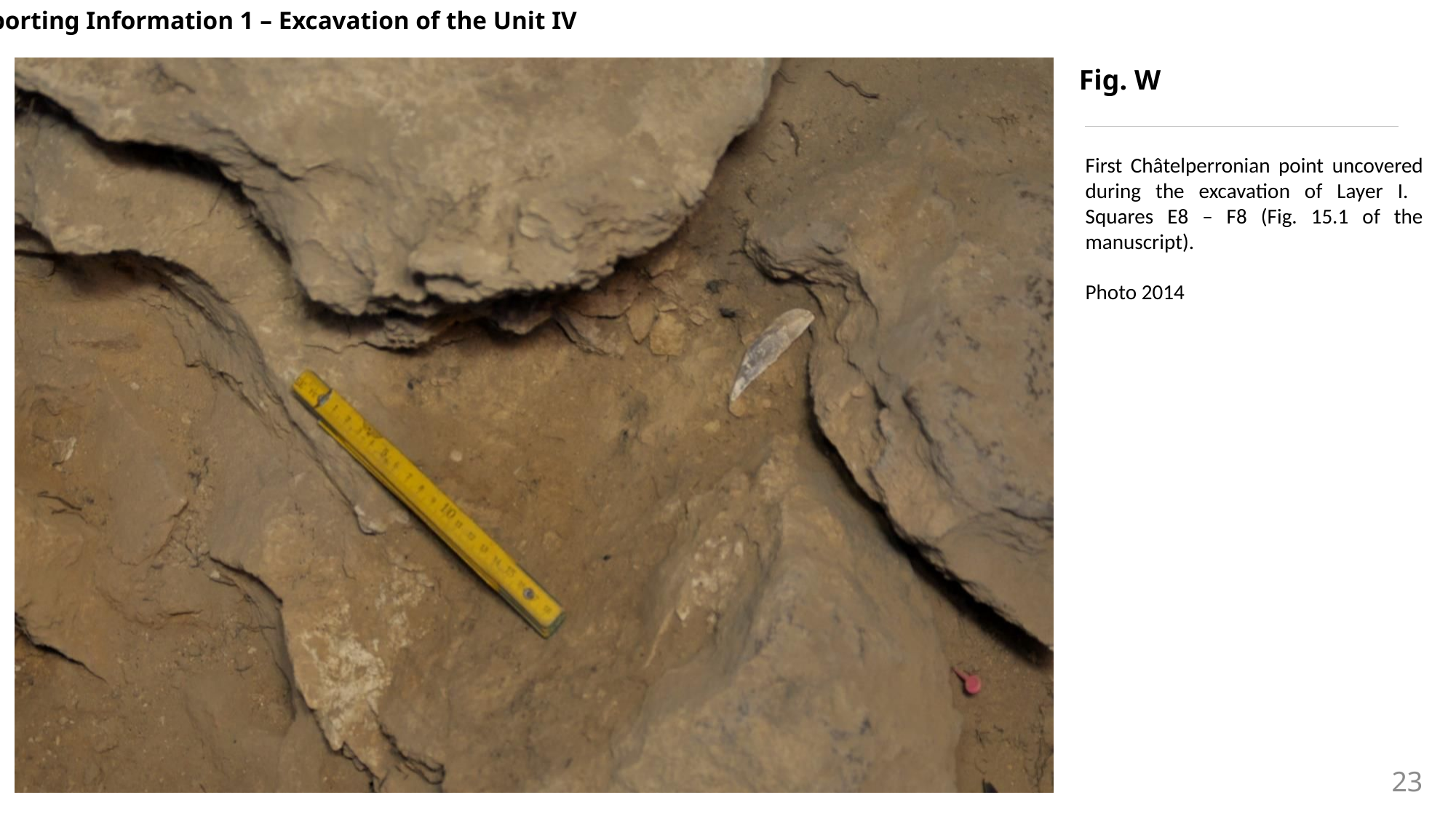

Supporting Information 1 – Excavation of the Unit IV
Fig. W
First Châtelperronian point uncovered during the excavation of Layer I. Squares E8 – F8 (Fig. 15.1 of the manuscript).
Photo 2014
23

## Slide 24
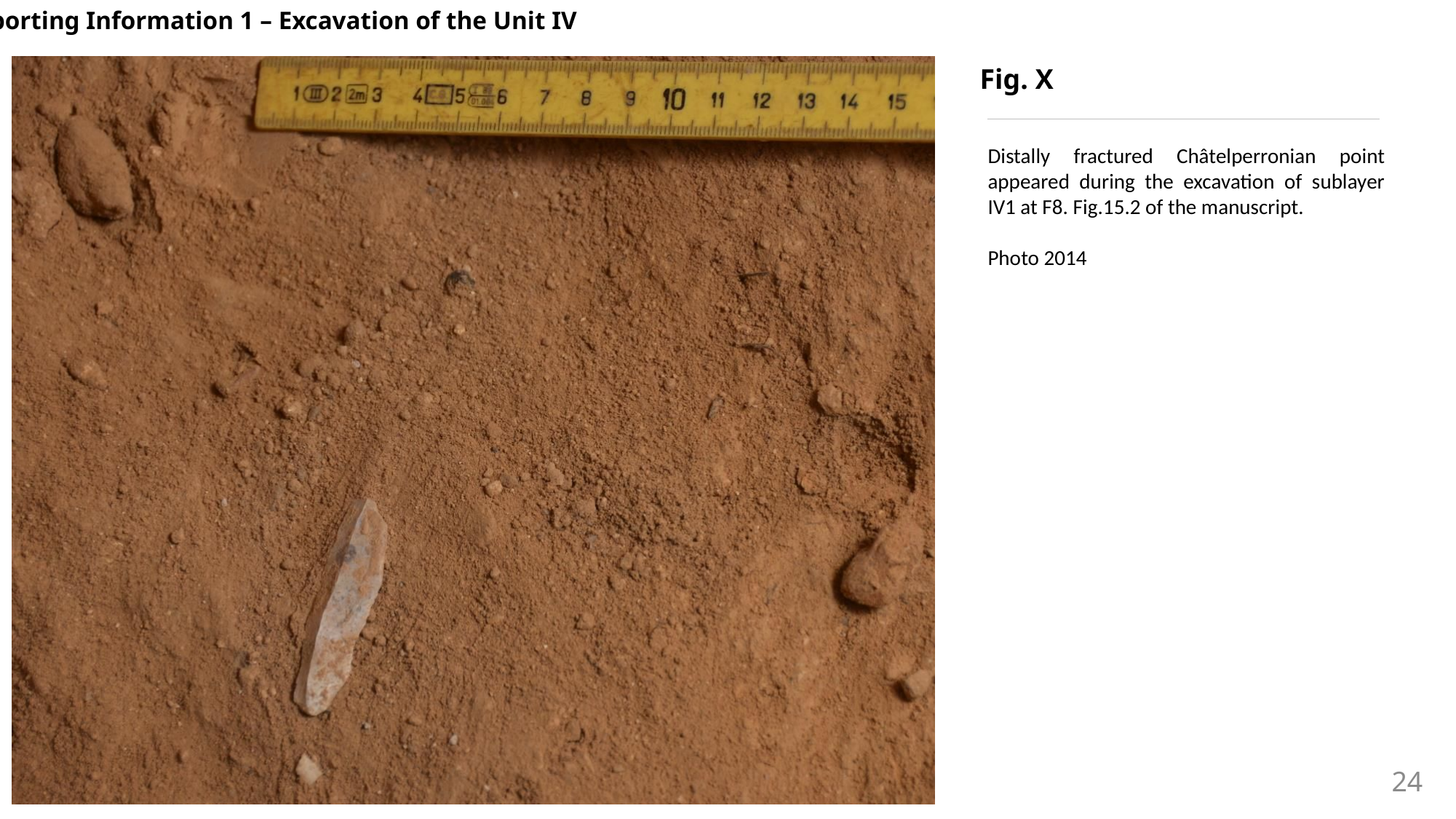

Supporting Information 1 – Excavation of the Unit IV
Fig. X
Distally fractured Châtelperronian point appeared during the excavation of sublayer IV1 at F8. Fig.15.2 of the manuscript.
Photo 2014
24

## Slide 25
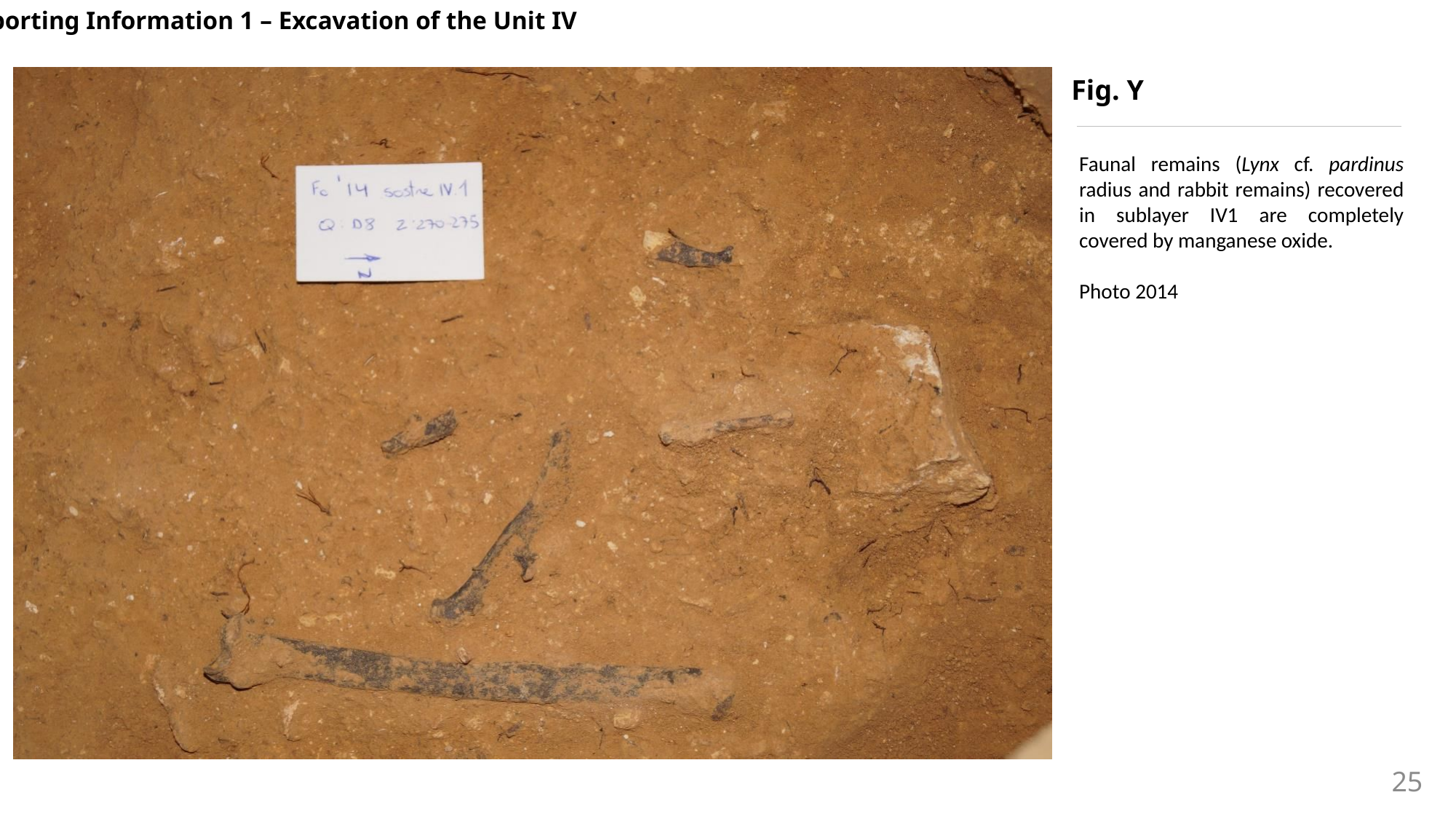

Supporting Information 1 – Excavation of the Unit IV
Fig. Y
Faunal remains (Lynx cf. pardinus radius and rabbit remains) recovered in sublayer IV1 are completely covered by manganese oxide.
Photo 2014
25

## Slide 26
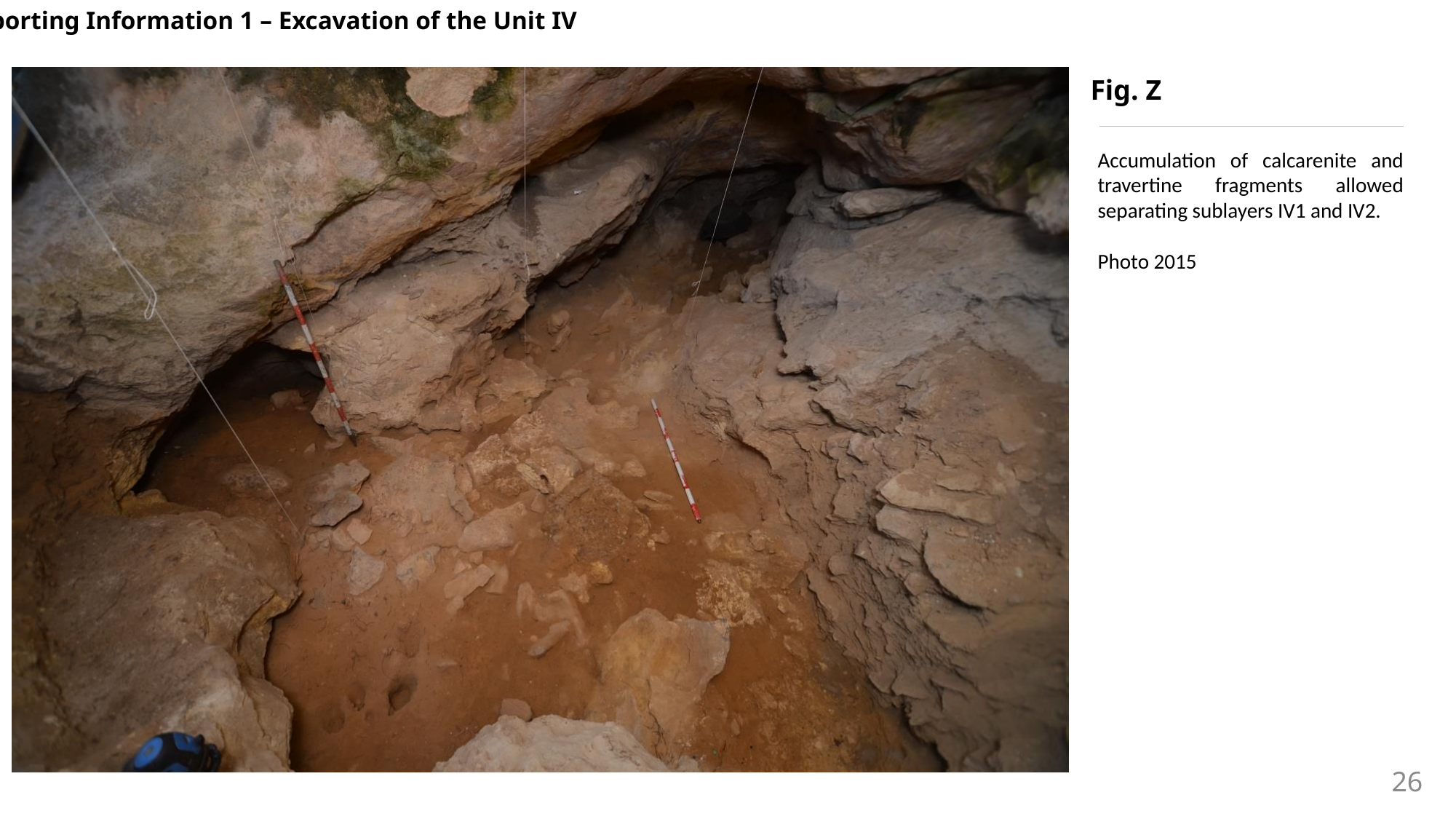

Supporting Information 1 – Excavation of the Unit IV
Fig. Z
Accumulation of calcarenite and travertine fragments allowed separating sublayers IV1 and IV2.
Photo 2015
26

## Slide 27
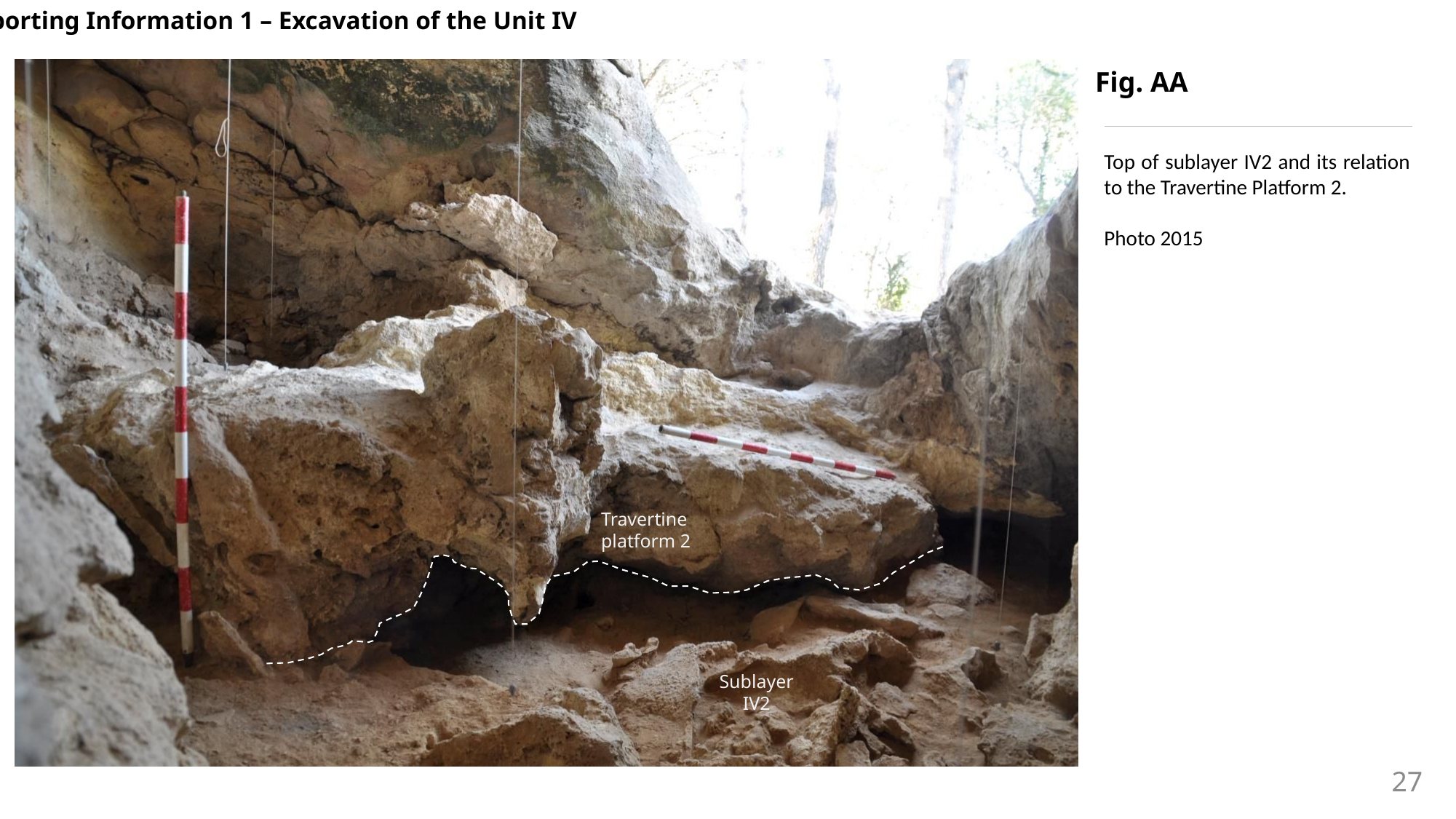

Supporting Information 1 – Excavation of the Unit IV
Fig. AA
Top of sublayer IV2 and its relation to the Travertine Platform 2.
Photo 2015
Travertine
platform 2
Sublayer
IV2
27

## Slide 28
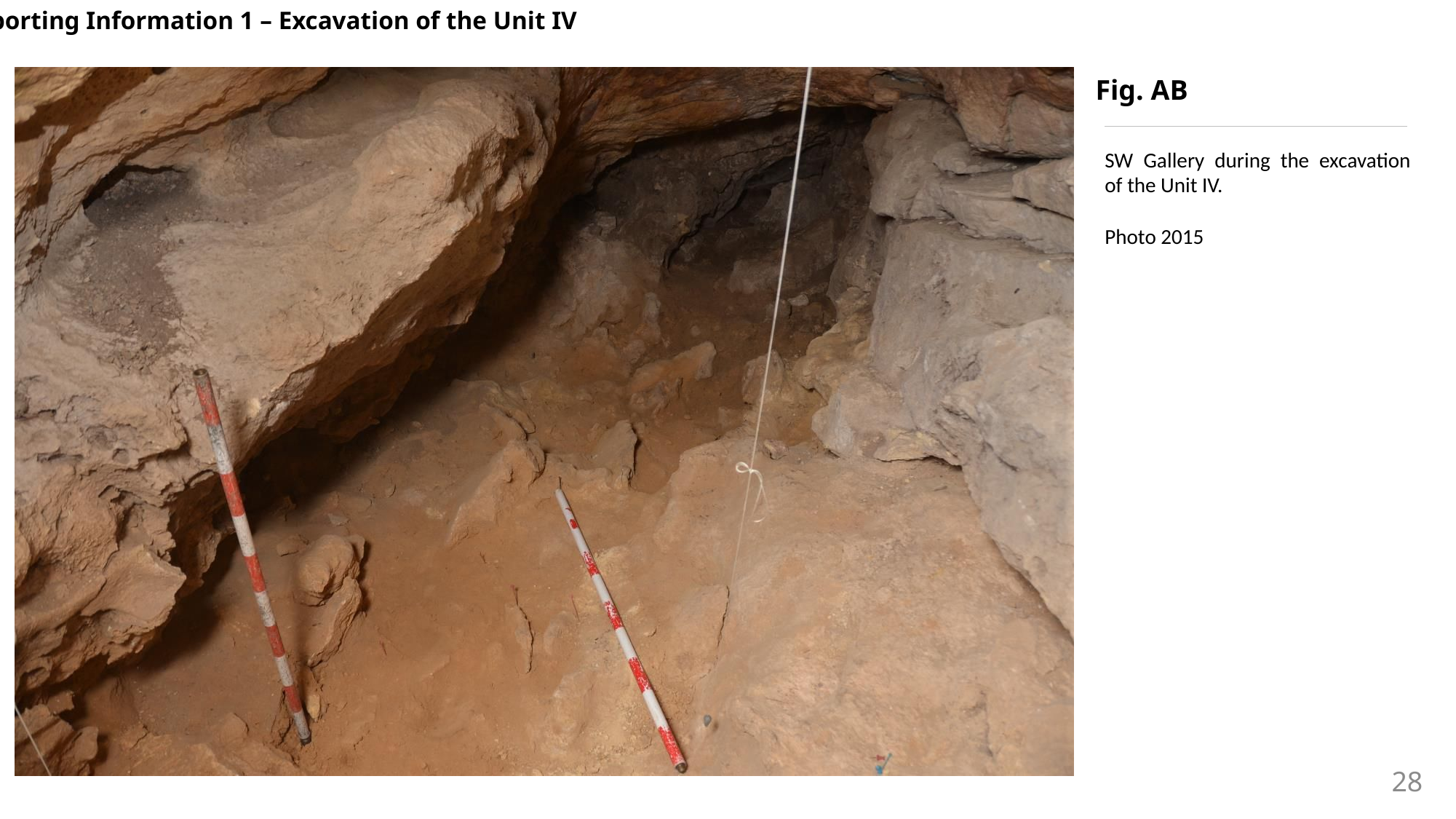

Supporting Information 1 – Excavation of the Unit IV
Fig. AB
SW Gallery during the excavation of the Unit IV.
Photo 2015
28

## Slide 29
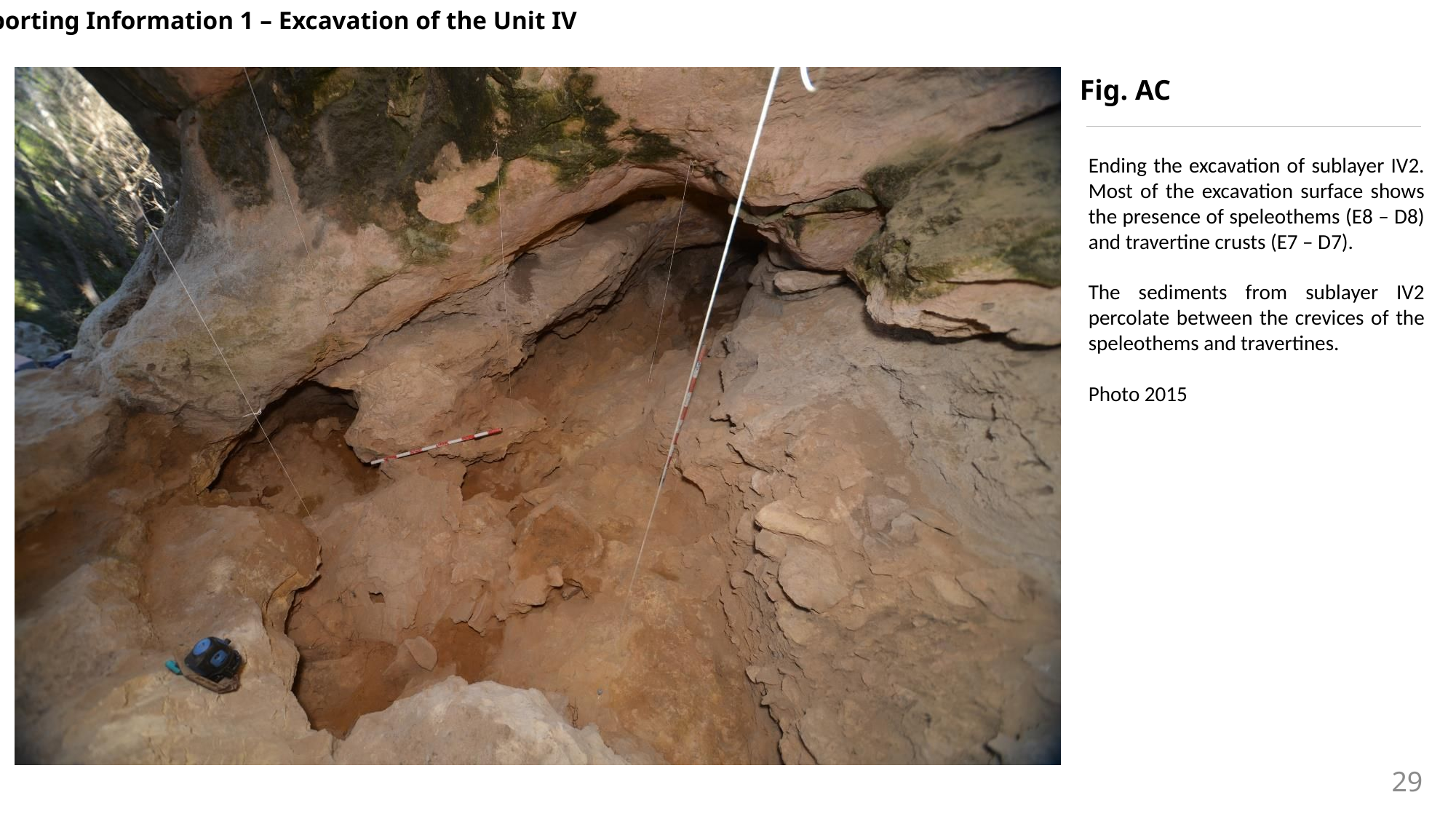

Supporting Information 1 – Excavation of the Unit IV
Fig. AC
Ending the excavation of sublayer IV2. Most of the excavation surface shows the presence of speleothems (E8 – D8) and travertine crusts (E7 – D7).
The sediments from sublayer IV2 percolate between the crevices of the speleothems and travertines.
Photo 2015
29

## Slide 30
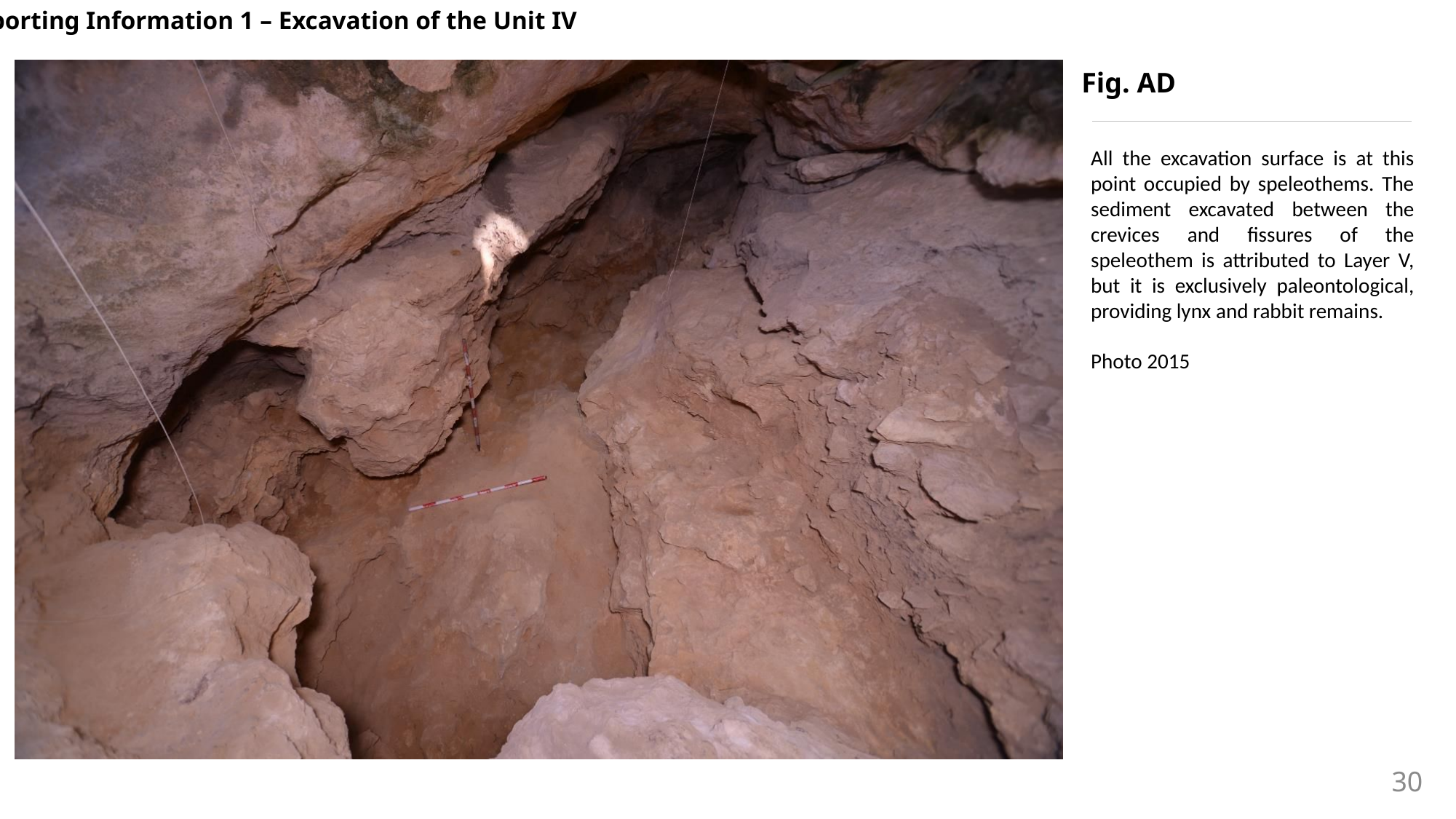

Supporting Information 1 – Excavation of the Unit IV
Fig. AD
All the excavation surface is at this point occupied by speleothems. The sediment excavated between the crevices and fissures of the speleothem is attributed to Layer V, but it is exclusively paleontological, providing lynx and rabbit remains.
Photo 2015
30

## Slide 31
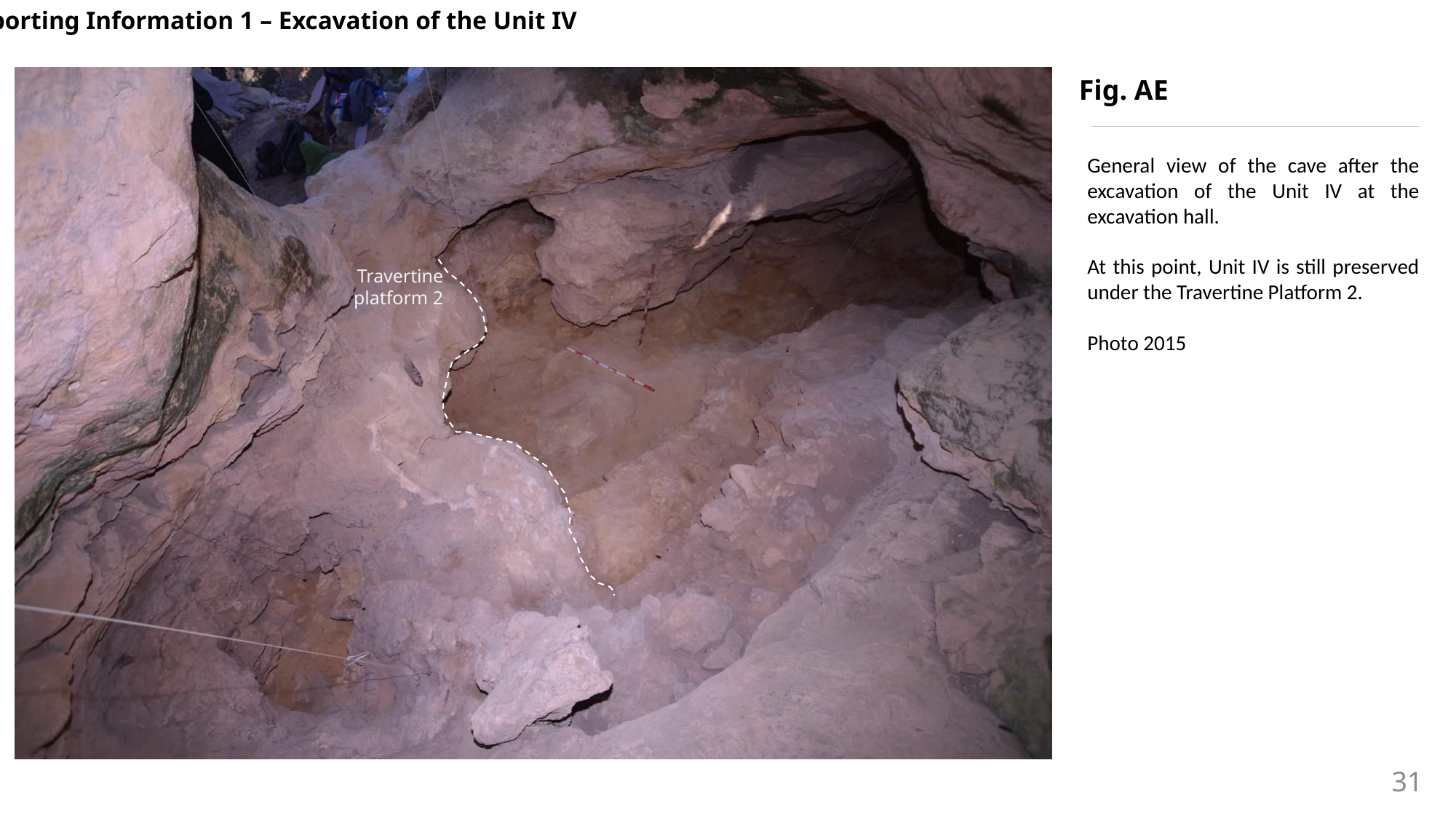

Supporting Information 1 – Excavation of the Unit IV
Fig. AE
General view of the cave after the excavation of the Unit IV at the excavation hall.
At this point, Unit IV is still preserved under the Travertine Platform 2.
Photo 2015
Travertine
platform 2
31

## Slide 32
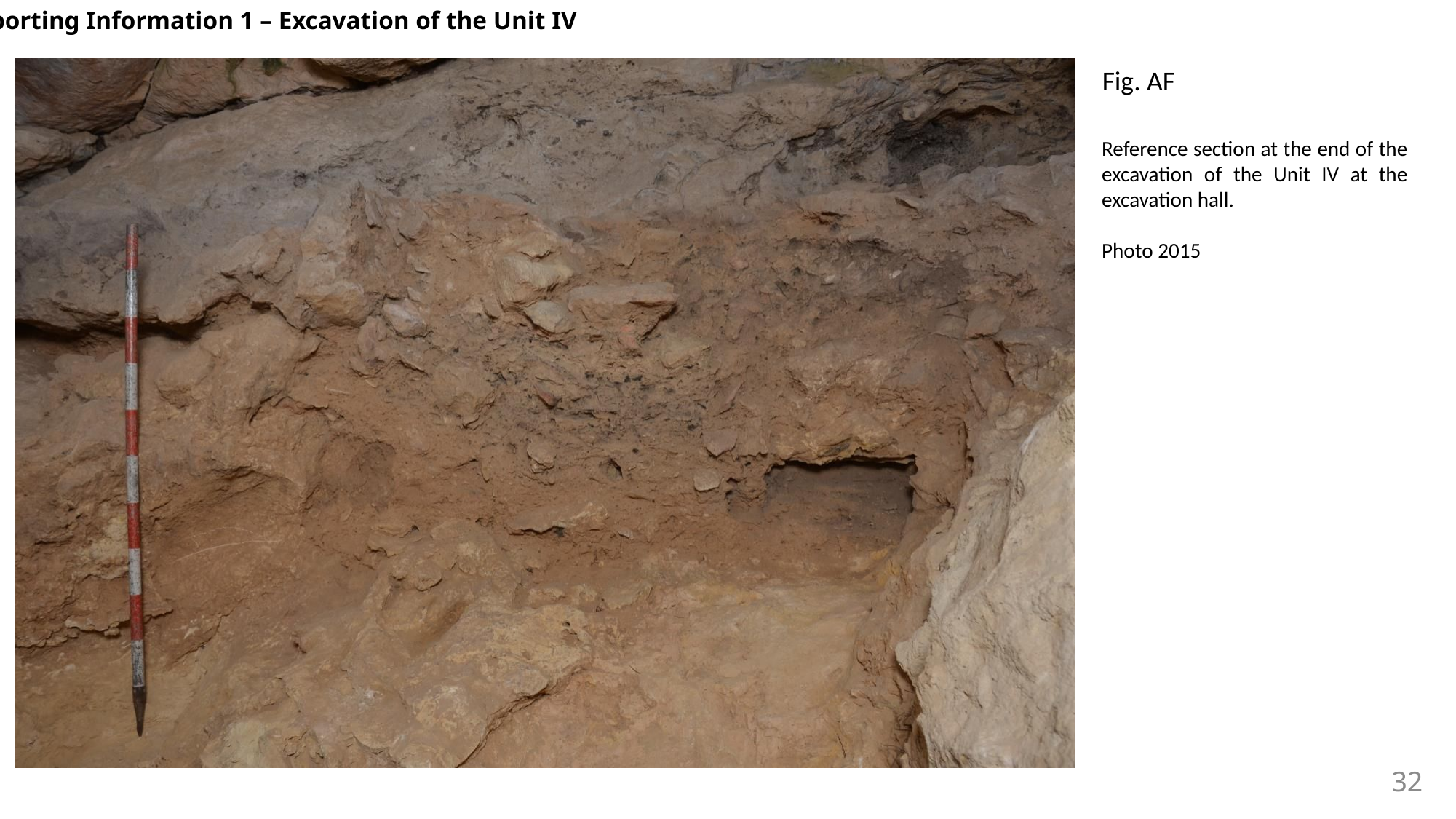

Supporting Information 1 – Excavation of the Unit IV
Fig. AF
Reference section at the end of the excavation of the Unit IV at the excavation hall.
Photo 2015
32

## Slide 33
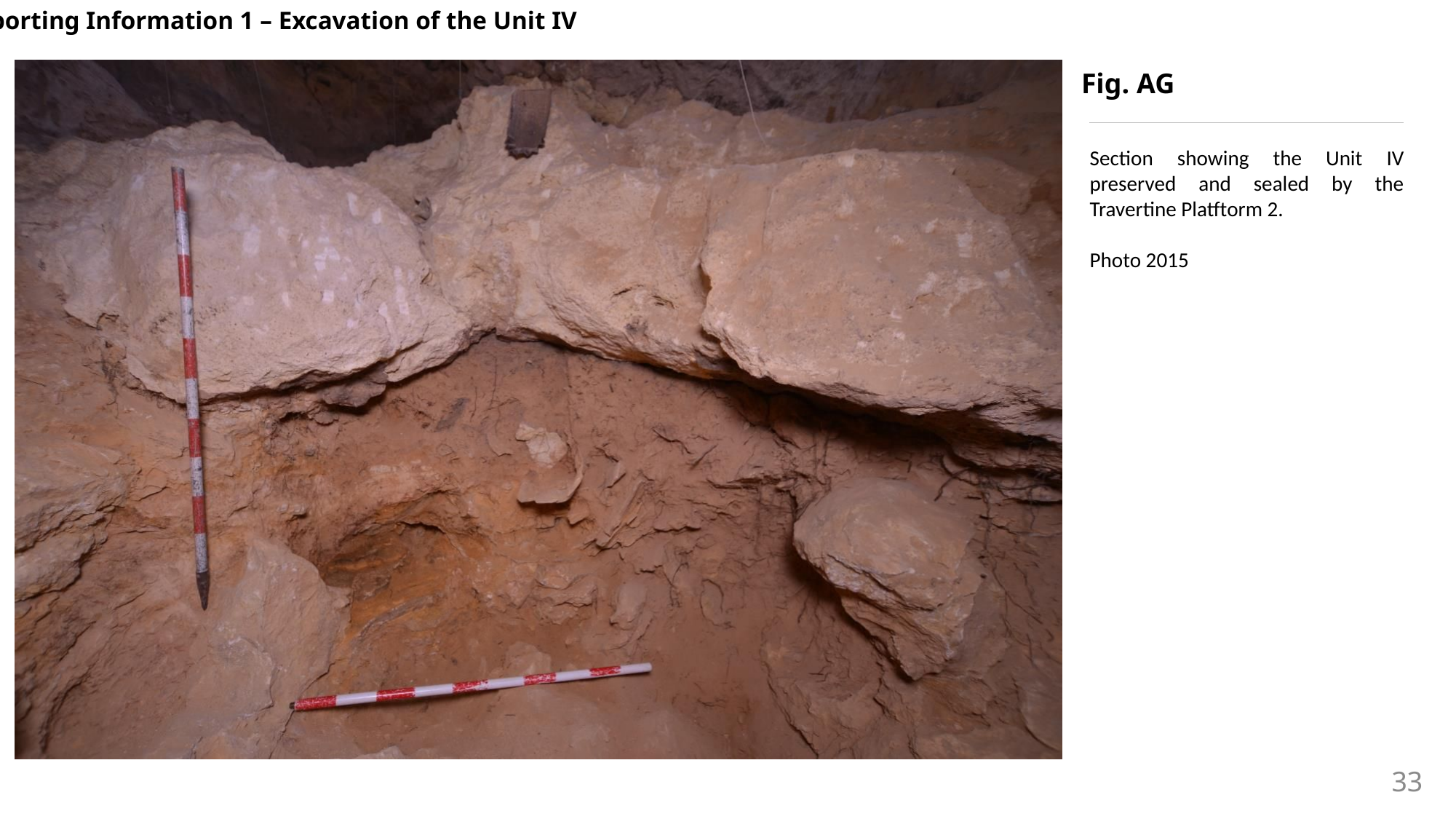

Supporting Information 1 – Excavation of the Unit IV
Fig. AG
Section showing the Unit IV preserved and sealed by the Travertine Platftorm 2.
Photo 2015
33

## Slide 34
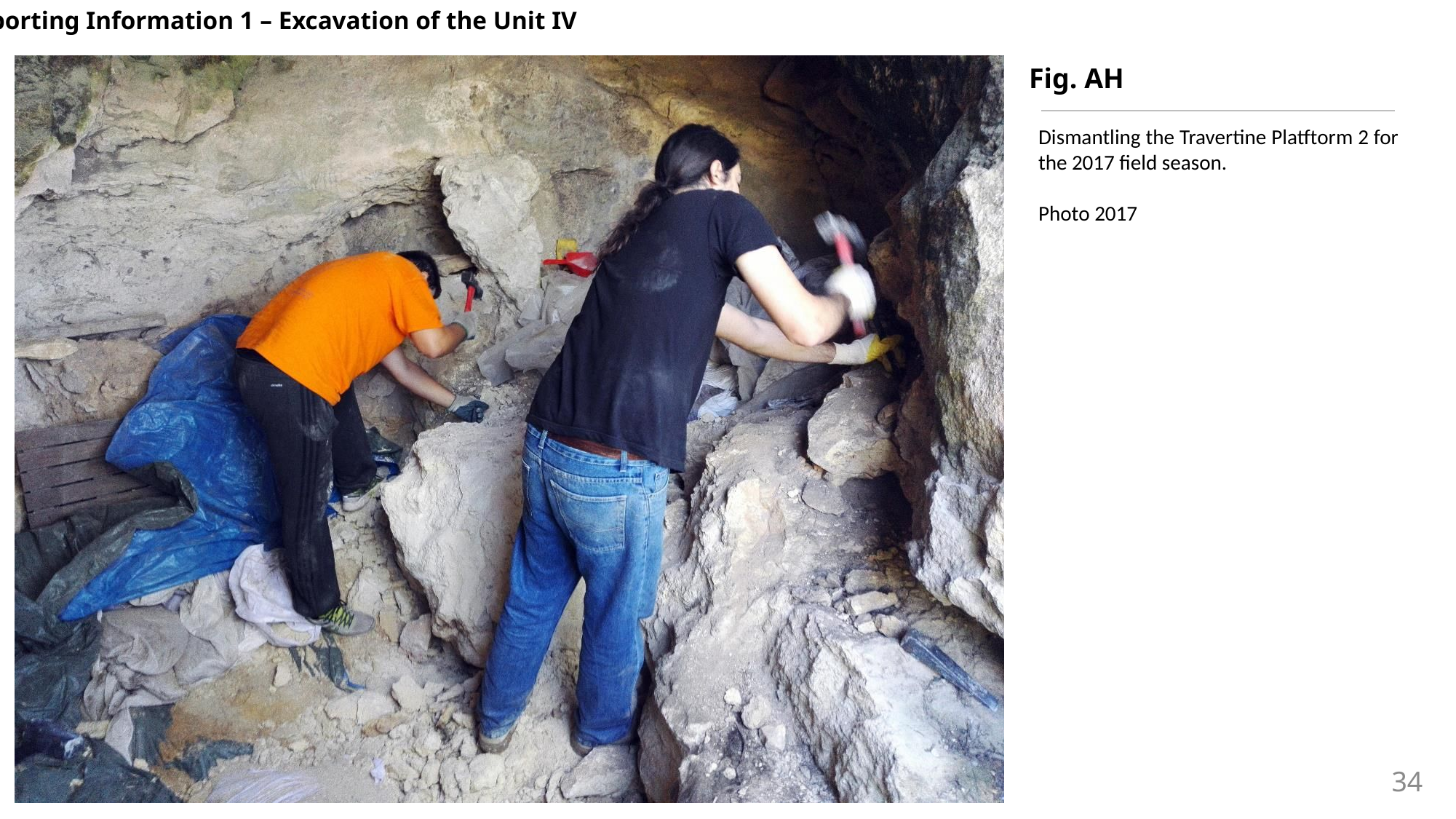

Supporting Information 1 – Excavation of the Unit IV
Fig. AH
Dismantling the Travertine Platftorm 2 for the 2017 field season.
Photo 2017
34

## Slide 35
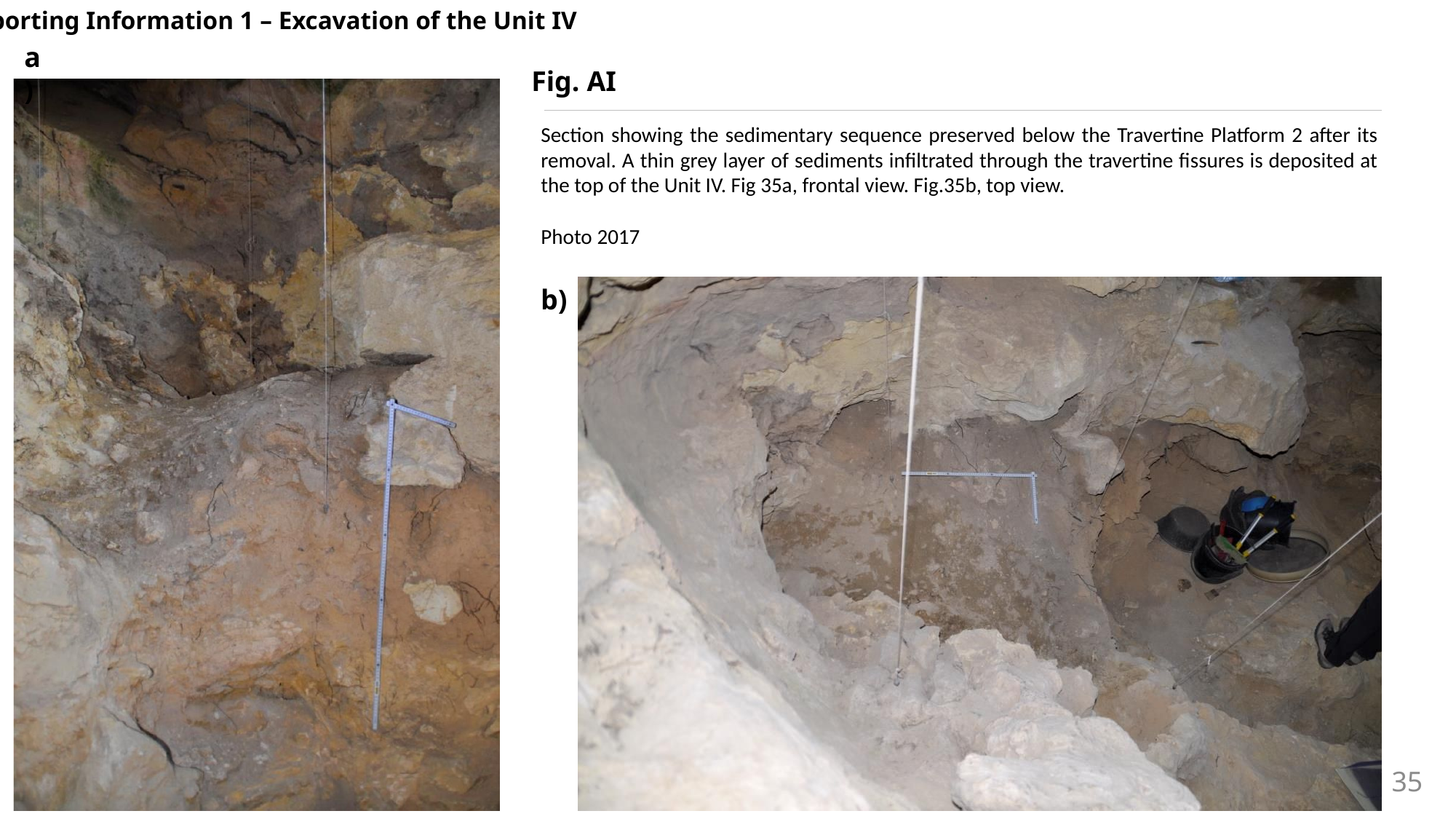

Supporting Information 1 – Excavation of the Unit IV
a)
Fig. AI
Section showing the sedimentary sequence preserved below the Travertine Platform 2 after its removal. A thin grey layer of sediments infiltrated through the travertine fissures is deposited at the top of the Unit IV. Fig 35a, frontal view. Fig.35b, top view.
Photo 2017
b)
35

## Slide 36
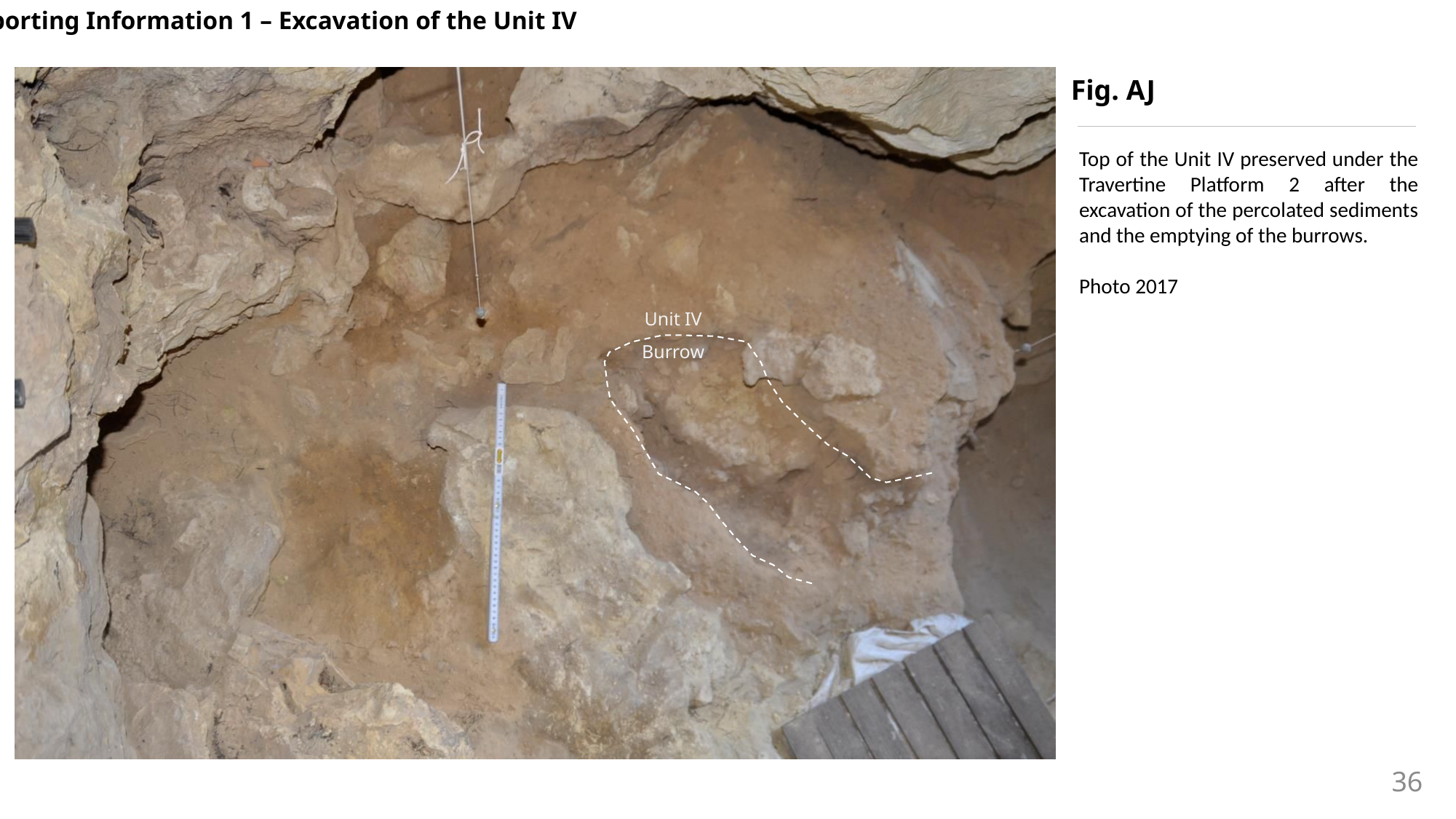

Supporting Information 1 – Excavation of the Unit IV
Fig. AJ
Top of the Unit IV preserved under the Travertine Platform 2 after the excavation of the percolated sediments and the emptying of the burrows.
Photo 2017
Unit IV
Burrow
36

## Slide 37
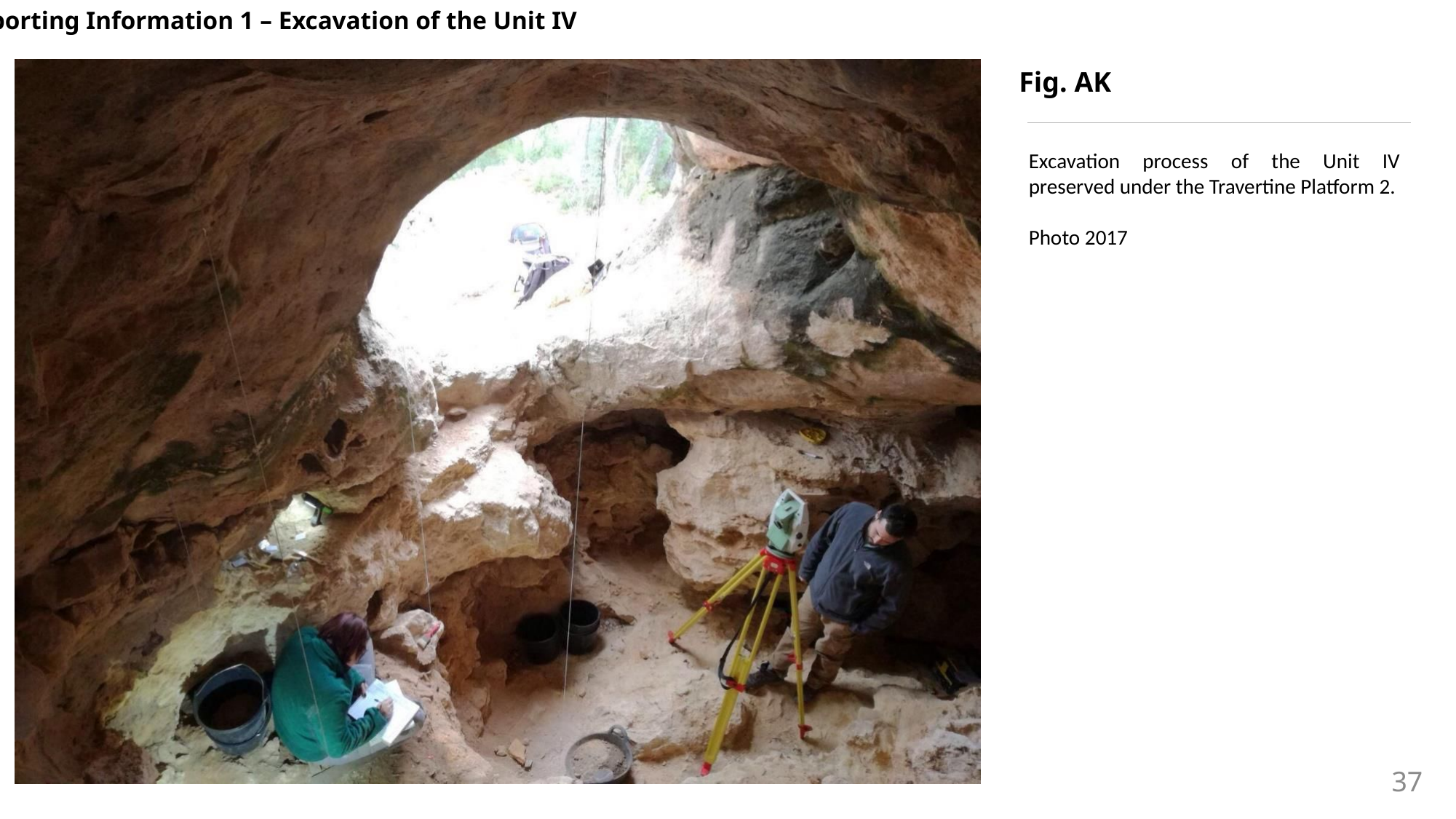

Supporting Information 1 – Excavation of the Unit IV
Fig. AK
Excavation process of the Unit IV preserved under the Travertine Platform 2.
Photo 2017
37

## Slide 38
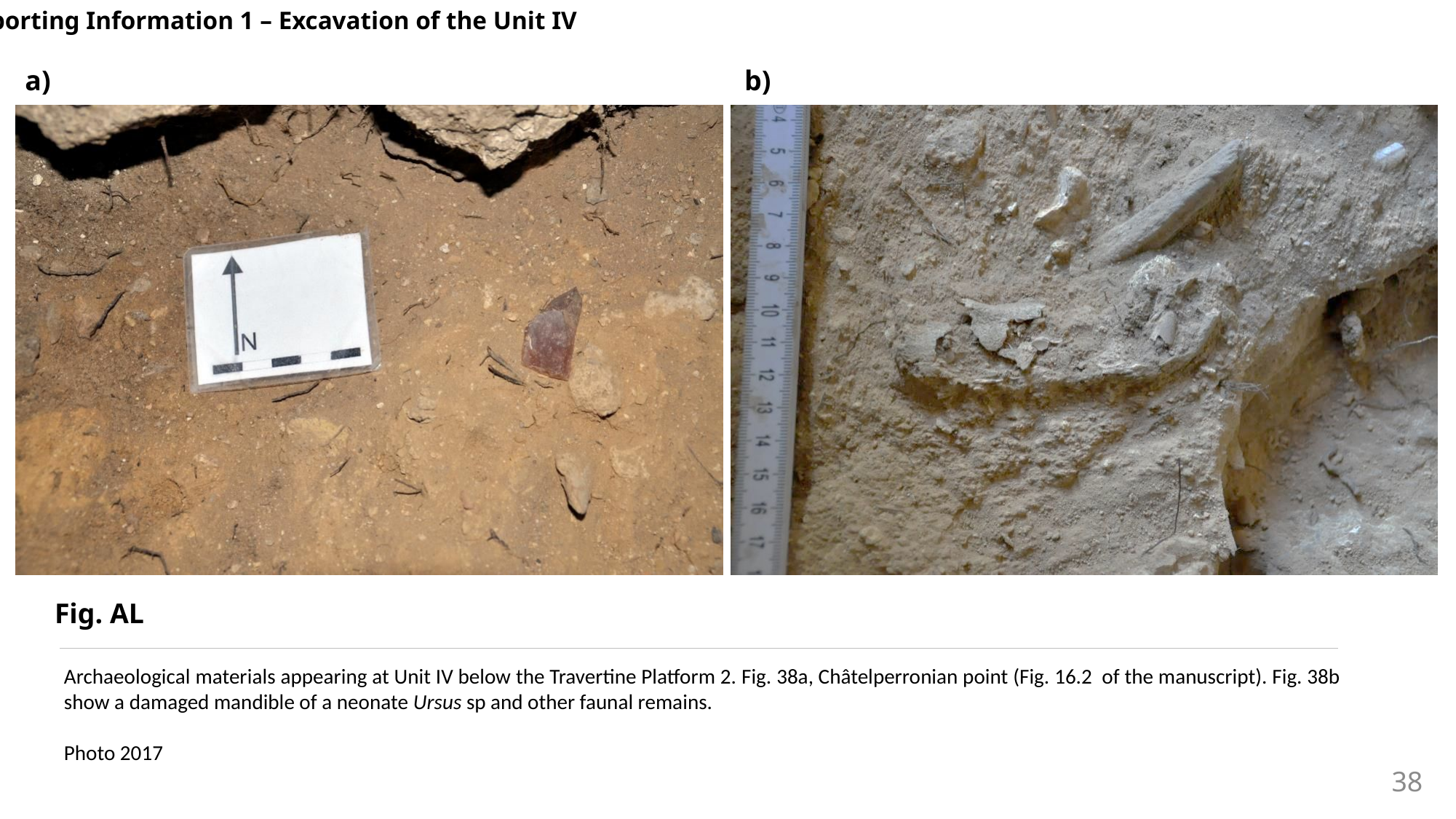

Supporting Information 1 – Excavation of the Unit IV
a)
b)
Fig. AL
Archaeological materials appearing at Unit IV below the Travertine Platform 2. Fig. 38a, Châtelperronian point (Fig. 16.2 of the manuscript). Fig. 38b show a damaged mandible of a neonate Ursus sp and other faunal remains.
Photo 2017
38

## Slide 39
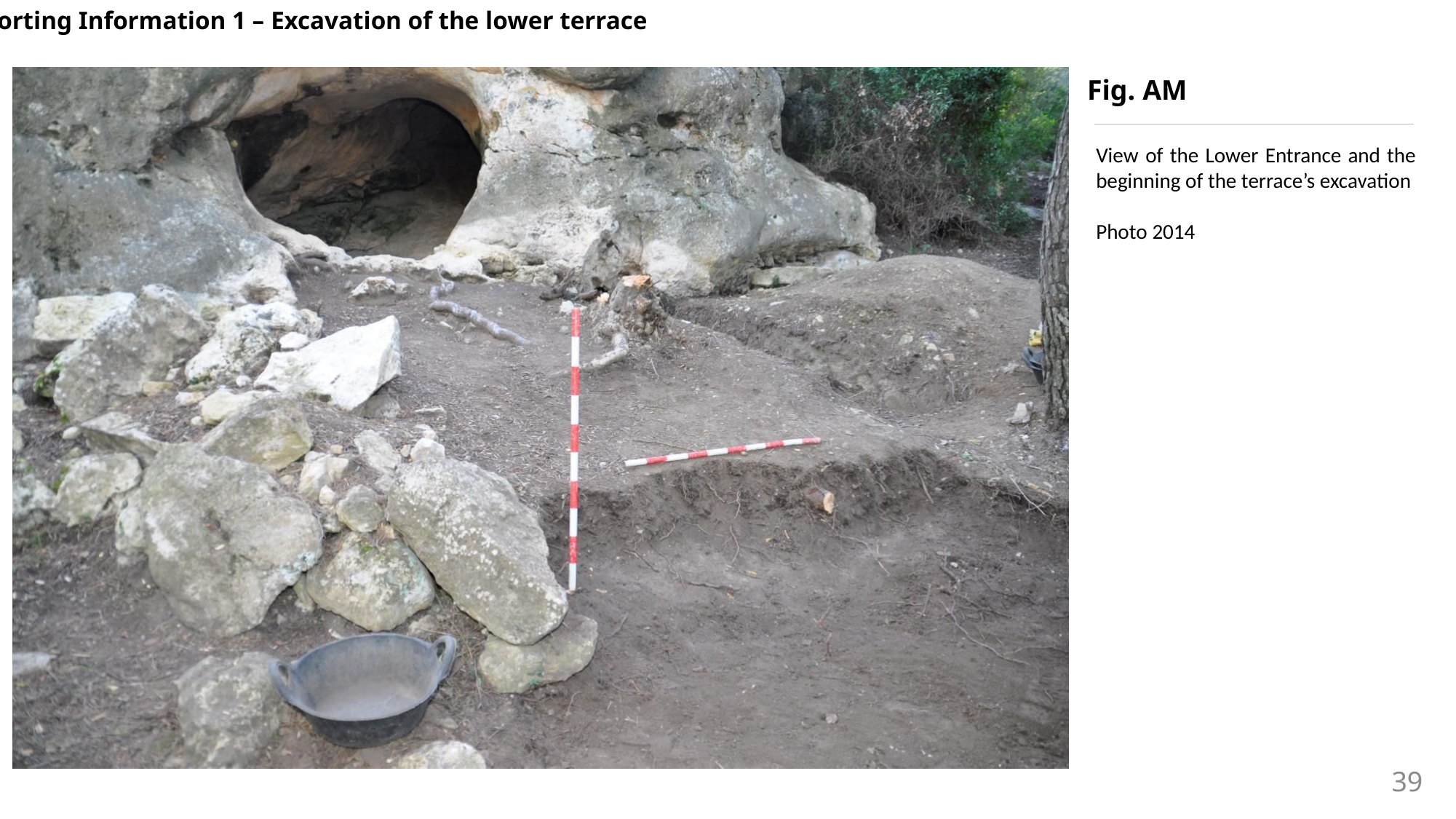

Supporting Information 1 – Excavation of the lower terrace
Fig. AM
View of the Lower Entrance and the beginning of the terrace’s excavation
Photo 2014
39

## Slide 40
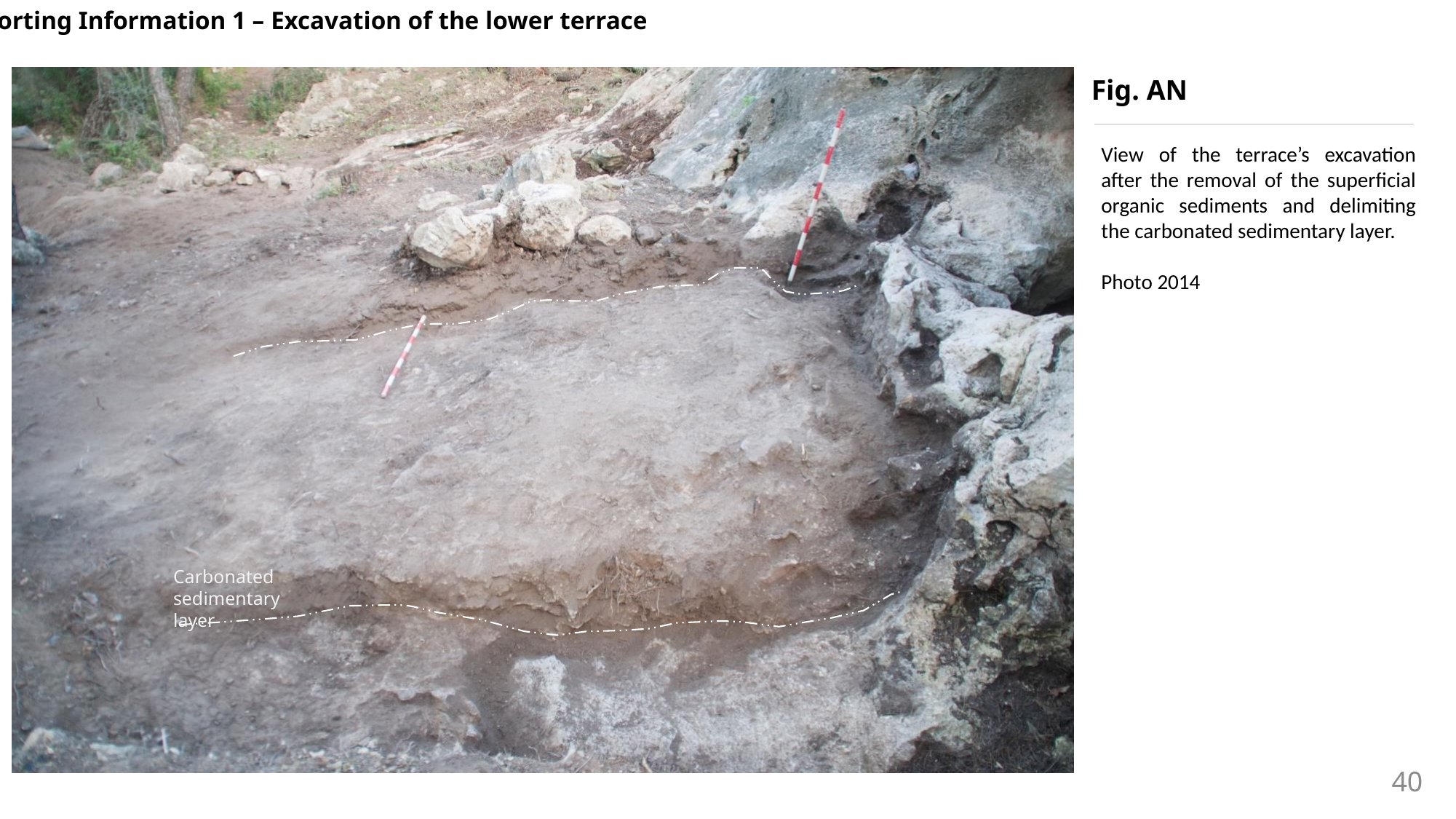

Supporting Information 1 – Excavation of the lower terrace
Fig. AN
View of the terrace’s excavation after the removal of the superficial organic sediments and delimiting the carbonated sedimentary layer.
Photo 2014
Carbonated sedimentary layer
40

## Slide 41
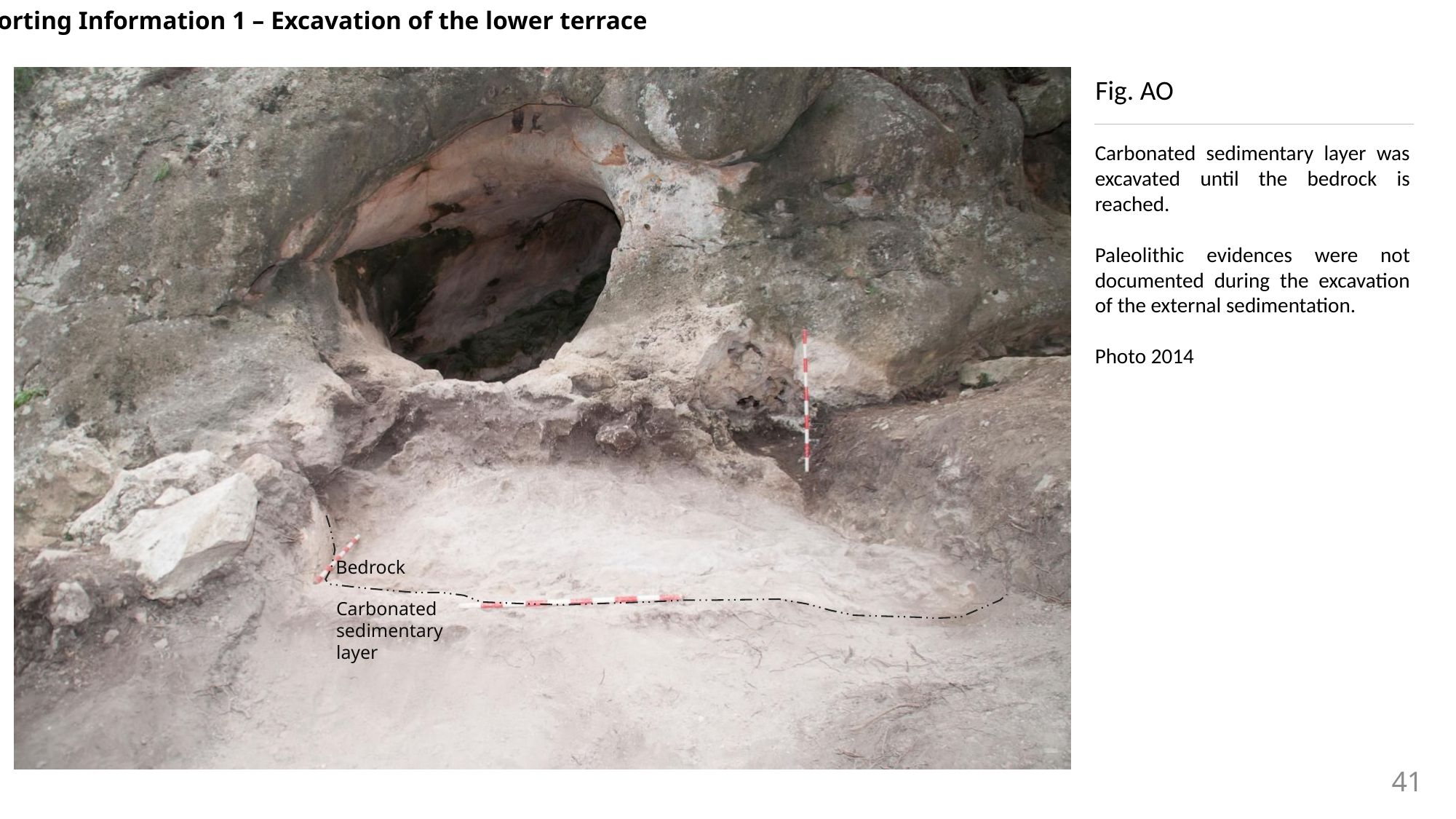

Supporting Information 1 – Excavation of the lower terrace
Fig. AO
Carbonated sedimentary layer was excavated until the bedrock is reached.
Paleolithic evidences were not documented during the excavation of the external sedimentation.
Photo 2014
Bedrock
Carbonated sedimentary layer
41

## Slide 42
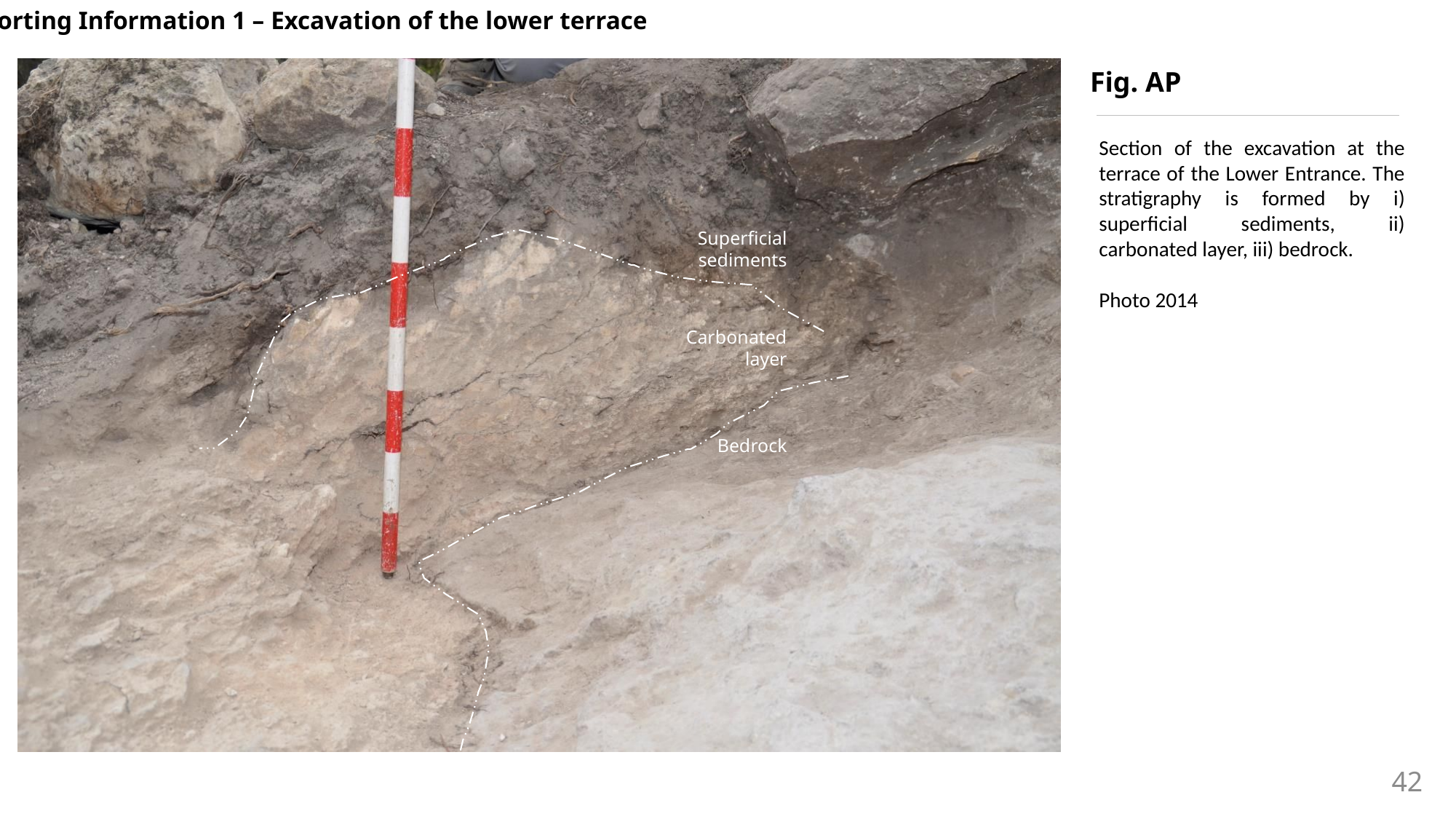

Supporting Information 1 – Excavation of the lower terrace
Fig. AP
Section of the excavation at the terrace of the Lower Entrance. The stratigraphy is formed by i) superficial sediments, ii) carbonated layer, iii) bedrock.
Photo 2014
Superficial sediments
Carbonated layer
Bedrock
42

## Slide 43
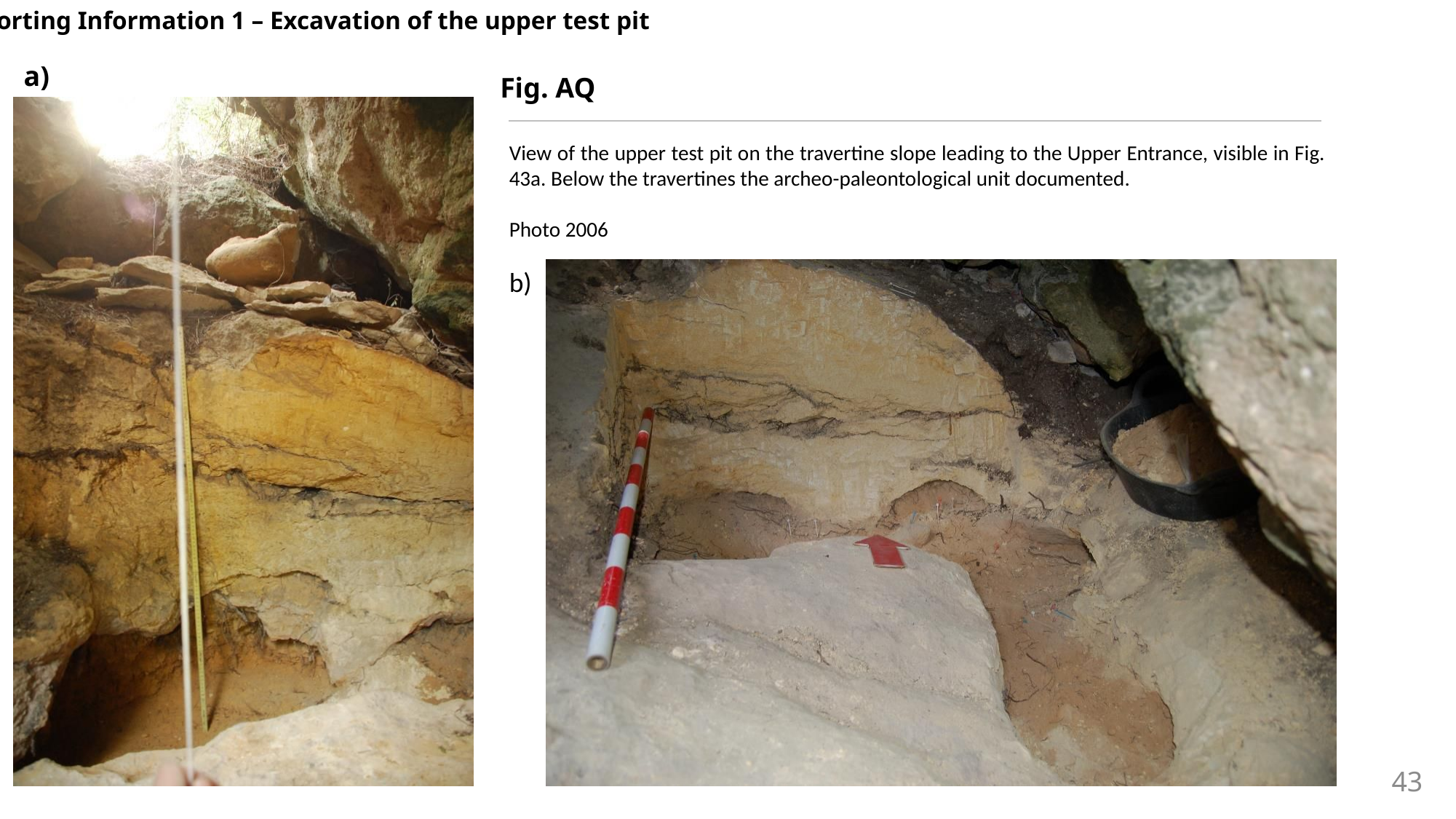

Supporting Information 1 – Excavation of the upper test pit
a)
Fig. AQ
View of the upper test pit on the travertine slope leading to the Upper Entrance, visible in Fig. 43a. Below the travertines the archeo-paleontological unit documented.
Photo 2006
b)
43

## Slide 44
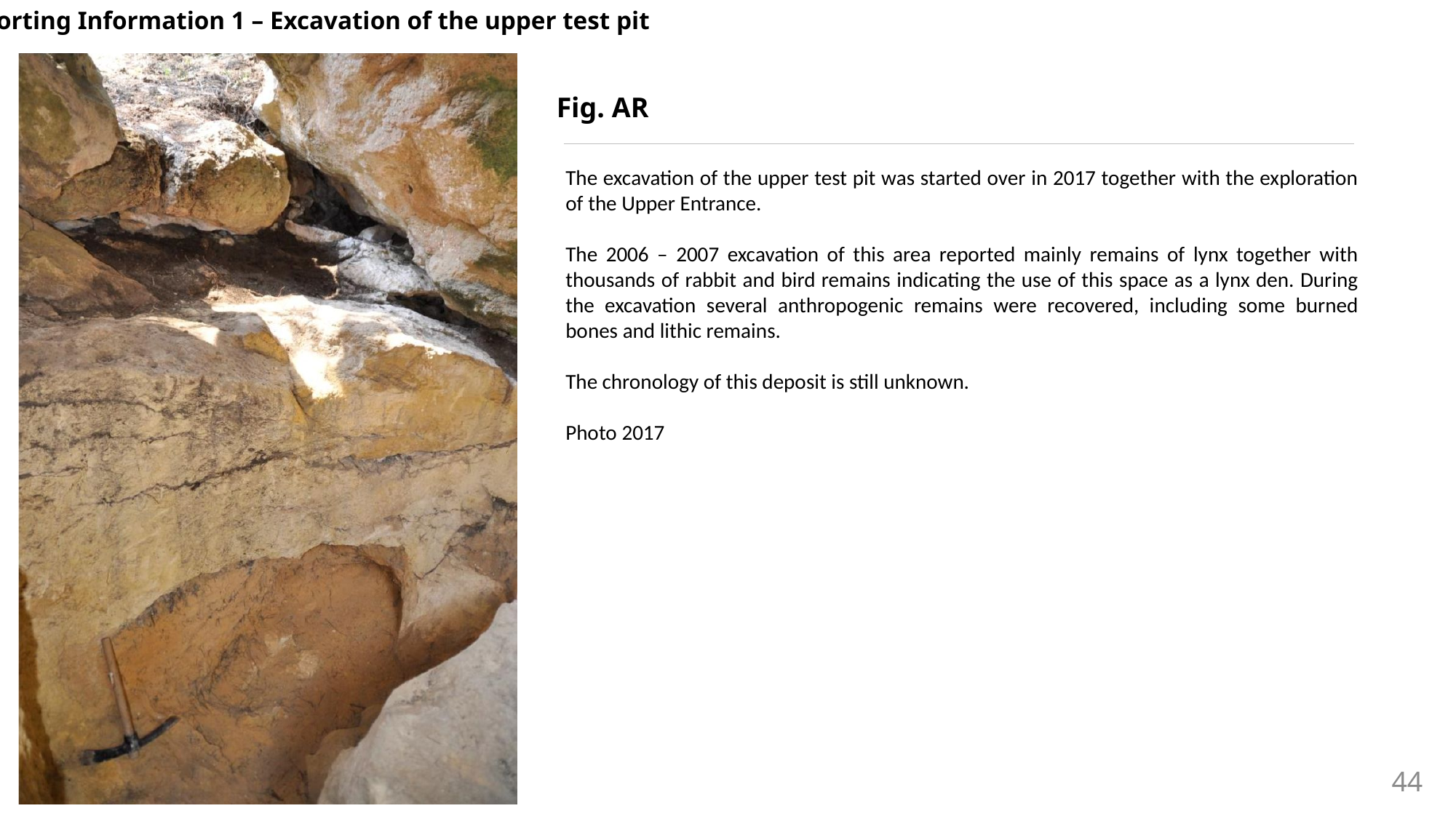

Supporting Information 1 – Excavation of the upper test pit
Fig. AR
The excavation of the upper test pit was started over in 2017 together with the exploration of the Upper Entrance.
The 2006 – 2007 excavation of this area reported mainly remains of lynx together with thousands of rabbit and bird remains indicating the use of this space as a lynx den. During the excavation several anthropogenic remains were recovered, including some burned bones and lithic remains.
The chronology of this deposit is still unknown.
Photo 2017
44

## Slide 45
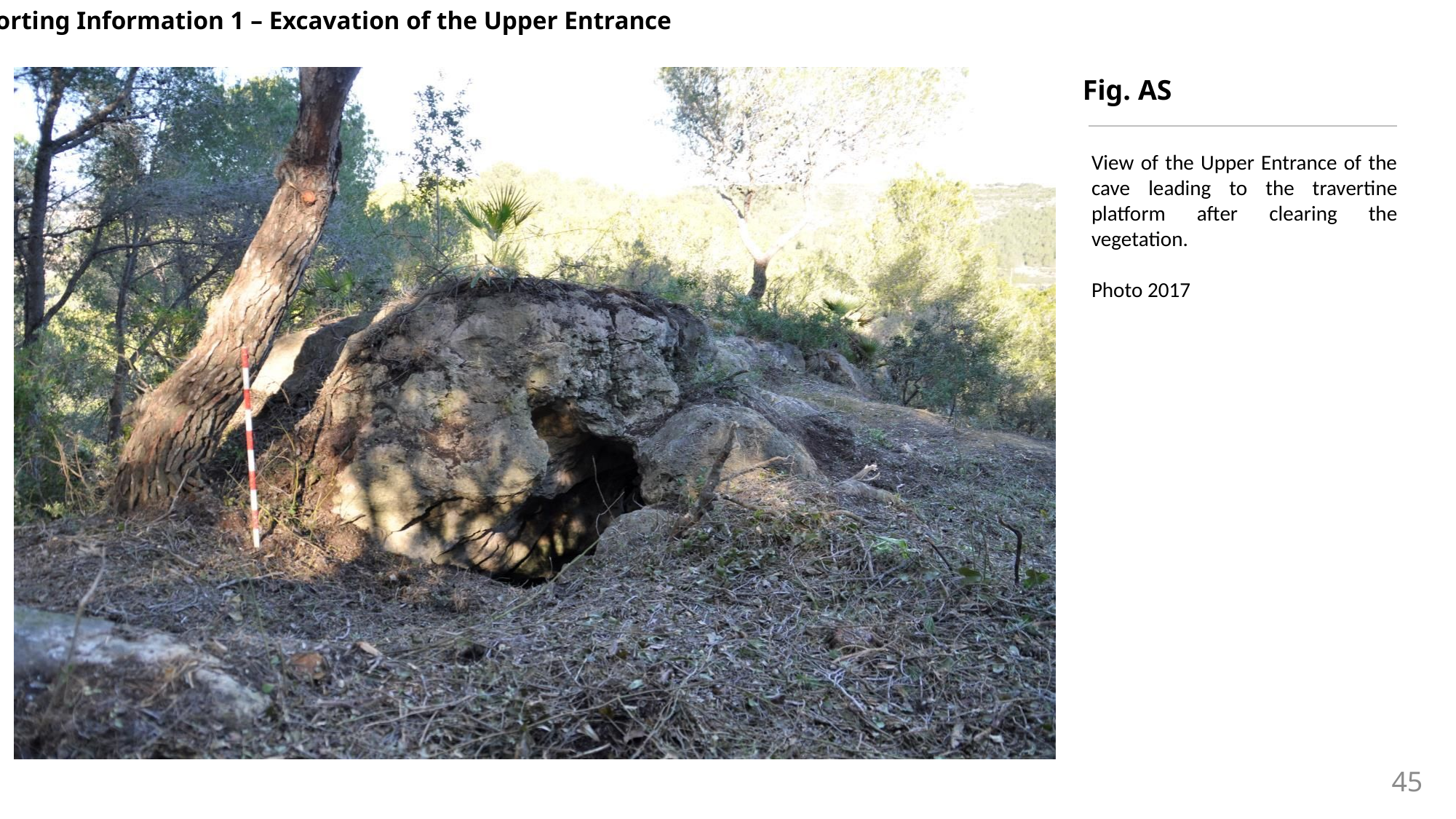

Supporting Information 1 – Excavation of the Upper Entrance
Fig. AS
View of the Upper Entrance of the cave leading to the travertine platform after clearing the vegetation.
Photo 2017
45

## Slide 46
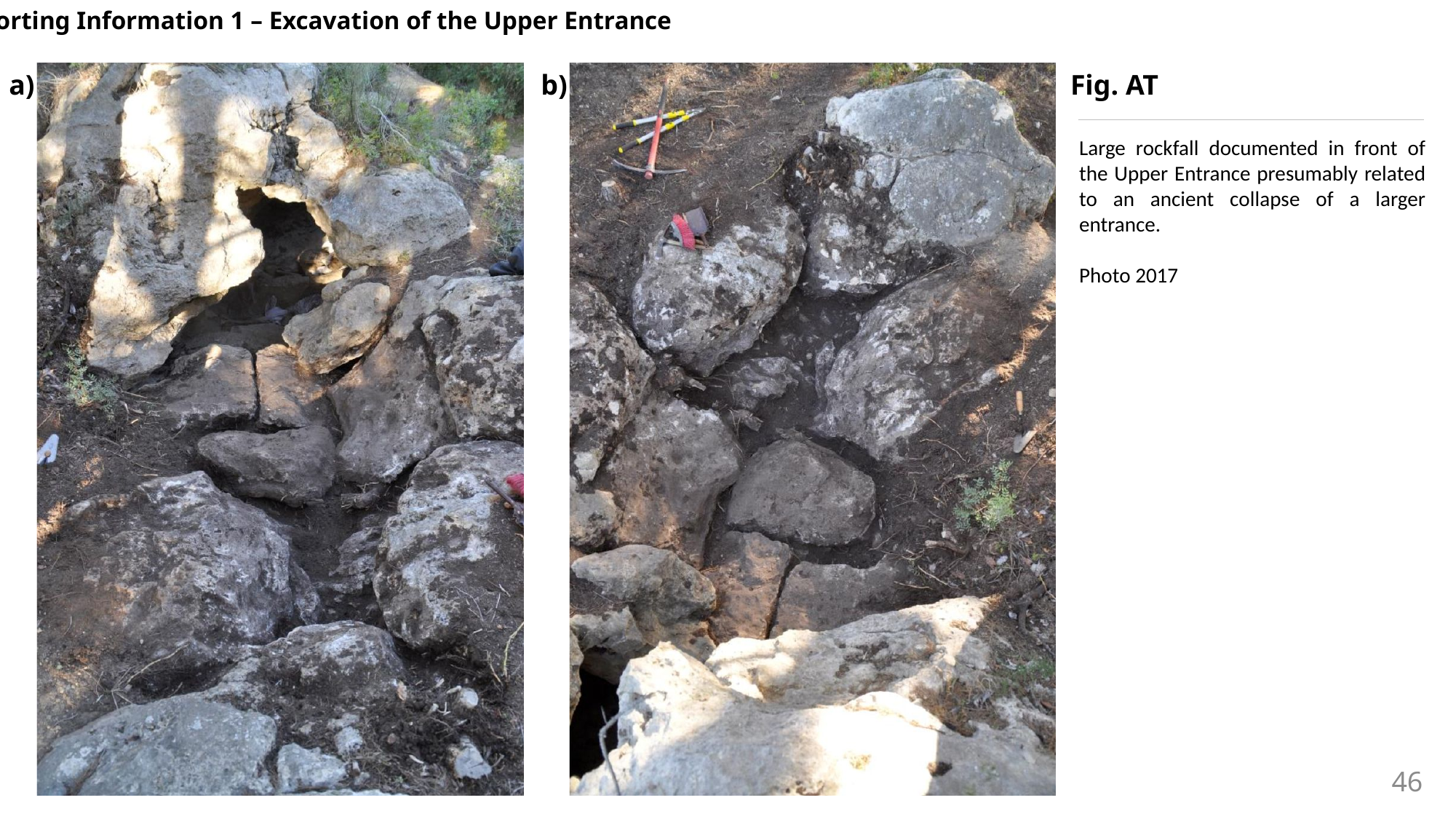

Supporting Information 1 – Excavation of the Upper Entrance
a)
b)
Fig. AT
Large rockfall documented in front of the Upper Entrance presumably related to an ancient collapse of a larger entrance.
Photo 2017
46

## Slide 47
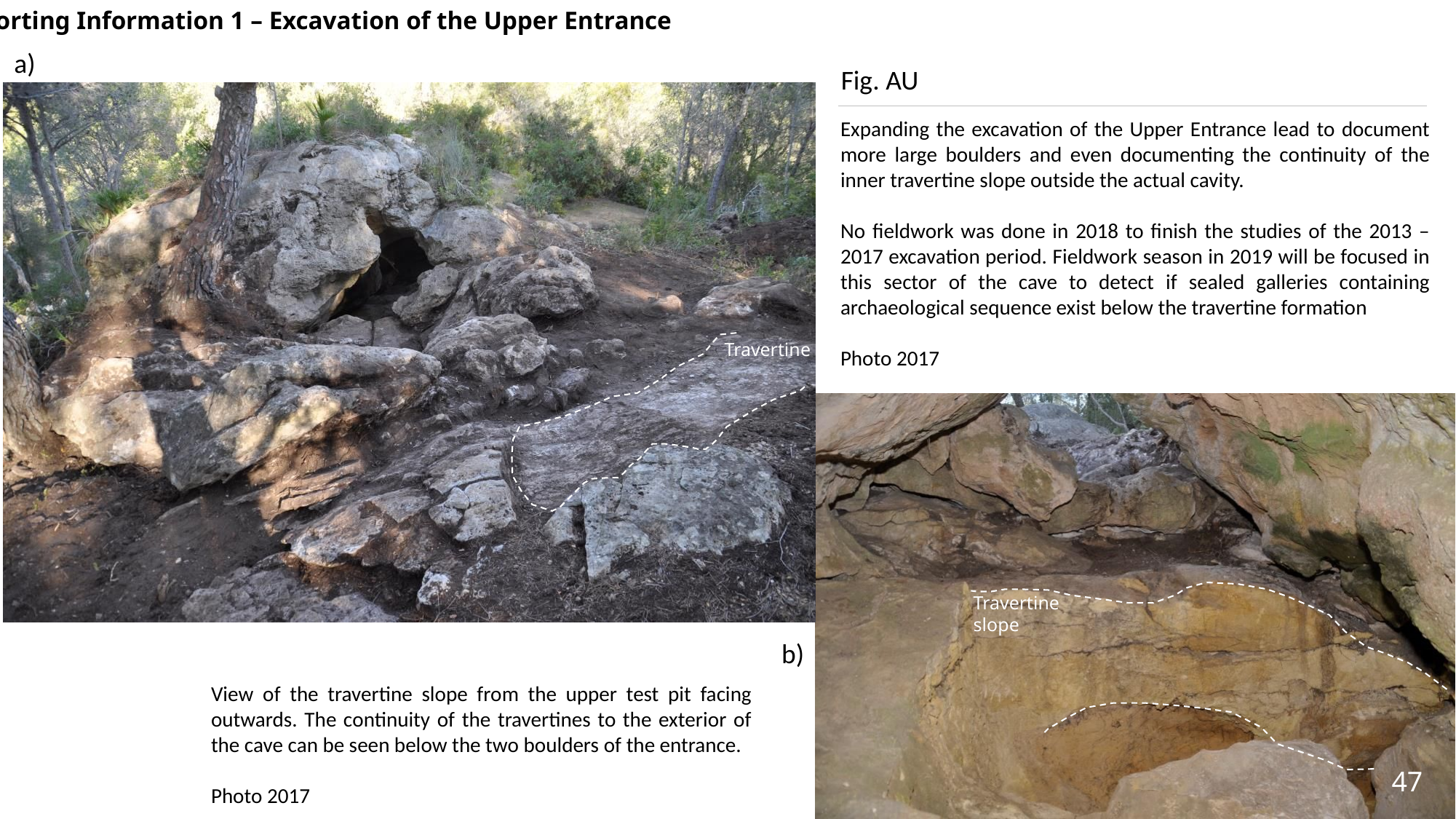

Supporting Information 1 – Excavation of the Upper Entrance
a)
Fig. AU
Expanding the excavation of the Upper Entrance lead to document more large boulders and even documenting the continuity of the inner travertine slope outside the actual cavity.
No fieldwork was done in 2018 to finish the studies of the 2013 – 2017 excavation period. Fieldwork season in 2019 will be focused in this sector of the cave to detect if sealed galleries containing archaeological sequence exist below the travertine formation
Photo 2017
Travertine
Travertine slope
b)
View of the travertine slope from the upper test pit facing outwards. The continuity of the travertines to the exterior of the cave can be seen below the two boulders of the entrance.
Photo 2017
47
47
