# Supplementary material for: The Middle-to-Upper Paleolithic transition occupations from Cova Foradada (Calafell, NE Iberia)
Source: PLoS One. 2019 May 16;14(5):e0215832. doi: 10.1371/journal.pone.0215832 (PMC6522054; doi:10.1371/journal.pone.0215832)
Supplement: S2 Supporting Information — (DOCX) [file pone.0215832.s002.docx]

Plot()

{

Outlier_Model("General",T(5),U(0,4),"t");

Sequence("Cova Foradada")

{

Boundary("Start Châtelperronian")

{

color="Red";

};

{

Phase("Châtelperronian")

{

R_Date("OxA-X-2650-9", 34300, 1000)

{

Outlier(0.05);

};

R_Combine("Subsample 6a and 6b")

{

Outlier(0.05);

R_Date("OxA-X-2649-9", 34490, 320);

R_Date("Beta-435465", 34570, 240);

};

};

Boundary("End Châtelperronian")

{

color="Red";

};

Boundary("Start Early Aurignacian")

{

color="Green";

};

Phase("Early Aurignacian")

{

R_Date("MAMS-33909", 30760, 150)

{

Outlier(0.05);

};

R_Date("Beta-378800", 30220, 180)

{

Outlier(0.05);

};

};

Boundary("End Early Aurignacian")

{

color="Green";

};

Boundary("Start Gravettian")

{

color="Blue";

};

Phase("Gravettian")

{

R_Date("OxA-24646", 26570, 120)

{

Outlier(0.05);

};

};

Boundary("End Gravettian")

{

color="Blue";

};

};

};

};
