# Supplementary material for: The Middle-to-Upper Paleolithic transition occupations from Cova Foradada (Calafell, NE Iberia)
Source: PLoS One. 2019 May 16;14(5):e0215832. doi: 10.1371/journal.pone.0215832 (PMC6522054; doi:10.1371/journal.pone.0215832)
Supplement: S3 Supporting Information — (DOCX) [file pone.0215832.s003.docx]

|  | Unmodelled (BP) | | | Modelled (BP) | | | Indices Amodel 108.3 Aoverall 108.5 | | | | |
| --- | --- | --- | --- | --- | --- | --- | --- | --- | --- | --- | --- |
|  |  |  |  |  |  |  |  |  |  |  |  |
|  |  |  |  |  |  |  |  |  |  |  |  |
|  | **from** | **to** | **%** | **from** | **to** | **%** | **Acomb** | **A** | **L** | **P** | **C** |
| Boundary End Layer IIIn |  |  |  | 31067 | 27196 | 95,4 |  |  |  |  | 98,5 |
| R_Date OxA-24646 | 31045 | 30610 | 95,4 | 31058 | 30605 | 95,4 |  | 100,8 |  | 95,9 | 99,9 |
| Phase Layer IIIn |  |  |  |  |  |  |  |  |  |  |  |
| Boundary Start Layer IIIn |  |  |  | 33650 | 30665 | 95,4 |  |  |  |  | 99,5 |
| Boundary End Layer IIIc |  |  |  | 34665 | 31939 | 95,4 |  |  |  |  | 99,6 |
| R_Date Beta-378800 | 34620 | 33902 | 95,4 | 34684 | 33945 | 95,4 |  | 96 |  | 95,8 | 99,9 |
| R_Date MAMS-33909 | 35009 | 34341 | 95,4 | 34973 | 34285 | 95,4 |  | 97,4 |  | 95,8 | 99,9 |
| Phase Layer IIIc |  |  |  |  |  |  |  |  |  |  |  |
| Boundary Start Layer IIIc |  |  |  | 37526 | 34298 | 95,4 |  |  |  |  | 99,4 |
| Boundary End Layer IV |  |  |  | 39426 | 35696 | 95,4 |  |  |  |  | 99,1 |
| R_Combine Subsample 6a and 6b | 39566 | 38581 | 95,4 | 39540 | 38545 | 95,4 |  | 102,0 |  | 95,4 | 99,8 |
| R_Date OxA-X-2650-9 | 41138 | 36445 | 95,4 | 40272 | 37278 | 95,4 |  | 127,2 |  | 95,7 | 99,8 |
| Phase Layer IV |  |  |  |  |  |  |  |  |  |  |  |
| Boundary Start Layer IV |  |  |  | 42226 | 38536 | 95,4 |  |  |  |  | 95,8 |
| Sequence Cova Foradada |  |  |  |  |  |  |  |  |  |  |  |
| U(0,4) | 3,990E-12 | 4 | 95,4 | 5,38E-12 | 3.808 | 95,4 |  | 100 |  |  | 99,8 |
| T(5) | -2,65 | 2,65 | 95,4 |  |  |  |  |  |  |  | 98,7 |
| Outlier_Model General |  |  |  | -1022 | 911 | 95,4 |  |  |  |  | 99,9 |
