# Supplementary material for: The Middle-to-Upper Paleolithic transition occupations from Cova Foradada (Calafell, NE Iberia)
Source: PLoS One. 2019 May 16;14(5):e0215832. doi: 10.1371/journal.pone.0215832 (PMC6522054; doi:10.1371/journal.pone.0215832)
Supplement: S1 Table — Percentage of lithics analyzed through high-power optical and digital microscopes and percentages of tools displaying any kind of use-related polishes. Hafting wear and post-depositional damage are not considered in the results. (DOCX) [file pone.0215832.s004.docx]

|  |  | Analyzed | | with use wear traces | | |
| --- | --- | --- | --- | --- | --- | --- |
|  | **Total lithics** | total | % | total | %  (from the analyzed sample) | %  (from the total) |
| Layer IIIn | 39 | 14 | 35,90 | 10 | 71,43 | 25,64 |
| Layer IIIc | 75 | 24 | 32,0 | 16 | 66,67 | 21,33 |
| Unit IV | 34 | 15 | 44,12 | 9 | 60,00 | 26,47 |
