# Supplementary figures and images for: The Middle-to-Upper Paleolithic transition occupations from Cova Foradada (Calafell, NE Iberia)
Source: PLoS One. 2019 May 16;14(5):e0215832. doi: 10.1371/journal.pone.0215832 (PMC6522054; doi:10.1371/journal.pone.0215832)

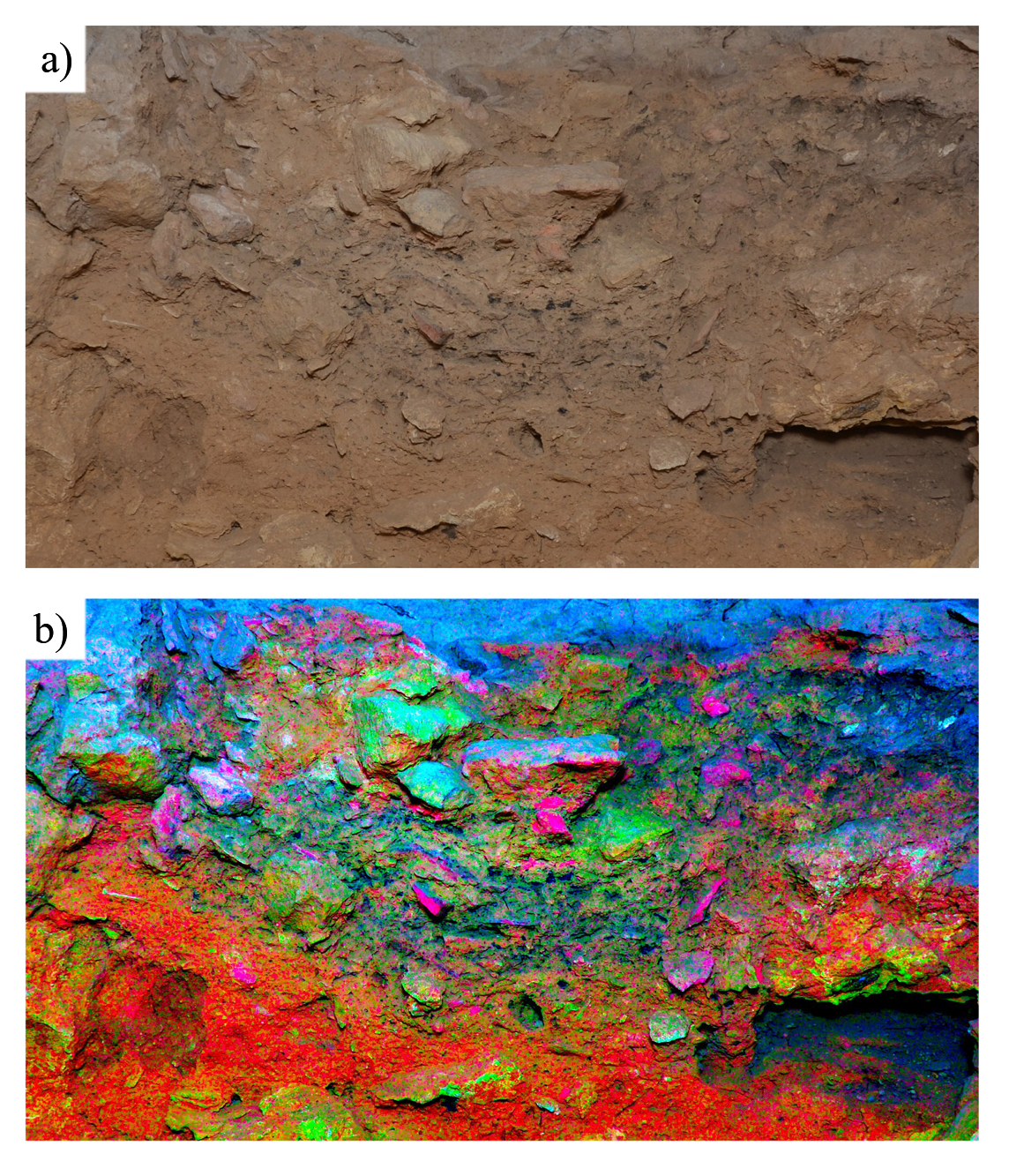

Supplement: S1 Fig — Detail of the stratigraphic contact between Units III and IV at the E9-D9 reference section (Fig 10A–10B and Fig 12 of the main text). a) View of the E9-D9 section, b) view of the E9-D9 section after image enhancement applying decorrelation stretch with the DStretch plugin for ImageJ (http://www.dstretch.com/), LXX colorspace. The enhancement clearly highlights the stratigraphic boundary between layer IIIc and the top of Unit IV (red). It also picks out combustion features from layer IIIc by identifying charcoal lines (blue) and altered sediments (magenta). (JPG) [file pone.0215832.s005.jpg]

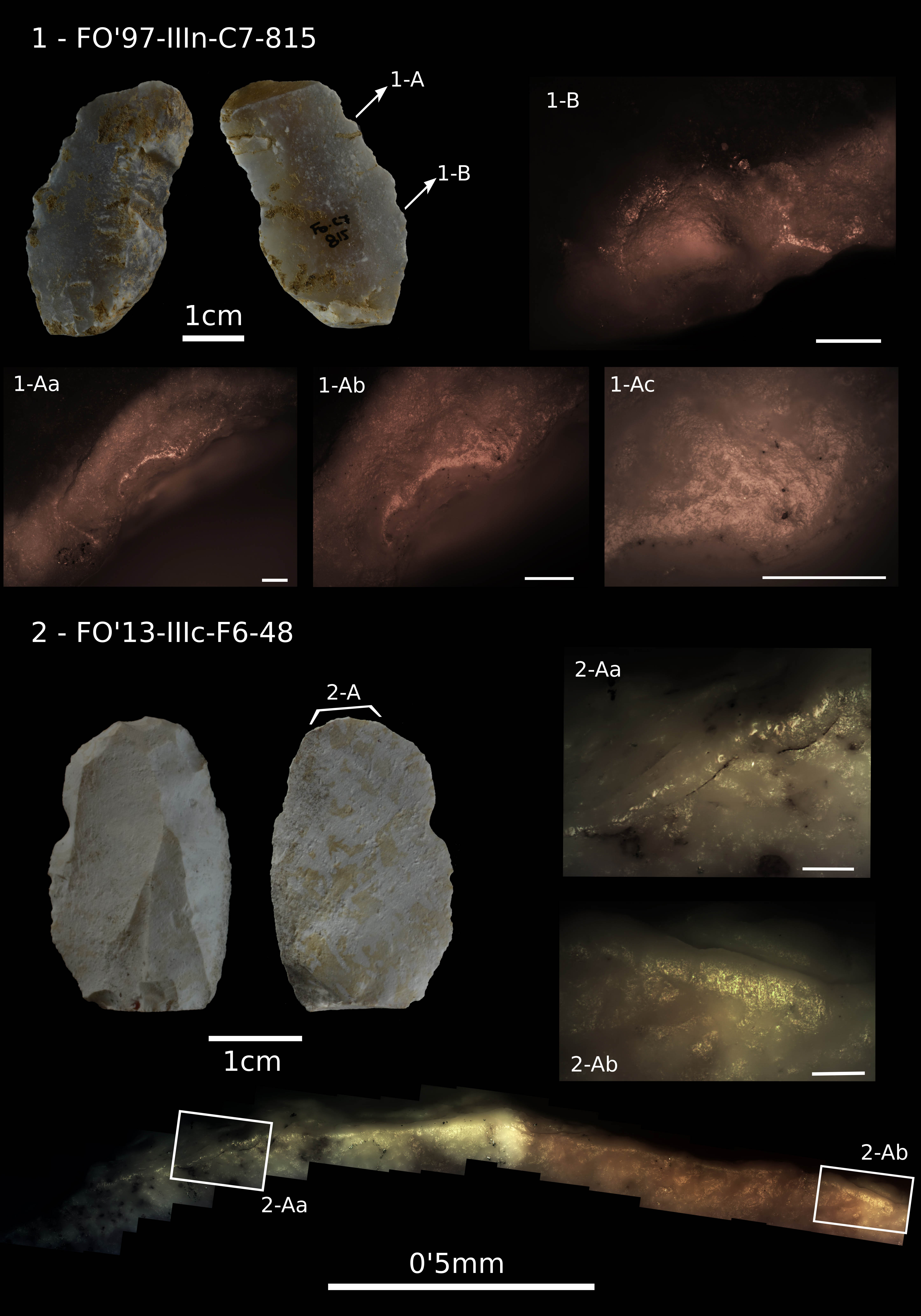

Supplement: S2 Fig — Scale bar represents 100μm in the microscope photos. 1. FO’97-IIIn-C7-815, denticulated flake with working traces of a medium-hard material, probably wood. The tool shows microchipping and intense polish and rounding along the edge on both sides. 1-Aa (100x), 1-Ab and 1B (200x) and 1-Ac (500x). 2. FO’13-III-F6-48. Scraper from the Aurignacian layer with intense rounding and polish provoked by its use on hard materials, possibly bone or antler. 2-A is a “gigapixel-like” image of the edge with more intense traces. 2-Aa shows the most intense polish, and 2Ab shows rounding, polish, and striation-like features. All images of this tool were taken at 200x. (JPG) [file pone.0215832.s006.jpg]

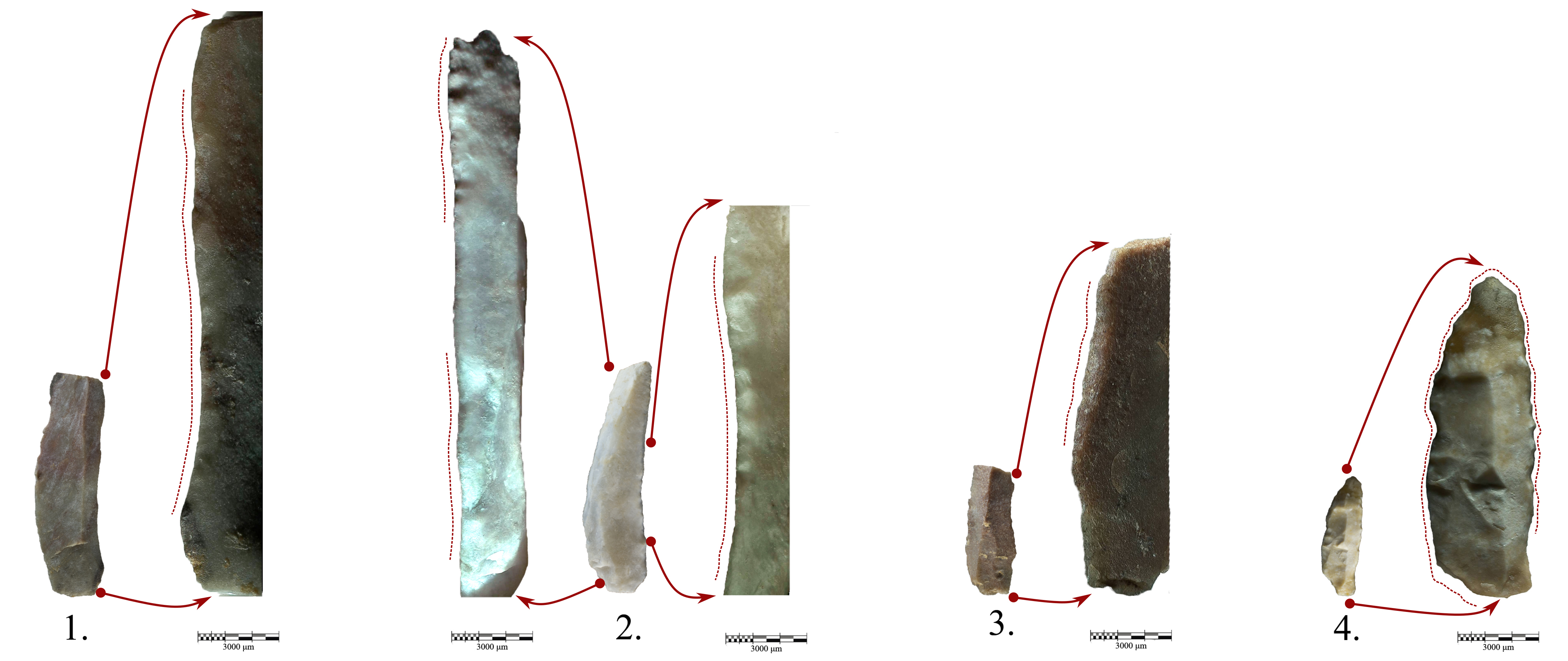

Supplement: S3 Fig — Details of the marginally retouched edges of the bladelets from layer IIIc (Fig 13 3,4,8–10 of the main text). Panoramic views of the retouched edges have been processed using a KH8700 Hirox digital microscope at the IPHES lithic analysis laboratory. Scales are only applicable to magnified edges, numbered bladelets are in a relative scale. (JPG) [file pone.0215832.s007.jpg]

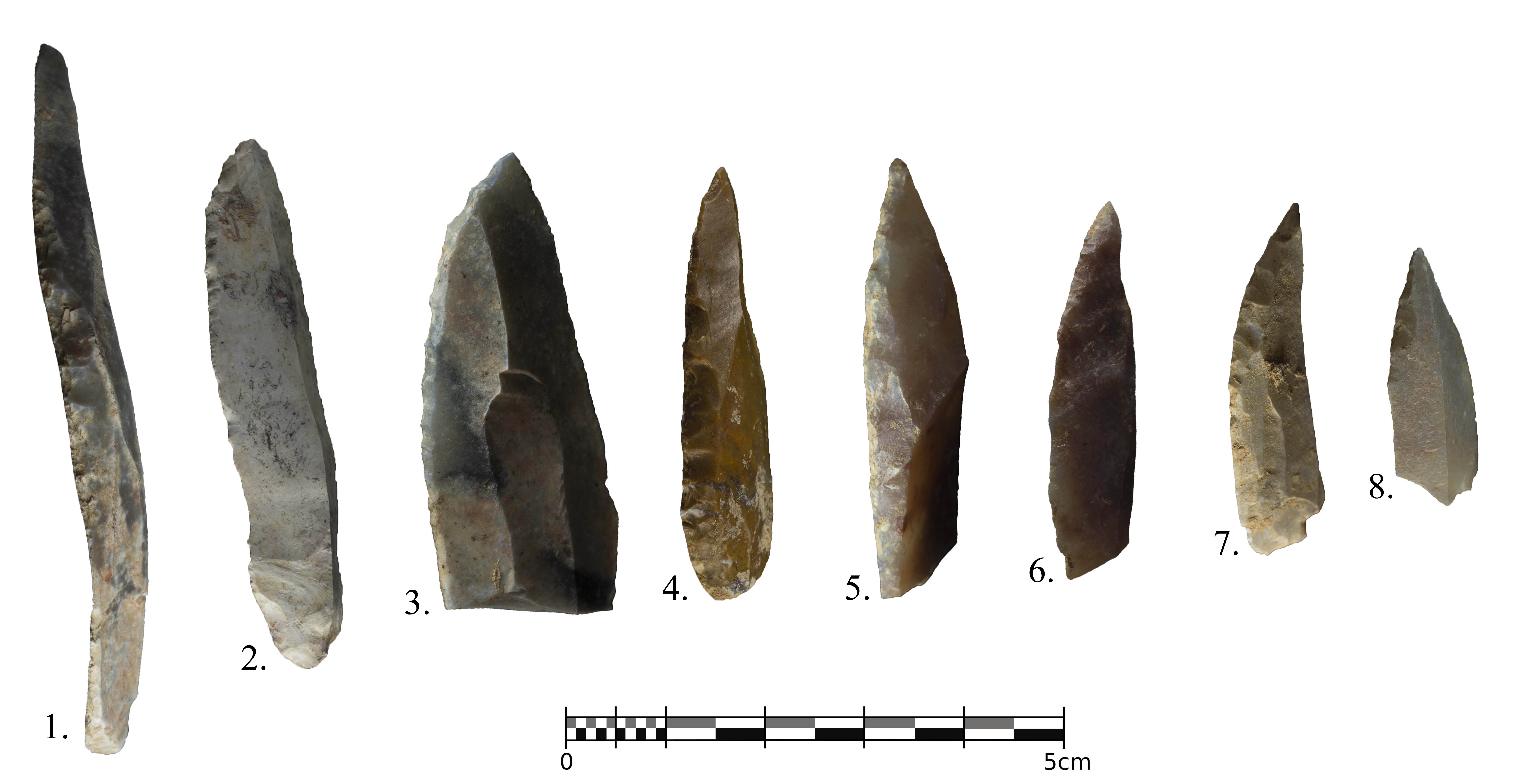

Supplement: S4 Fig — Comparative lateral views of the extent and distribution of retouching on the retouched blades from Unit IV. Lateral views of blades 1, 2, 3 and 8 have been mirrored for comparative purposes. (JPG) [file pone.0215832.s008.jpg]

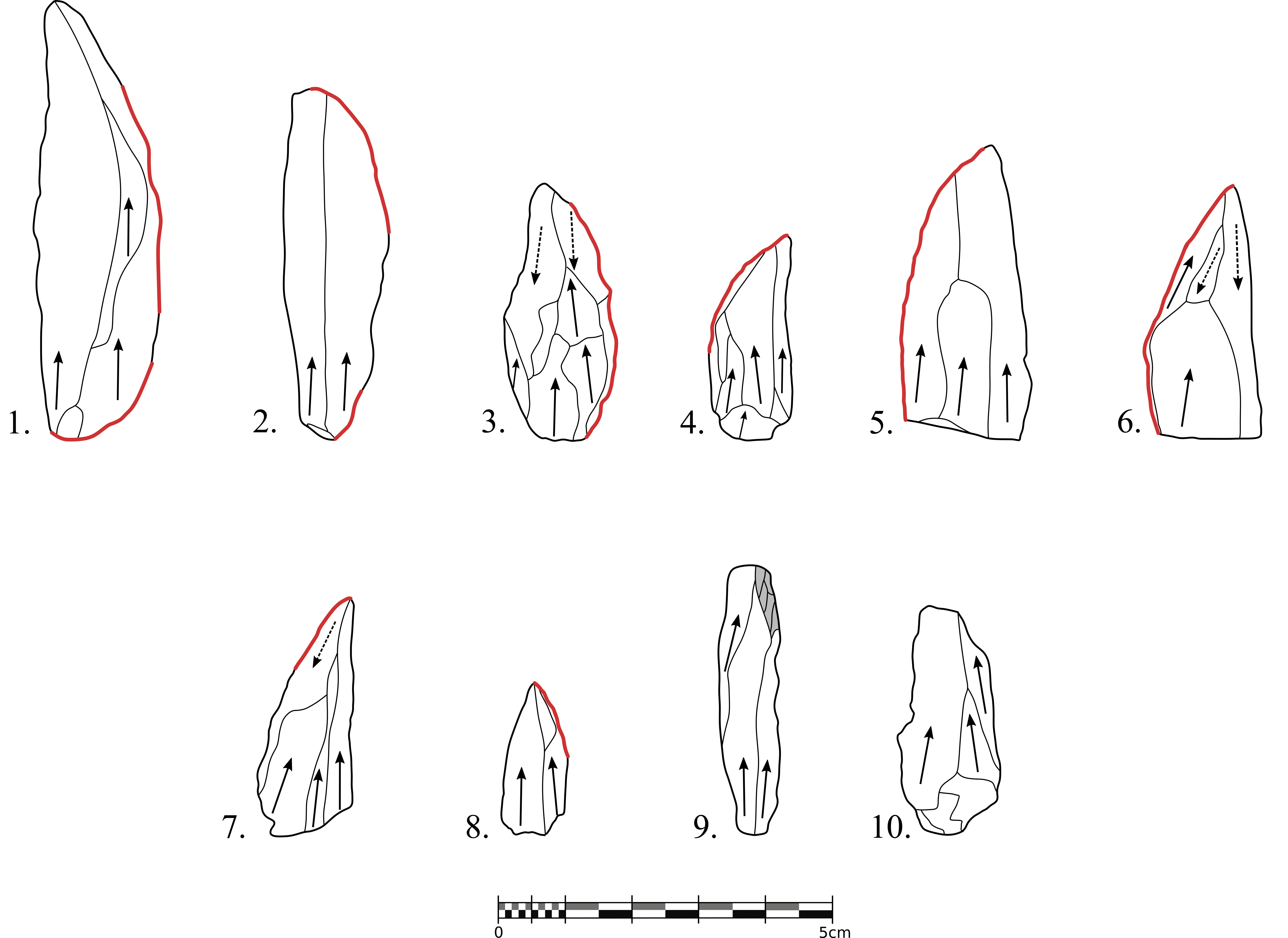

Supplement: S5 Fig — 1–8 retouched blades or Châtelperronian points. 9–10 unretouched blades. (JPG) [file pone.0215832.s009.jpg]
